# 3D printer emissions elicit filament-specific and dose-dependent metabolic and genotoxic effects in human airway epithelial cells

**DOI:** 10.3389/fpubh.2024.1408842

**Published:** 2024-07-12

**Authors:** LMA Barnett, Q. Zhang, S. Sharma, S. Alqahtani, J. Shannahan, M. Black, C. Wright

**Affiliations:** ^1^Chemical Insights Research Institute, UL Research Institutes, Marietta, GA, United States; ^2^School of Health Sciences, Purdue University, West Lafayette, IN, United States; ^3^Advanced Diagnostic and Therapeutics Technologies Institute, Health Sector, King Abdulaziz City for Science and Technology (KACST), Riyadh, Saudi Arabia

**Keywords:** 3D printer, emissions, exposure dosimetry, particulate matter, respiratory toxicity, metabolomics

## Abstract

Three-dimensional (3D) printers have become popular educational tools in secondary and post-secondary STEM curriculum; however, concerns have emerged regarding inhalation exposures and associated health risks. Current evidence suggests that filament materials and site conditions may cause differences in the chemical profiles and toxicological properties of 3D printer emissions; however, few studies have evaluated exposures directly in the classroom. In this study, we monitored and sampled particulate matter (PM) emitted from acrylonitrile-butadiene-styrene (ABS) and polylactic acid (PLA) filaments during a 3-hour 3D printing session in a high school classroom using aerosol monitoring instrumentation and collection media. To evaluate potential inhalation risks, Multiple Path Particle Dosimetry (MPPD) modeling was used to estimate inhaled doses and calculate *in vitro* concentrations based on the observed aerosol data and specific lung and breathing characteristics. Dynamic light scattering was used to evaluate the hydrodynamic diameter, zeta potential, and polydispersity index (PDI) of extracted PM emissions dispersed in cell culture media. Small airway epithelial cells (SAEC) were employed to determine cellular viability, genotoxic, inflammatory, and metabolic responses to each emission exposure using MTS, ELISA, and high-performance liquid chromatography-mass spectrometry (HPLC-MS), respectively. Aerosol monitoring data revealed that emissions from ABS and PLA filaments generated similar PM concentrations within the ultrafine and fine ranges. However, DLS analysis showed differences in the physicochemical properties of ABS and PLA PM, where the hydrodynamic diameter of PLA PM was greater than ABS PM, which may have influenced particle deposition rates and cellular outcomes. While exposure to both ABS and PLA PM reduced cell viability and induced MDM2, an indicator of genomic instability, PLA PM alone increased gamma-H2AX, a marker of double-stranded DNA breaks. ABS and PLA emissions also increased the release of pro-inflammatory cytokines, although this did not reach significance. Furthermore, metabolic profiling via high performance liquid chromatography-mass spectrometry (HPLC-MS) and subsequent pathway analysis revealed filament and dose dependent cellular metabolic alterations. Notably, our metabolomic analysis also revealed key metabolites and pathways implicated in PM-induced oxidative stress, DNA damage, and respiratory disease that were perturbed across both tested doses for a given filament. Taken together, these findings suggest that use of ABS and PLA filaments in 3D printers within school settings may potentially contribute to adverse respiratory responses especially in vulnerable populations.

## Introduction

Fused filament fabrication (FFF) is a form of three-dimensional (3D) printing that employs heating and extrusion of thermoplastics in layers onto a print bed surface to form multi-dimensional objects. FFF has become the most common form of 3D printing and is a popular hands-on educational tool in secondary and higher education settings. However, these benefits are coupled with potential health hazards due to the release of potentially harmful emissions during 3D printing.

3D printers pose potential respiratory hazards to users because they emit ultrafine particles at rates of 2 × 10^8^ to 2 × 10^12^ min^−1^ in tandem with gas phase emissions (1–4). This is concerning because ultrafine particles can cause both local and systemic toxicity by penetrating deep into the respiratory tract, passing through the alveolar–capillary barrier, and distributing throughout the body (5). Additionally, 3D printers emit metals such as Cr, As, Pb, Cd, and Co (6–8) and volatile organic compounds (VOCs) such as styrene, formaldehyde, acetaldehyde, ethylbenzene, methylene chloride, methyl-methacrylate, toluene, lactide, and caprolactam that are International Agency for Research on Cancer (IARC) class 1 or 2 carcinogens and/or respiratory hazards (1–3, 9–12). Moreover, total VOC and individual VOCs released by 3D printers have been shown to exceed national and international indoor air quality (IAQ) standards (10, 11). This is especially concerning for indoor environments that are poorly filtered and ventilated, such as older homes, schools, and small offices.

Importantly, the chemical composition of 3D printing emissions depends on printer settings and filament formulations. For example, acrylonitrile butadiene styrene (ABS) filaments have been shown to emit 3 to 4-fold higher emissions than polylactic acid (PLA) filaments (1, 2, 6, 13). This could be because ABS filaments require higher extrusion temperatures relative to PLA filaments and contain unknown additives that elevate emissions (4, 14). Accordingly, regardless of the filament type (ABS vs. PLA), higher extrusion temperatures have been shown to increase particle and VOC emissions from 3D printers (15, 16).

Conversely, relatively few studies have compared different printer settings and filament formulations in terms of their toxicological effects. A recent *in vitro* study from Zhang and coworkers revealed that ABS and PLA 3D printer emission exposures caused a reduction in cell viability and oxidative stress in both macrophages and airway epithelial cells (4). Farcas and coworkers revealed dose-dependent increases in pro-inflammatory cytokine and chemokines, oxidative stress, and cytotoxicity due to ABS emission exposures in small airway epithelial cells (17). Animal studies investigating 3D printing emissions have also found concerning results where 3 h exposures to 1 mg/m^3^ of ABS aerosols induced substantial impairments to cardiovascular function in rats (18). Moreover, a recent health survey revealed approximately 60% of participants using 3D printers in commercial prototyping facilities, educational settings, and public libraries experienced weekly respiratory issues along with strong associations between hours worked per week and asthma or allergic rhinitis development (19). Given the rising popularity of 3D printers in educational and residential settings, research on how 3D printer emissions may alter indoor air quality and enhance exposure to hazardous chemicals is critical to protecting human health.

In this study, we characterized the particulate emissions and potential respiratory toxicity resulting from a 3 h 3D printing session at a high school and compared two different filament types, ABS and PLA. Scanning mobility particle sizer (SMPS) and optical particle sizer (OPS) technology were used to compare particle sizes and concentrations and dynamic light scattering (DLS) was used to determine physicochemical properties of ABS and PLA particles including hydrodynamic diameter and zeta potential. Dosimetric analyses using multiple-path particle dosimetry (MPPD) computational software were performed to estimate rate of deposition of 3D printer emissions within the human lung using parameters obtained during aerosol monitoring. Potentially inhaled doses and extrapolated *in vitro* concentrations were calculated using aerosol data consisting of count median diameter, geometric standard deviation, and aerosol concentration along with breathing parameters. Additionally, primary small airway epithelial cells (SAEC) were exposed to ABS and PLA-emitted particles collected and extracted from filters for 24 h, followed by assessments of cell viability, DNA damage, inflammation, and metabolomic responses. Our results suggest that ABS and PLA 3D printing emissions reduce cellular viability, induce genotoxic effects, and elicit metabolic changes in SAEC. Furthermore, metabolic pathways related to oxidative stress, DNA damage, inflammation, and respiratory disease were altered by ABS and PLA across both tested doses. Ultimately, these results advance our understanding of the potential toxicity of 3D printer emissions and their impact on respiratory health.

## Methods

### Sampling sites and generation of 3D printer emissions

Airborne particulate matter (PM) was collected from a high school located in Atlanta, GA. There were two locations studied for each filament material; one science, technology, engineering, and mathematics (STEM) lab classroom with a 3D printer (hereinafter referred to as the printer room) and an adjacent classroom without a 3D printer (the control room). In the printer room, PM was sampled within one meter of the printer.

One fused filament fabrication (FFF) 3D printer was operated in the printer room for 3 h to generate a cube. Black ABS (extrusion temperature = 245°C, printer chamber temperature = 85°C) or black PLA (extrusion temperature = 200°C, printer chamber temperature = 40°C) filaments were used on two separate days. On each day, PM was monitored during printing in both the printer room and control room. Support filaments (SR30 for ABS at 240°C and PVA (polyvinyl alcohol) for PLA at 200°C) were loaded to enable printer function following manufacturer’s instruction and accounted for a minimal fraction of the printed part.

### PM monitoring, sampling, and extraction

Aerosol size distributions were assessed using a scanning mobility particle sizer (NanoScan SMPS, TSI 3910) and an optical particle sizer (OPS, TSI 3330) to detect a particle size range of 10 nm to 10 microns. Fine PM (PM_2.5_, less than 2.5 μm) were collected during printing using PTFE (polytetrafluoroethylene) filters (37 mm, 0.45 μm pore size), personal modular impactors, and portable pumps at a flow rate of 4 L/min. The weight of PM collected on the filter was analyzed using a microbalance (Mettler Toledo XS3DU) by subtracting pre-sampling filter weights from post-sampling filter weights. Filter collected particles were extracted using a solvent-based (75% methanol) method coupled with sonication. Extractions were then concentrated using a vacufuge to remove the solvent extraction fluid and refrigerated until toxicological analysis.

### Estimation of inhaled and *in vitro* doses

Estimated inhaled doses were determined by inputting measured aerosol data into the Multiple-Path Particle Dosimetry 2 (MPPD2) computational model. The parameters used by MPPD2 to calculate deposition comprise four areas: the type of airway morphometry was age-specific (14 years old) symmetric; the particle properties included count median diameter (CMD), geometric standard deviation (GSD), and averaged aerosol mass concentration; the exposure was a constant exposure at the measured PM concentration. The exposure time was assumed to be 6 h per day, 5 days per week for a school year of 36 weeks. The total deposited mass across the airways (Generations 1–21) was divided by the surface area of those regions, which provided the total deposited dose within the small airways. To convert the total deposited dose to an *in vitro* dose or concentration, the total deposited dose (μg/cm^2^) was multiplied by the surface area of one well within a 96 well plate (0.33 cm^2^) then divided by the total exposure volume (100 μL).

### Dynamic light scattering

Extracted particles were submerged in cell culture media and analyzed on a Zetasizer Ultra (Malvern Panalytical, Malvern, United Kingdom). Samples were loaded into folded capillary cells/cuvettes (Malvern Panalytical, DTS1070) and polystyrene cells/cuvettes (Malvern Panalytical, DTS0012) for analysis of zeta potential and particle size, respectively.

### Cell culture and 3D printer PM exposure parameters

Normal small airway epithelial cells (SAEC) were cultured in small airway basal media (SABM) (Lonza, Walkersville, MD) supplemented with bovine pituitary extract (BPE), hydrocortisone, human epidermal growth factor (hEGF), epinephrine, transferrin, insulin, retinoic acid, triiodothyronine, gentamicin, and amphotericin-B (GA-1000). SAEC were maintained in a humidified atmosphere of 37°C and 5% CO_2_ with media renewal every 2–3 days. For 3D printer exposure assessments, SAEC were seeded in 96 well plates at a density of 10,000 cells/well and grown to 70–80% confluency for 5–7 days. Cells were exposed to 5 μg/mL or 10 μg/mL of PM extracts from the printer room and control room for 24 h to cover the range of estimated *in vitro* doses from the MPPD2 model (Table 1). PM extracts were diluted in cell culture media and administered in a volume of 100 μL in triplicate for each dose. In addition, untreated cells in culture media were used as a negative control (NC).

**Table 1:** Aerosol characterization, estimated inhaled doses, and calculated *in vitro* doses of particles emitted during 3 h of 3D printing.

Sample location	Count median diameter (nm)	Geometric standard deviation	Aerosol concentration (μg/m^3^)	Inhaled dose (μg/cm^2^)	*In vitro* dose (μg/mL)
Control room	125 ± 4.0	1.77 ± 0.12	4.22 ± 0.48	2.08	6.87
Printer room during ABS printing	87.4 ± 4.9	1.94 ± 0.24	4.33 ± 0.81	1.46	4.81
Printer room during PLA printing	130 ± 3.0	1.77 ± 0.32	4.44 ± 0.79	2.16	7.14

### MTS assay

Cell viability was measured after 24 h of exposure to PM using the CellTiter 96 Aqueous One Solution Cell Proliferation Assay (Promega Corp., Madison, WI). This test is based on the reduction of the tetrazolium salt MTS (3-[4,5-dimethylthiazol-2-yl]-5-(3-carboxymethoxyphenyl)-2-(4-sulfophenyl)-2H-tetrazolium) into a soluble purple formazan product by dehydrogenase enzymes in metabolically active cells. After removing the exposure media containing ABS or PLA PM, cells were washed twice with 1X phosphate buffered saline (PBS). A 1:10 dilution of MTS reagent: cell culture media was added to each well for 45 min and absorbance was read at 490 nm using a microplate reader (Cytation 1, Biotek). Triplicate readings were blank-corrected and averaged for each control and sample. In addition, cells that were treated with a hypotonic solution (0.1% Triton-X) served as a positive control (PC). Cells were also treated with blank filter extracts to account for residual solvent during the extraction process.

### Endotoxin assay

The Pierce Chromogenic Endotoxin Quant Kit (Pierce Biotechnology, Rockford, IL) was used to assess the potential for bacterial endotoxin contamination when cells were exposed to particulate matter samples. Due to limited sample, cell lysates were assessed rather than cell culture supernatants. All steps were performed according to the manufacturer’s protocol. Briefly, 50 μL lysates, standards, and blanks were added in triplicate to a 96-well plate. After adding 50 μL Amebocyte Lysate Reagent to each well, the plate was incubated at 37°C for the time indicated on the lysate vial. Next, 100 μL/well of Chromogenic Substrate Solution was added, followed by 6 min incubation at 37°C. To stop the reaction, 50 μL Stop Solution was added to each well. Absorbance was read at optical density (OD) 405 nm using a Cytation C10 plate reader (Agilent, Santa Clara, CA). The blank-corrected absorbance for standards and samples was calculated by subtracting the average absorbance of blank wells. The corresponding endotoxin concentration of each sample was calculated by plotting a standard curve of the average blank-corrected absorbance of each standard vs. the known endotoxin concentration in EU/mL.

### DNA damage evaluation

The MILLIPLEX 7-Plex DNA Damage/Genotoxicity Magnetic Bead Kit (Millipore Sigma) was used to measure changes in a panel of 7 DNA damage and repair pathway markers, including phosphorylated Chk1 (Ser 345), Chk2 (Thr68), H2A.X (Ser139), and p53 (Ser15) as well as total protein levels of ATR, MDM2, and p21. Following 24 h exposure to PM, SAEC were lysed, and protein was collected using mammalian protein extraction reagent (MPER, Thermo Fisher) according to the manufacturer’s protocol. Protein extracts were diluted to 1 mg/mL and analyzed according to the assay protocol. The Median Fluorescence Intensity (MFI) was measured on a Luminex Flexmap 3D system. Triplicate readings were blank-corrected and averaged for each control and sample.

### Cytokine analysis

Cytokines were detected and quantified in media collected from SAEC following 24 h exposure to PM using the Quantibody^®^ Human Cytokine Array (QAH-CYT-1) full testing ELISA service provided by Raybiotech Life, Inc. (Peachtree Corners, GA). Media samples were centrifuged at 250 × g for 1 min prior to cytokine analysis. A panel of 20 cytokines, including IL-1α, IL-1β, IL-2, IL-4, IL-5, IL-6, IL-8, IL-10, IL-12p70, IL-13, GM-CSF, GRO, IFNg, MCP-1, MIP-1α, MIP-1β, MMP-9, RANTES, TNFα, and VEGF, were analyzed. Quantibody^®^ employs matched pairs of antibodies for target protein detection in which multiple capture antibody arrays are printed on a standard slide. After blocking, unknown samples are incubated with the arrays, followed by a wash step to remove non-specific protein binding. A cocktail of biotinylated detection antibodies was then added to the array along with streptavidin-conjugated fluorescent reagents that were subsequently detected using a fluorescence laser scanner. Array-specific predetermined protein standards were utilized to generate an 8-point standard curve of each target protein. Concentrations of each cytokine were calculated in unknown samples using the standard curve and Q analyzer software.

### Metabolite profiling, sample preparation, and extraction

Protein removal and sample extraction were performed by adding 500 mL of methanol to 200 mL of cell supernatant. Solutions were vortexed and centrifuged at 16,000 g for 8 min. The supernatants were transferred to separate vials and evaporated to dryness in a vacuum concentrator. The dried polar fractions were reconstituted in 60 mL of diluent composed of 95% water and 5% acetonitrile, containing 0.1% formic acid.

### High performance liquid chromatography-mass spectrometry (HPLC-MS) and bioinformatic analyses

HPLC-MS and bioinformatic analyses were performed as described in our previous publication (20). Separations were performed on an Agilent 1,290 system (Palo Alto, CA), with a mobile phase flow rate of 0.45 mL/min. The metabolites were assayed using a Waters HSS T3 column (1.8 μm, 2.1 × 100 mm), where the mobile phases were A (0.1% formic acid in ddH2O) and B (0.1% formic acid in acetonitrile). Initial conditions were 100:0 A:B, held for 1 min, followed by a linear gradient of 80:20 at 16 min, then 5:95 at 21 min, held for 1.5 min. Column re-equilibration was performed by returning to 100:0 A:B at 23.5 min and holding until 28.5 min. The mass analysis was obtained in positive ionization mode using an Agilent 6,545 Q-TOF mass spectrometer with ESI capillary voltage +3.5 kV, nitrogen gas temperature 325°C, drying gas flow rate 8.0 L/min, nebulizer gas pressure 30 psig, fragmentor voltage 135 V, skimmer 45 V, and OCT RF 750 V. Mass data (from m/z 70–1,000) were collected using Agilent MassHunter Acquisition software (v. B.06). Mass accuracy was improved by infusing Agilent Reference Mass Correction Solution (G1969-85001). MS/MS was performed in a data dependent acquisition mode. Peak picking and annotation was performed using MS-DIAL (v. 4.7).^1^ Adduct ions selected were [M + H]+, [M + Na]+, [2 M + H]+, [2 M + Na]+. After blank peak removal, 1,141 sample related peaks were observed. Peak annotations were performed using the MassBank of North America metabolomics MS/MS library, based on authentic standards (v. 16).^2^ Mass tolerances were 0.005 Da for MS1 and 0.01 Da for MS2. Statistical analysis was performed using MetaboAnalyst 5.0.^3^ Data imputation, normalization, and comparisons were made with significance threshold set at *p* < 0.05.

### Pathway analysis

For each condition, a 4-column table of m/z features, *p*-values, *t* scores, and retention time was inputted into the MS Peaks to Pathways module on MetaboAnalyst 5.0 (see text footnote 3). The Mummichog algorithm was selected as the analysis parameter, and the human KEGG pathway library was selected. Significantly enriched pathways were selected with an adjusted *p* < 0.05.

### Statistical analysis

Results obtained from the MTS, DNA, and cytokine assays were assessed for statistical significance using one-way ANOVA followed by a Dunnett’s post-hoc analysis using GraphPad Prism version 10.1.2 (Boston, Massachusetts, United States) to compare each treatment group to the untreated negative control (NC). For metabolomics, all statistical analyses were performed using MetaboAnalyst 5.0 (see text footnote 3). The level of statistical significance was *p* < 0.05 for all analyses. HCA heatmaps were made using MetaboAnalyst 5.0. Venn diagrams were made using Venn Diagram Plotter version 1.6.7458.^4^ All other graphs were made using GraphPad Prism version 10.1.2.

## Results

### Indoor aerosol characterization and dosimetry

Aerosol characteristics, estimated inhaled and *in vitro* dose are described in Table 1. Details of particle number distribution are shown in Supplementary Figure S1. Particles emitted during ABS printing fell within the nanoscale range and were smaller, but more concentrated relative to particles in the control room. The estimated inhaled and *in vitro* deposited doses were smaller for ABS-emitted particles compared to particles in the control room, and this was likely because smaller-sized particles contributed to less mass deposition. On the other hand, particles in the PLA printing room were slightly larger and more concentrated than particles in the control room. The size, average concentration, and estimated inhaled and *in vitro* deposited doses of particles emitted in the printer room during ABS printing were consistently lower than the corresponding characteristics of particles emitted during PLA printing. This could be due to a combination of variables such as printer emission rate, local ventilation conditions, occupancy, and in-room activities that likely differed during the dates of sampling.

### Size, polydispersity, and surface charge of submerged PM samples

We performed dynamic light scattering (DLS) to assess the physical properties of PM upon submersion in cell culture media (Table 2). The size of PM from all sampling locations increased upon submersion in media, as indicated by hydrodynamic diameter or z-averages relative to the count median diameters summarized in Table 1. PLA PM had a greater hydrodynamic diameter compared to ABS PM. Additionally, PM from all sample locations had similar polydispersity, with control room PM being the most polydisperse. Finally, the surface charge of all PM was negative, with control room being the most negatively charged, followed by ABS, and then PLA.

**Table 2:** Characteristics of collected PM when submerged in SAEC media as measured by dynamic light scattering (DLS).

Sample location	Z-average (nm)	Polydispersity index (PDI)	Zeta potential (mV)
Control room	261.21 ± 24.52	0.546 ± 0.11	−21.556 ± 0.86
Printer room during ABS printing	1269.26 ± 33.02	0.444 ± 0.12	−18.824 ± 3.44
Printer room during PLA printing	1515.85 ± 205.53	0.502 ± 0.092	−13.411 ± 5.89

### Effect of 3D printer emissions on cellular viability

**Figure 1:**
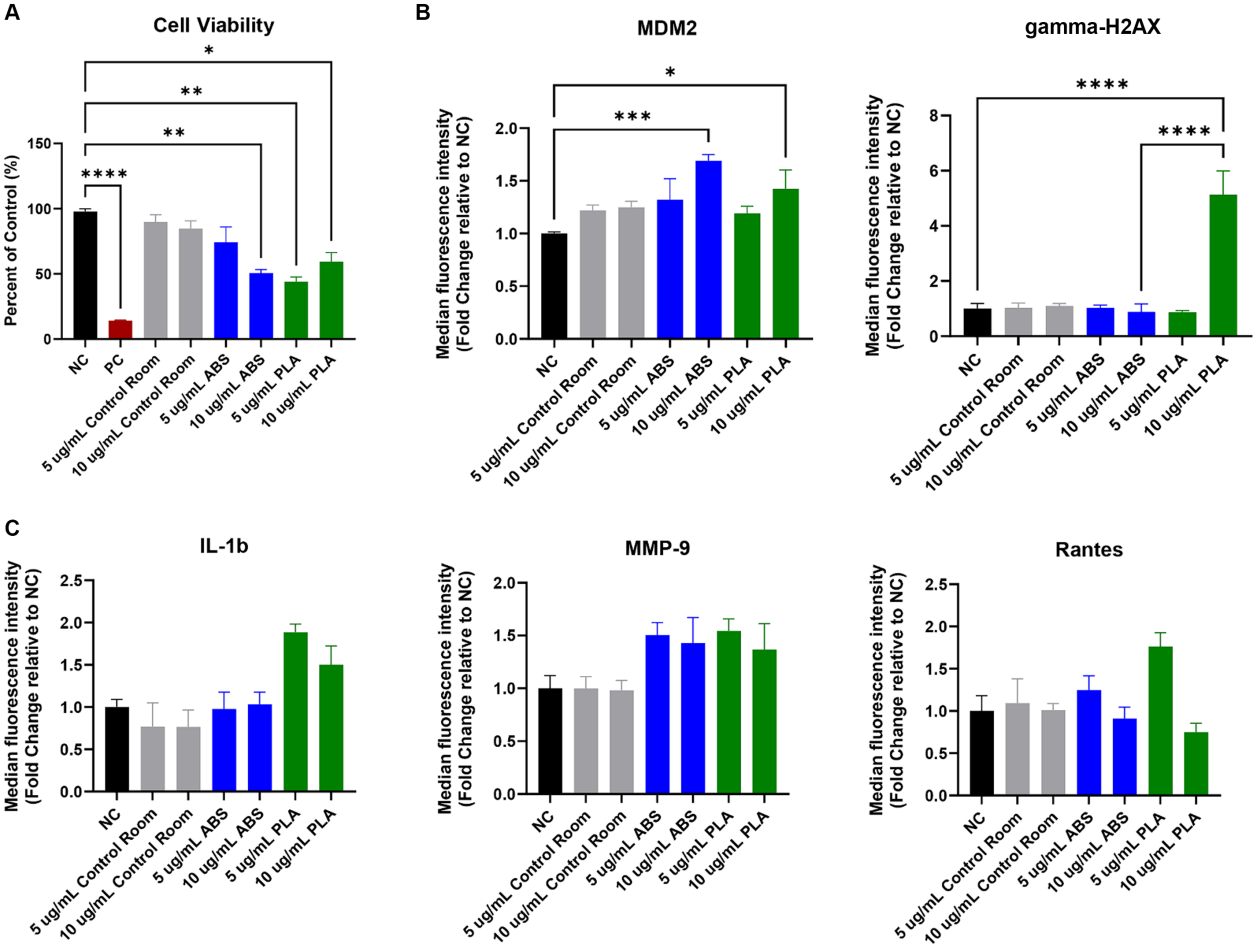
Toxicological effects of 24 h exposure to PM emitted during 3 h of 3D printing in SAEC. Each graph represents the effect of exposure to 5 μg/mL and 10 μg/mL PM collected during printing with ABS, PM collected during printing with PLA, or PM collected from the control room on **(A)** SAEC cell viability as measured by the MTS assay; **(B)** expression of DNA damage and repair markers; and **(C)** release of pro-inflammatory cytokines. Error bars represent the standard error of the mean. *n* = 3–4. *p < 0.05, **p < 0.01, ***p < 0.001, ****p < 0.0001 relative to untreated negative control cells (NC).

The MTS assay was used to determine the metabolic capacity and viability of SAEC after 24 h of exposure to PM emitted during 3D printing (Figure 1A). 5 μg/mL and 10 μg/mL PM were used as the administered concentrations to cover the range of extrapolated *in vitro* doses described in Table 1 and to identify the biologically effective dose. Cells exposed to extracts from blank filters did not display decreased viability (data not shown). In comparison to untreated cells, cells exposed to control room PM did not experience significant reductions in cellular viability (Figure 1A). Exposure to 10 μg/mL PM emitted during printing with ABS significantly reduced cellular viability to 51%, while exposure to 5 μg/mL had a slight but non-significant effect (74.1% viability). Exposure to both doses of PM emitted during printing with PLA also significantly reduced cell viability. Specifically, cells exposed to 5 μg/mL and 10 μg/mL PLA were 44 and 59% viable, respectively. We also confirmed that SAEC lysates contained minimal levels of endotoxin. These levels were not significantly different from levels in negative control cells and are below the available FDA limits for sterile water and medical device eluates (0.25 and 0.5 EU/mL, respectively). Therefore, bacterial contamination had a minimal effect on the toxicological endpoints measured (Supplementary Figure S2).

### Genotoxicity of 3D printer emissions

To explore the potential genotoxicity of ABS and PLA emissions, we measured a panel of seven DNA damage and repair pathway proteins in SAEC after 24 h of exposure to 5 μg/mL and 10 μg/mL doses of PM emitted during 3D printing (Figure 1B). Although most proteins did not change significantly in response to ABS or PLA emissions at either dose, murine double minute clone 2 (MDM2) increased upon exposure to ABS and PLA-emitted PM at 10 μg/mL. Additionally, gamma-H2AX increased in response to 10 μg/mL PM collected during PLA printing.

### Effect of 3D printer emissions on release of pro-inflammatory factors

To determine the effect of exposure to PM emitted during 3D printing on inflammation, we measured a panel of 20 cytokines, chemokines, and other pro-inflammatory factors in SAEC supernatants. Exposure to ABS and PLA emissions increased the release of some pro-inflammatory cytokines relative to untreated cells, although these increases did not reach statistical significance. Specifically, exposure to PM emitted during printing with PLA increased IL-1β at both doses (Figure 1C). Exposure to PM emitted during printing with ABS and PLA at both doses increased MMP-9 release. Finally, exposure to ABS and PLA at 5 μg/mL elevated RANTES.

### Effect of 3D printer emissions on the metabolome

**Figure 2:**
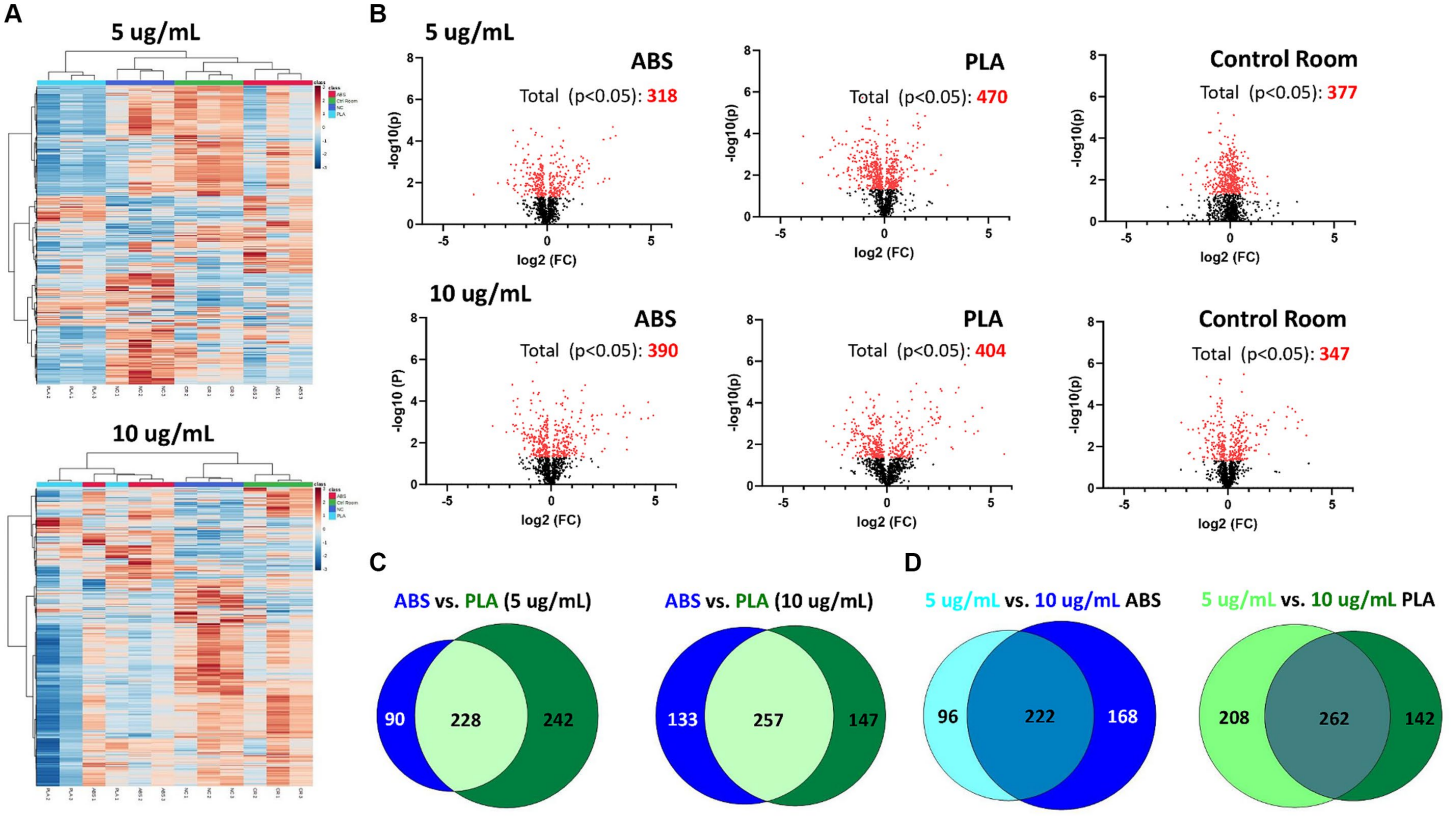
Metabolic changes in SAEC exposed to low (5 μg/mL) and high (10 μg/mL) doses of PM collected during 3D printing. **(A)** Heatmaps depict HCA of all metabolic features detected in cells exposed to ABS emissions (red), PLA emissions (light blue), control room PM (green), and untreated (NC) cells (dark blue). **(B)** Volcano plots depict the significantly altered metabolites for each exposure (*p* < 0.05 relative to NC). Venn diagrams compare the significantly altered metabolites (*p* < 0.05 relative to NC) between **(C)** each filament for a given dose and **(D)** between doses for a given filament.

Using HPLC MS/MS, we characterized the metabolites released by SAEC exposed to ABS, PLA, and control classroom emissions, alongside untreated negative control (NC) cells (Figure 2). Hierarchical clustering analysis (HCA) of all detected metabolites yielded separate clusters for NC, cells exposed to control classroom PM, and cells exposed to 3D printer emissions (Figure 2A). This was the case for both low (5 μg/mL) and high (10 μg/mL) exposures. This suggests that 3D printer emissions and ambient classroom air both have distinct effects on cellular metabolic profiles. Notably, in the high dose exposure, ABS and PLA-exposed cells were not clustered separately from one another, suggesting that at higher doses, printer filament types may differ less in terms of their effects on cellular metabolism.

To further examine the metabolomic responses revealed by the HCA, we identified metabolites that were significantly altered relative to NC cells at each dose for each treatment group (Figure 2B; Supplementary Tables S1–S6). SAEC exposed to PLA emissions yielded the highest number of significantly altered metabolites at both the low (*n* = 470) and high (*n* = 404) dose (Figure 2B). For the low dose exposure group, this was followed by cells exposed to the control room emissions (*n* = 377) and then ABS emissions (*n* = 318). For the high dose exposure group, this was followed by cells exposed to ABS emissions (*n* = 390) and then control room emissions (*n* = 347).

We also compared the overlap in significantly altered metabolites across filaments and doses (Figures 2C,D). Although ABS and PLA-exposed cells shared 228 and 257 significantly altered metabolites at low and high doses respectively, we still identified metabolites that were uniquely altered for each filament type (Figure 2C). Additionally, 222 metabolites were shared between low and high doses for ABS, and 262 metabolites were shared between low and high doses for PLA (Figure 2D).

### Pathway analysis of metabolic changes

**Figure 3:**
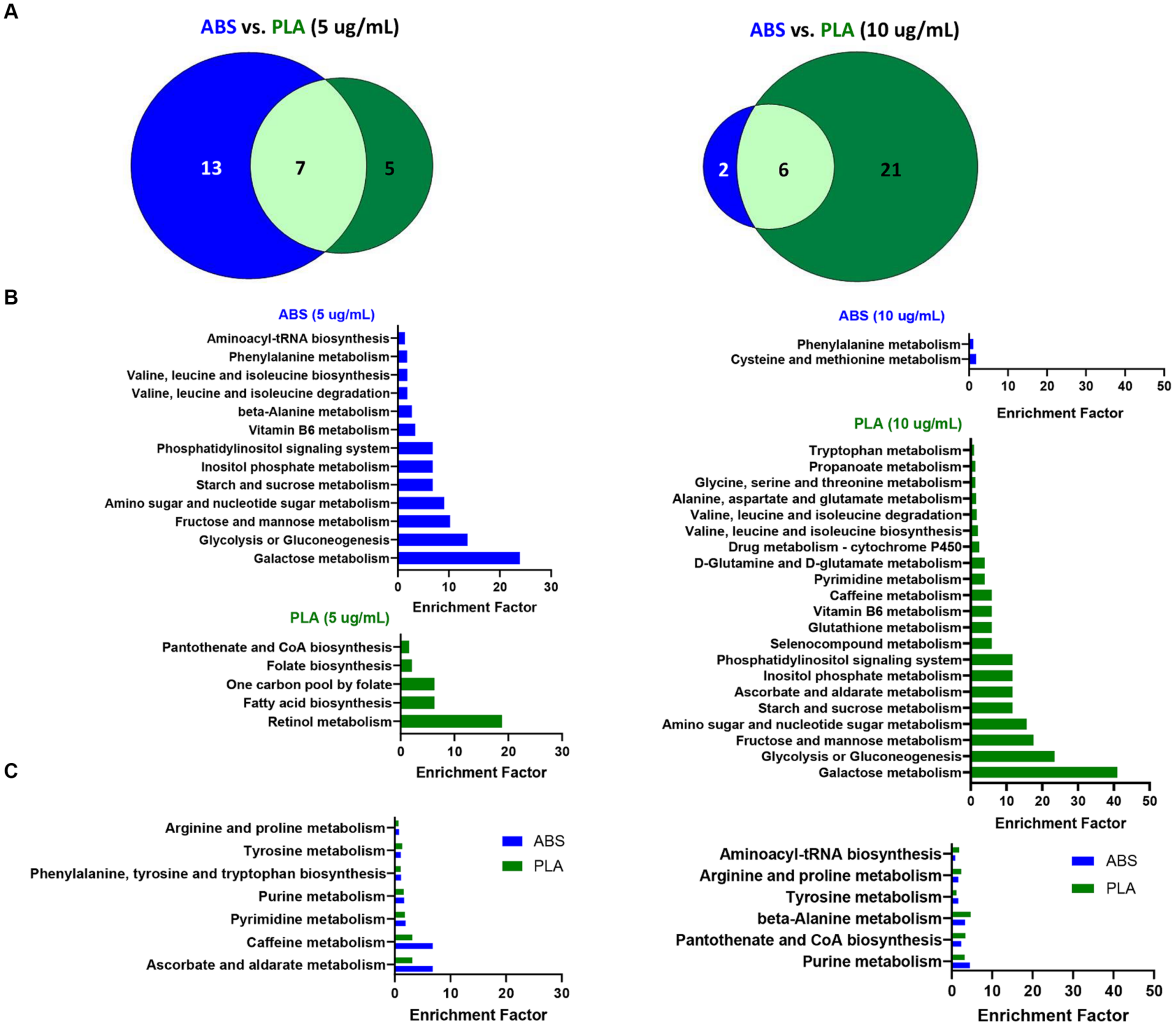
Metabolic pathways altered by ABS vs. PLA emissions at each dose. **(A)** Venn diagrams depict the overlap between significantly enriched pathways (*p* < 0.05 relative to NC cells) in ABS and PLA-exposed cells. Histograms list the significantly enriched pathways (*p* < 0.05 relative to NC cells) that were **(B)** unique to ABS or PLA and **(C)** shared between ABS and PLA.

To determine the biological significance of the metabolic changes noted above, we used the MS Peaks to Pathways module on Metaboanalyst to identify metabolic pathways that were significantly enriched in SAECs exposed to ABS emissions and PLA emissions relative to untreated negative control (NC) cells (Supplementary Tables S7–S12). Next, we compared the overlap in significantly enriched pathways between ABS and PLA filaments at each tested dose (5 μg/mL and 10 μg/mL, respectively) (Figure 3). Although ABS and PLA-exposed cells shared some significantly enriched pathways at each dose (Figures 3A,C), we also identified pathways that were uniquely enriched for each filament type (Figure 3B). At the low dose, ABS-enriched pathways were primarily related to carbohydrate metabolism, whereas PLA-enriched pathways were related to metabolism of cofactors and vitamins and amino acid metabolism (Figure 3B; Supplementary Tables S8, S9). Conversely, for the high dose exposure, ABS-enriched pathways were primarily related to amino acid metabolism, whereas PLA-enriched pathways were primarily related to carbohydrate metabolism (Figure 3B; Supplementary Tables S11, S12). These results support our differential metabolomics data by suggesting that ABS and PLA have distinct effects on cellular metabolism, this time at the pathway level.

**Figure 4:**
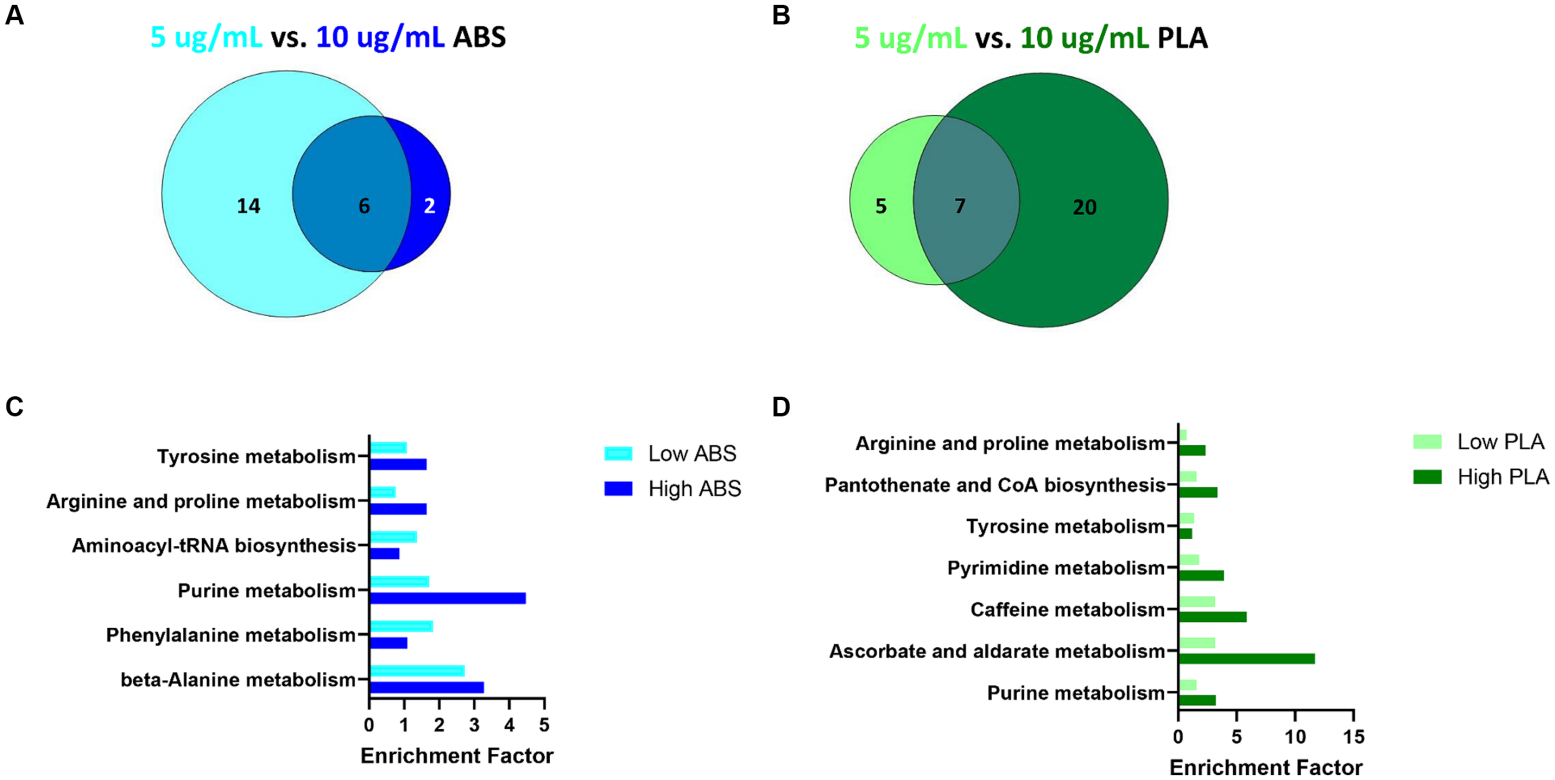
Metabolic pathways altered by low (5 μg/mL) vs. high (10 μg/mL) doses for a given filament. Venn diagrams depict the overlap between significantly enriched pathways (*p* < 0.05 relative to NC cells) in **(A)** ABS-exposed cells at each dose and **(B)** PLA-exposed cells at each dose. Histograms depict the enrichment factor for significantly enriched pathways (*p* < 0.05 relative to NC cells) that were shared between high and low doses for **(C)** ABS-exposed cells and **(D)** PLA-exposed cells.

To distinguish the effect of dose on metabolic pathway enrichment, we compared the pathways that were significantly enriched for both low and high doses of a given filament (Figure 4). Specifically, six pathways were shared between both doses for ABS (Figures 4A,C) and seven pathways were shared between both doses for PLA (Figures 4B,D), suggesting that the effects of a given 3D printer filament exposure on metabolic pathways vary greatly depending on the dose.

## Discussion

In this study, we characterized and sampled particulates emitted from ABS and PLA filaments during 3 h 3D printer operation at a high school. We also exposed small airway epithelial cells (SAEC) to the sampled emissions and studied effects on cell viability, DNA damage, inflammation, and cellular metabolomics after 24 h of exposure. By evaluating a single classroom exposure and comparing across different doses, this field investigation builds on previous metabolomics studies conducted in laboratory settings (20). In doing so, it reveals important insights into the potential respiratory consequences of 3D printing exposure and the metabolic changes that govern these effects.

Our toxicological data suggests that ABS and PLA-emitted PM are potentially cytotoxic and genotoxic. Specifically, exposure to emissions from both filament types reduced airway epithelial cell viability, which was previously observed in laboratory studies (16, 17, 20, 21). In addition to impacting cell viability, exposure to PM emitted from both ABS and PLA filaments increased levels of murine double minute clone 2 (MDM2), which is observed in different types of cancers and promotes genomic instability (22). Specifically, increased MDM2 can negatively regulate p53 in order to reduce DNA repair activity. Although p53 did not decrease in the present study, MDM2 can also function independently of p53 to inhibit DNA breakage repair through associating with the Mre11/Rad50/Nbs1 DNA repair complex (22). Future studies should explore the impact of ABS and PLA- emitted PM on these different mechanisms of MDM2-mediated genomic instability.

Additionally, we found that exposure to PM emitted during 3D printing with PLA filaments, but not ABS filaments, induced DNA damage in SAEC as measured by increased gamma-H2AX. Formation of gamma-H2AX occurs upon phosphorylation of the Ser-139 residue of the histone variant H2AX and is an early response to DNA double-strand breakage that recruits DNA repair proteins (23). Given that DNA damage and reduced DNA repair capacity are both involved in asthma development (24, 25), these findings reveal potential mechanisms that mediate the development of asthma-like symptoms in 3D printer users (26, 27).

In support of our toxicological data, several metabolic pathways that function during DNA damage and repair were perturbed in cells exposed to ABS and PLA-emitted PM. Specifically, purine and pyrimidine metabolism, amino sugar and nucleotide sugar metabolism, and intermediates from glucose, glutamine, and aspartate metabolisms were perturbed by 3D printer filament exposure in the present study. Importantly, these metabolic pathways function to regulate the pool of nucleotides available for DNA repair (28). Additionally, ABS and PLA-emitted PM disrupted cysteine metabolism and glutathione metabolism, respectively, at the high dose, suggesting that 3D printer emissions may additionally induce genotoxicity through de-regulating redox homeostasis (28). Taken together, these results provide additional support for the potential genotoxicity of ABS and PLA-emitted PM at the level of cellular metabolism.

Additionally, exposure to 3D printer-emitted PM altered several metabolites and pathways that are implicated in respiratory disease. Specifically analysis of the serum, plasma, blood, urine, and exhaled breath condensate of asthma patients have observed dysregulated tyrosine, arginine, purine, and phenylalanine metabolites (29, 30). Arginine and phenylalanine are also dysregulated in COPD patients (31). According to an additional study, arginine expression was elevated in the plasma of patients with chronic obstructive pulmonary disease (COPD) compared to the healthy population and was further elevated in acute exacerbation of COPD (AECOPD) (32). Furthermore, pathway analysis of altered metabolites in serum samples collected from patients with allergic rhinitis revealed that purine metabolism was enriched relative to healthy controls and that the associated metabolites hypoxanthine and urate could be potential biomarkers (33). Therefore, the metabolic changes noted in this study provide early indicators that 3D printer-emitted PM may induce adverse respiratory consequences.

Moreover, several pathways that were altered by 3D printer-emitted PM in this study were also associated with PM exposure from ambient air pollution, indoor classroom air, and occupational exposures in other studies. Specifically, in patients with silicosis, arginine and proline metabolism was the major perturbed pathway relative to healthy controls (34). Furthermore, the abundance of L-arginine was negatively correlated with the predicted percentage of forced vital capacity in these patients, which is a measure of lung function. In children exposed to ambient classroom air, both PM_0.5_ exposure and decreased pulmonary function were associated with dysregulated purine metabolism (35). In plasma from patients with COPD, arginine and proline metabolism was affected by PM_2.5_ exposure, and arginine was positively associated with acute exacerbation of COPD (AECOPD). Taken together, these findings suggest that perturbed amino acid metabolism and purine metabolism may play a role in the adverse respiratory effects associated with 3D printer emissions exposures.

We previously found that exposure to ABS and PLA-emitted PM triggered formation of reactive oxygen species (ROS), increased total glutathione, and release of pro-inflammatory cytokines in airway epithelial cells (4, 20). Although we did not measure oxidative stress in the present study, ABS and PLA emissions exposures perturbed metabolic pathways that have previously been observed alongside PM-induced oxidative stress and inflammation, which are key events responsible for the increased risk of COPD, asthma, and other lung diseases associated with PM exposure (36, 37). Specifically, tyrosine metabolism was enriched in blood from healthy volunteers 2 h following exposure to ambient air pollution. In the same cohort, tyrosine levels correlated with fibrinogen levels, which increase in the presence of inflammation (38). Altered purine metabolism was observed alongside disrupted pro-oxidant/antioxidant balance in rodents exposed to PM_2.5_ via intratracheal instillation (39). Purine metabolism was also altered in mice with PM_2.5_ exposure-induced asthma. Furthermore, five inflammatory cytokines (IL-4, IL-5, IL-13, IL-1β, IL-8) were positively correlated with levels of uric acid, which is a product of purine metabolism (40). Additionally, exposure to PM_2.5_ perturbed purine metabolism and arginine and proline metabolism in BEAS-2B cells. This was coupled with significant increases in oxidative stress markers including reactive oxygen species (ROS), malondialdehyde (MDA), and nitric oxide (NO) pro-inflammatory cytokines (TNF-α, IL-6 and IL-1β), and metabolic reprogramming from oxidative phosphorylation to glycolysis (41). Furthermore, in two studies, purine metabolism and pyrimidine metabolism were enriched alongside increased alongside increases in gamma-H2AX in rats exposed to long-term low-level PM_2.5_ and O_3_ through ambient air pollution. Results also showed that the DNA damage biomarker gamma-H2AX in the lungs was positively correlated with ADP and N-acetyl-D-glucosamine, which are two serum metabolites involved in these pathways (42, 43). Given that 3D printers emit fine and ultrafine particles, the above studies reveal how the metabolic changes noted in this study may be mechanistically linked to respiratory symptoms reported by occupational users of 3D printers.

Our data additionally suggest that the toxicological and metabolic effects of 3D printer emissions differ depending on the filament used, with PLA impacting more of the endpoints studied. For example, although both ABS and PLA-emitted PM impacted cell viability, MDM2, and increased MMP-9, only PLA increased gamma-H2AX, IL-1β and RANTES. The metabolomic alterations observed in this study additionally support this. Specifically, PLA exposure resulted in larger numbers of significantly altered metabolites relative to ABS at both tested doses. Pathway analysis further revealed that ABS primarily altered pathways related to carbohydrate metabolism at the low dose and amino acid metabolism at the high dose. Conversely, PLA exposure altered pathways related to cofactor and vitamin metabolism at the low dose and carbohydrate metabolism at the high dose. Taken together, these results agree with our previous finding that ABS and PLA exposure perturbed different metabolic pathways (20).

A combination of physical and chemical properties of 3D printer-emitted particles was likely responsible for the filament-specific differences in cellular outcomes. PLA-emitted PM potentially produced more toxicological effects and altered more metabolites in the present study because of particle kinetics that caused a larger effective dose relative to ABS-emitted particles. Specifically, our aerosol characterization data predicted higher deposition of PLA-emitted PM relative to ABS, which was likely due to the larger size of PLA-emitted particles. In addition to size, differences in the effective density of ABS and PLA-emitted PM may have increased the effective dose of PLA-emitted PM relative to ABS when particles were dispersed in cell culture media. Specifically, particles with effective densities lower than the cell culture media will exhibit buoyancy, which may alter particle deposition and dose–response relationship (44). To support this, the raw material density of PLA is higher than the density of cell culture media (1.25 g/cm^3^ vs. ~1.0 g/cm^3^) (44–46). Conversely, ABS PM may float or settle at a lower rate in cell culture media due to a similar density (1.05 g/cm^3^) (47), which may have contributed to certain differences found in genotoxicity and metabolic profiling. Therefore, future studies should measure the physicochemical properties of 3D printer emissions including effective density alongside markers of genotoxicity to distinguish which filament and PM properties produce these effects.

It is important to note that this study is not without limitations. First, we were unable to control for other sources of particulate emissions in the printer room that could have resulted from classroom activities. Second, due to scheduled classroom activities, we were only allowed to sample and collect PM on restricted occasions for each experimental group. Future studies should consider more sampling occasions and duplicates to better monitor the environments. Third, we did not characterize the chemical composition of 3D printer emissions. Although we previously characterized VOCs and metals present in ABS and PLA 3D printer emissions (4, 20), future studies should measure physical and chemical properties of emissions alongside toxicological outcomes to distinguish their contributions to toxicity. Fourth, as discussed above, SAEC were exposed to PM in a submerged format, which may have altered the particle kinetics and cellular uptake of PM despite equivalent administered doses. Notably, we addressed this limitation, as well as the potential for external sources of PM in ambient air, in a previously published laboratory study by measuring metabolomic responses in cells cultured in air-liquid interface in real-time during 3D printing (20). Furthermore, SAEC were exposed to PM after sampling and extraction from filters, which raises the possibility of particle alterations due to the solvent extraction method employed. As the present study is a field study conducted in a non-laboratory setting, this limitation was unavoidable. Finally, because particulates were sampled outside of a laboratory setting, SAEC may have been subjected to bacterial contamination. To address this, we confirmed that the endotoxin levels in cells exposed to PM were below FDA limits and were not significantly different from the levels detected in untreated negative control cells. Therefore, the toxicological and metabolic changes observed in this study were likely due to PM exposure rather than due to bacterial contamination.

## Conclusion

The data presented here suggest that after a single printing job in a high school classroom, 3D-printers emit fine and ultrafine particles, which may compromise cellular viability and induce genotoxic effects in airway epithelial cells. Furthermore, to determine molecular mechanisms governing these effects, we measured metabolic responses to 3D printer emissions exposure in SAEC, which varied depending on the filament used. Although SAEC metabolic responses also varied depending on the dose, we identified metabolic pathways that were enriched across both doses for a given filament. Importantly, some of these pathways play known roles in oxidative stress, DNA damage, and inflammation induced by PM exposures and are implicated in respiratory diseases such as asthma, allergic rhinitis, and COPD. Taken together, these results reveal early molecular events that may drive 3D printer-induced respiratory toxicity.

## Data availability statement

The original contributions presented in the study are included in the article/Supplementary material, further inquiries can be directed to the corresponding author.

## Ethics statement

Ethical approval was not required for the studies on humans in accordance with the local legislation and institutional requirements because only commercially available established cell lines were used. Ethical approval was not required for the studies on animals in accordance with the local legislation and institutional requirements because only commercially available established cell lines were used.

## Author contributions

LB: Formal analysis, Investigation, Visualization, Writing – original draft, Writing – review & editing. QZ: Formal analysis, Investigation, Writing – review & editing. SS: Investigation, Visualization, Writing – review & editing. SA: Formal analysis, Investigation, Writing – review & editing. JS: Writing – review & editing, Conceptualization, Formal analysis, Investigation. MB: Writing – review & editing. CW: Conceptualization, Investigation, Supervision, Writing – review & editing.

## Footnotes

^1^ http://prime.psc.riken.jp/compms/msdial/main.html

^2^ http://prime.psc.riken.jp/compms/msdial/main.html#MSP

^3^ https://www.metaboanalyst.ca/

^4^ https://github.com/PNNL-Comp-Mass-Spec/Venn-Diagram-Plotter/releases
