# Supplementary material for: 3D printer emissions elicit filament-specific and dose-dependent metabolic and genotoxic effects in human airway epithelial cells
Source: Front Public Health. 2024 Jul 12;12:1408842. doi: 10.3389/fpubh.2024.1408842 (PMC11273288; doi:10.3389/fpubh.2024.1408842)
Supplement: Supplementary file 1 [file Data_Sheet_1.docx]

**Supplementary Material**

**Supplementary Figures**

**Figure S1.** Averaged particle number size distribution for printing with ABS and PLA materials. CRTL= control classroom without 3D printers; BG= background before the printer started to operate; PRINT= during printing duration. Averaged total particle number concentrations are shown in the annotation for each sampling scenario.

**Figure S2.** Endotoxin levels in lysates from small airway epithelial cells (SAEC) exposed to particulate matter (PM) collected from the control room, ABS printing room, and PLA printing room at low (5 µg/mL) and high (10 µg/mL) doses compared to FDA-approved limits. Error bars represent ± 1 standard deviation from the mean (*n*=2). NC= untreated negative control cells.

**Supplementary Tables**

**Table S1.** Significantly Altered Metabolites in SAECs Exposed to 5 µg/mL PM from the Control Room.

| m/z | rt | Metabolite Name | p.value | FDR | log2(FC) |
| --- | --- | --- | --- | --- | --- |
| 708.511 | 22.725 | PS(O-16:0/15:0) | 0.007 | 0.046 | 1.797 |
| 149.061 | 4.298 | Unidentified | 0.048 | 0.129 | 1.303 |
| 105.071 | 19.736 | L-2,3-DIAMINOPROPIONIC ACID | 0.023 | 0.081 | 1.277 |
| 163.089 | 4.132 | Unidentified | 0.045 | 0.124 | 1.199 |
| 736.214 | 4.295 | Unidentified | 0.045 | 0.124 | 1.198 |
| 366.113 | 2.676 | Unidentified | 0.014 | 0.061 | 1.188 |
| 258.066 | 5.776 | Unidentified | 0.020 | 0.075 | 1.137 |
| 268.136 | 11.140 | Unidentified | 0.008 | 0.049 | 1.102 |
| 463.190 | 5.771 | Ethyl 16-hydroxy-18-bromo-8E,17E19Z-tricosatrien-4,6-diynoate | 0.016 | 0.069 | 1.087 |
| 736.536 | 22.760 | PS(O-18:0/15:0) | 0.013 | 0.060 | 1.058 |
| 235.975 | 6.961 | Unidentified | 0.039 | 0.111 | 0.967 |
| 217.065 | 16.239 | Unidentified | 0.004 | 0.036 | 0.941 |
| 323.161 | 6.721 | Nordurlettone | 0.009 | 0.050 | 0.867 |
| 324.106 | 1.310 | Unidentified | 0.001 | 0.021 | 0.860 |
| 147.046 | 2.223 | Coumaric acid | 0.018 | 0.072 | 0.844 |
| 153.024 | 7.529 | Unidentified | 0.014 | 0.064 | 0.789 |
| 137.047 | 1.820 | Hypoxanthine | 0.003 | 0.031 | 0.754 |
| 634.741 | 16.356 | Cer(d18:2/23:0) | 0.046 | 0.125 | 0.743 |
| 236.112 | 2.569 | N-(9-oxodecyl) acetamide | 0.000 | 0.021 | 0.702 |
| 160.043 | 14.016 | Unidentified | 0.018 | 0.072 | 0.701 |
| 113.034 | 2.589 | Uracil | 0.001 | 0.021 | 0.622 |
| 110.036 | 2.300 | Hypotaurine | 0.013 | 0.061 | 0.621 |
| 482.401 | 22.062 | PC(O-15:0/O-1:0) [U] | 0.009 | 0.050 | 0.603 |
| 216.124 | 1.399 | Unidentified | 0.011 | 0.055 | 0.584 |
| 414.218 | 20.064 | Unidentified | 0.031 | 0.098 | 0.576 |
| 241.100 | 7.442 | Unidentified | 0.009 | 0.050 | 0.567 |
| 212.002 | 1.154 | Se-Methylselenomethionine | 0.024 | 0.083 | 0.567 |
| 197.112 | 7.449 | Unidentified | 0.011 | 0.056 | 0.552 |
| 423.194 | 1.972 | Unidentified | 0.000 | 0.021 | 0.535 |
| 238.071 | 2.638 | Unidentified | 0.003 | 0.033 | 0.493 |
| 367.152 | 6.750 | Unidentified | 0.002 | 0.025 | 0.491 |
| 524.077 | 1.377 | Unidentified | 0.030 | 0.097 | 0.486 |
| 414.359 | 21.464 | Heptadecanoyl carnitine | 0.011 | 0.056 | 0.474 |
| 320.135 | 6.755 | Unidentified | 0.003 | 0.033 | 0.473 |
| 598.340 | 18.588 | Unidentified | 0.023 | 0.082 | 0.472 |
| 642.519 | 22.062 | GlcCer(d14:2(4E,6E)/16:0) | 0.002 | 0.028 | 0.467 |
| 424.162 | 16.360 | Unidentified | 0.044 | 0.122 | 0.460 |
| 611.263 | 3.300 | Unidentified | 0.043 | 0.120 | 0.455 |
| 326.119 | 7.458 | Citalopram (propionic acid derivative) | 0.008 | 0.048 | 0.437 |
| 70.074 | 1.961 | 1-Pyrroline | 0.043 | 0.121 | 0.425 |
| 432.238 | 19.741 | 17-phenoxy trinor PGF2α ethyl amide | 0.022 | 0.080 | 0.420 |
| 303.068 | 5.293 | Unidentified | 0.046 | 0.125 | 0.420 |
| 453.339 | 14.548 | (17E)-1α,25-dihydroxy-26,27-dimethyl-17,20,22,22,23,23-hexadehydro-24a-homovitamin D3 / (17E)-1α,25-dihydroxy-26,27-dimethyl-17,20,22,22,23,23-hexadehydro-24a-homocholecalciferol | 0.037 | 0.110 | 0.420 |
| 549.343 | 1.992 | 5beta-scymnol sulfate | 0.035 | 0.106 | 0.410 |
| 310.224 | 5.016 | Unidentified | 0.004 | 0.034 | 0.406 |
| 514.325 | 18.595 | Unidentified | 0.009 | 0.050 | 0.392 |
| 148.132 | 0.975 | Unidentified | 0.028 | 0.093 | 0.373 |
| 310.315 | 5.011 | Unidentified | 0.017 | 0.070 | 0.372 |
| 258.094 | 2.561 | Unidentified | 0.002 | 0.025 | 0.370 |
| 438.304 | 19.409 | PE(P-16:0/0:0) | 0.001 | 0.021 | 0.365 |
| 305.148 | 6.735 | Unidentified | 0.009 | 0.050 | 0.364 |
| 162.091 | 5.016 | 1-(2,3-Dihydro-1H-pyrrolizin-5-yl)-2-propen-1-one | 0.008 | 0.047 | 0.359 |
| 241.078 | 1.632 | Unidentified | 0.003 | 0.033 | 0.358 |
| 290.126 | 3.565 | Unidentified | 0.008 | 0.047 | 0.357 |
| 401.103 | 2.205 | S-Acetylphosphopantetheine | 0.013 | 0.060 | 0.353 |
| 152.055 | 1.279 | Guanine | 0.002 | 0.026 | 0.348 |
| 473.177 | 2.162 | C1'-C9-Glycosylated UWM6 | 0.001 | 0.021 | 0.346 |
| 514.410 | 21.803 | Sulfolithocholylglycine | 0.003 | 0.032 | 0.341 |
| 331.114 | 0.803 | Unidentified | 0.010 | 0.053 | 0.318 |
| 294.416 | 3.258 | Unidentified | 0.024 | 0.085 | 0.317 |
| 366.094 | 5.041 | 2-S-Glutathionyl acetate | 0.008 | 0.047 | 0.317 |
| 349.136 | 6.750 | Unidentified | 0.003 | 0.031 | 0.305 |
| 100.114 | 4.884 | cyclohexylammonium | 0.023 | 0.081 | 0.302 |
| 220.045 | 16.248 | 4-Amino-2-methyl-5-phosphomethylpyrimidine | 0.010 | 0.052 | 0.299 |
| 679.234 | 5.035 | Unidentified | 0.000 | 0.021 | 0.296 |
| 170.120 | 3.229 | Unidentified | 0.019 | 0.073 | 0.293 |
| 431.262 | 6.548 | 17-phenyl trinor PGF2α isopropyl ester | 0.017 | 0.070 | 0.293 |
| 98.962 | 19.790 | Phosphoric acid | 0.017 | 0.071 | 0.292 |
| 275.162 | 1.070 | Unidentified | 0.050 | 0.133 | 0.282 |
| 608.386 | 16.452 | Unidentified | 0.014 | 0.063 | 0.281 |
| 310.126 | 5.021 | Unidentified | 0.002 | 0.027 | 0.277 |
| 384.299 | 18.639 | Unidentified | 0.009 | 0.050 | 0.276 |
| 319.035 | 5.778 | Unidentified | 0.006 | 0.045 | 0.269 |
| 417.073 | 2.210 | Unidentified | 0.033 | 0.102 | 0.268 |
| 496.474 | 21.797 | PS (16:0/0:0) | 0.033 | 0.102 | 0.259 |
| 311.229 | 5.014 | 9-hydroperoxy-10E,12,15Z-octadecatrienoic acid | 0.006 | 0.043 | 0.258 |
| 276.142 | 3.271 | Tryptophyl-Alanine | 0.001 | 0.024 | 0.248 |
| 518.237 | 1.366 | PS (18:4(6Z,9Z,12Z,15Z)/0:0) | 0.035 | 0.104 | 0.244 |
| 247.128 | 4.380 | L-beta-aspartyl-L-leucine | 0.010 | 0.052 | 0.241 |
| 276.211 | 3.270 | Unidentified | 0.001 | 0.024 | 0.240 |
| 143.056 | 1.404 | Unidentified | 0.025 | 0.087 | 0.239 |
| 177.991 | 1.205 | Unidentified | 0.031 | 0.098 | 0.238 |
| 119.075 | 4.299 | Methyl-3-hydroxybutyric acid | 0.004 | 0.036 | 0.238 |
| 248.095 | 1.793 | Glutamyl-Threonine | 0.028 | 0.093 | 0.237 |
| 292.019 | 1.181 | N-Succinyl-L-diaminopimelic acid | 0.010 | 0.053 | 0.237 |
| 162.078 | 6.751 | L-2-Aminoadipic acid | 0.004 | 0.037 | 0.235 |
| 293.172 | 1.067 | (E)-2-decylpent-2-enedioic acid | 0.003 | 0.034 | 0.231 |
| 376.317 | 20.069 | N-(1,1-dimethyl-2-hydroxy-ethyl) arachidonoyl amine | 0.025 | 0.087 | 0.231 |
| 102.056 | 1.715 | Unidentified | 0.010 | 0.052 | 0.226 |
| 340.089 | 4.301 | 6-Hydroxy-5-methoxyindole glucuronide | 0.044 | 0.123 | 0.223 |
| 781.252 | 0.776 | N, N'-Diacetylchitobiosyldiphosphodolichol | 0.002 | 0.028 | 0.223 |
| 348.123 | 20.069 | Unidentified | 0.009 | 0.050 | 0.222 |
| 114.046 | 6.538 | Unidentified | 0.013 | 0.061 | 0.221 |
| 194.023 | 1.263 | Unidentified | 0.001 | 0.021 | 0.221 |
| 582.174 | 2.205 | Unidentified | 0.012 | 0.060 | 0.220 |
| 605.175 | 1.935 | SP1 | 0.006 | 0.043 | 0.217 |
| 308.114 | 2.737 | Cysteinyl-Tryptophan | 0.007 | 0.046 | 0.215 |
| 182.210 | 2.216 | Unidentified | 0.000 | 0.011 | 0.213 |
| 178.088 | 5.011 | O-Ureidohomoserine | 0.014 | 0.062 | 0.211 |
| 309.226 | 17.938 | Unidentified | 0.022 | 0.078 | 0.209 |
| 367.318 | 6.744 | Unidentified | 0.006 | 0.044 | 0.209 |
| 298.129 | 2.722 | Phenethylamine glucuronide | 0.007 | 0.046 | 0.207 |
| 276.120 | 6.746 | Norophthalmic acid | 0.000 | 0.021 | 0.207 |
| 140.107 | 3.271 | Unidentified | 0.004 | 0.036 | 0.204 |
| 328.139 | 5.014 | PC(O-2:0/2:0) | 0.002 | 0.030 | 0.202 |
| 208.099 | 5.024 | N-Acetyl-D-phenylalanine | 0.003 | 0.033 | 0.200 |
| 220.081 | 6.735 | O-Succinyl-L-homoserine | 0.000 | 0.020 | 0.199 |
| 281.210 | 1.404 | 12S-hydroxy-5Z,8E,10E-heptadecatrienoic acid | 0.022 | 0.079 | 0.197 |
| 350.067 | 1.805 | N-(5-Phospho-D-ribosyl) anthranilate | 0.008 | 0.047 | 0.191 |
| 208.143 | 20.067 | Unidentified | 0.038 | 0.110 | 0.190 |
| 105.071 | 5.021 | Unidentified | 0.001 | 0.021 | 0.187 |
| 204.123 | 12.327 | Acetylcarnitine | 0.016 | 0.069 | 0.180 |
| 161.068 | 1.405 | Unidentified | 0.005 | 0.042 | 0.179 |
| 516.224 | 1.070 | Unidentified | 0.023 | 0.082 | 0.178 |
| 363.157 | 2.202 | Unidentified | 0.008 | 0.048 | 0.178 |
| 132.082 | 5.010 | Unidentified | 0.001 | 0.021 | 0.171 |
| 107.047 | 5.018 | Unidentified | 0.004 | 0.036 | 0.168 |
| 540.216 | 1.242 | Unidentified | 0.007 | 0.046 | 0.167 |
| 228.085 | 4.069 | L-Arogenate | 0.000 | 0.004 | 0.167 |
| 166.086 | 14.016 | L-Phenylalanine | 0.020 | 0.075 | 0.165 |
| 133.030 | 1.785 | Unidentified | 0.040 | 0.114 | 0.163 |
| 116.071 | 1.672 | L-Proline | 0.010 | 0.053 | 0.163 |
| 693.216 | 5.019 | Unidentified | 0.001 | 0.021 | 0.154 |
| 132.102 | 2.014 | L-Isoleucine | 0.002 | 0.028 | 0.153 |
| 256.081 | 0.791 | methyl 2-[(2,3-dihydroxybenzoyl) amino]-3-hydroxypropanoate | 0.003 | 0.033 | 0.152 |
| 130.064 | 15.207 | 3-Methylene-indolenine | 0.003 | 0.033 | 0.151 |
| 357.160 | 1.664 | xi-3-Hydroxy-5-phenylpentanoic acid O-beta-D-Glucopyranoside | 0.043 | 0.121 | 0.148 |
| 418.252 | 1.990 | 16-phenoxy Prostaglandin F2a ethyl amide | 0.044 | 0.122 | 0.147 |
| 79.022 | 1.135 | Unidentified | 0.001 | 0.024 | 0.138 |
| 504.176 | 1.260 | TyrMe-Trp-OH | 0.000 | 0.013 | 0.137 |
| 370.199 | 2.011 | PC (7:0/0:0) | 0.019 | 0.073 | 0.135 |
| 248.150 | 3.232 | Malonylcarnitine | 0.004 | 0.036 | 0.131 |
| 128.107 | 3.259 | N-Cyclohexylformamide | 0.019 | 0.073 | 0.130 |
| 114.091 | 7.095 | Unidentified | 0.001 | 0.021 | 0.129 |
| 354.147 | 11.344 | Unidentified | 0.034 | 0.102 | 0.126 |
| 285.090 | 8.626 | Cysteinyl-Tyrosine | 0.030 | 0.096 | 0.123 |
| 294.156 | 3.258 | Unidentified | 0.011 | 0.056 | 0.123 |
| 172.042 | 1.275 | Unidentified | 0.000 | 0.017 | 0.122 |
| 176.065 | 1.979 | Unidentified | 0.018 | 0.072 | 0.118 |
| 201.956 | 1.564 | Selenocysteine seleninic acid | 0.007 | 0.046 | 0.117 |
| 163.110 | 14.364 | 3-hydroxy-3-methyl-Glutaric acid | 0.005 | 0.038 | 0.112 |
| 321.144 | 6.739 | Unidentified | 0.001 | 0.021 | 0.110 |
| 367.225 | 6.741 | 1,2-dihydroxyheptadec-16-en-4-yl acetate | 0.005 | 0.038 | 0.109 |
| 455.166 | 0.981 | Phe-Phe4Cl-OH | 0.003 | 0.031 | 0.108 |
| 354.075 | 4.780 | Unidentified | 0.030 | 0.097 | 0.106 |
| 273.089 | 18.650 | Unidentified | 0.003 | 0.033 | 0.106 |
| 385.316 | 11.494 | N-palmitoyl glutamine | 0.034 | 0.104 | 0.105 |
| 369.075 | 19.950 | 2-Deoxy-4-O-[(2E)-3-(4-hydroxyphenyl)-2-propenoyl]-3-C-(methoxycarbonyl)pentaric acid | 0.002 | 0.026 | 0.102 |
| 102.047 | 4.300 | 1-Aminocyclopropane-1-carboxylic acid | 0.011 | 0.056 | 0.100 |
| 210.044 | 7.521 | Unidentified | 0.013 | 0.061 | 0.100 |
| 528.120 | 14.018 | Unidentified | 0.007 | 0.046 | 0.094 |
| 300.103 | 1.069 | 8-hydroxy Guanosine | 0.009 | 0.050 | 0.092 |
| 161.960 | 1.211 | Unidentified | 0.012 | 0.060 | 0.091 |
| 230.137 | 3.246 | Unidentified | 0.002 | 0.026 | 0.091 |
| 91.055 | 5.019 | Unidentified | 0.001 | 0.021 | 0.088 |
| 233.062 | 2.474 | Unidentified | 0.003 | 0.033 | 0.087 |
| 458.692 | 17.498 | 7-methylguanosine 5'-diphosphate | 0.002 | 0.028 | 0.086 |
| 651.220 | 6.531 | Unidentified | 0.007 | 0.047 | 0.083 |
| 174.015 | 1.671 | Adenine | 0.015 | 0.065 | 0.081 |
| 105.047 | 4.298 | Malonic acid | 0.036 | 0.107 | 0.079 |
| 158.115 | 12.322 | Unidentified | 0.010 | 0.052 | 0.076 |
| 242.062 | 7.529 | N-(2,3-Dihydroxybenzoyl)-L-serine | 0.006 | 0.043 | 0.074 |
| 190.991 | 1.143 | Quinol sulfate | 0.023 | 0.081 | 0.072 |
| 442.388 | 21.800 | Leukotriene E3 | 0.036 | 0.107 | 0.071 |
| 283.085 | 7.454 | Abu-Ala-OH | 0.009 | 0.050 | 0.069 |
| 454.387 | 21.954 | PC(O-12:0/O-2:0) | 0.043 | 0.121 | 0.060 |
| 74.060 | 1.290 | Unidentified | 0.001 | 0.022 | 0.060 |
| 189.071 | 16.249 | Unidentified | 0.028 | 0.092 | 0.058 |
| 340.260 | 11.485 | Unidentified | 0.008 | 0.047 | 0.057 |
| 262.321 | 1.402 | Unidentified | 0.016 | 0.067 | 0.056 |
| 315.151 | 1.067 | Unidentified | 0.020 | 0.076 | 0.053 |
| 398.242 | 20.069 | PC (9:0/0:0) | 0.010 | 0.053 | 0.050 |
| 542.188 | 4.300 | cyclic adenosine diphosphate ribose | 0.021 | 0.076 | 0.046 |
| 80.947 | 0.872 | Pyridine | 0.008 | 0.047 | 0.040 |
| 552.182 | 1.974 | N-Acetyl-N6, N6,O-tridemethylpuromycin-5'-phosphate | 0.001 | 0.021 | 0.038 |
| 308.169 | 5.486 | Unidentified | 0.024 | 0.084 | 0.035 |
| 198.113 | 1.405 | Metanephrine | 0.004 | 0.036 | 0.035 |
| 168.079 | 6.753 | N-Trimethyl-2-aminoethylphosphonate | 0.010 | 0.053 | 0.032 |
| 674.429 | 19.078 | Unidentified | 0.015 | 0.065 | 0.031 |
| 534.192 | 4.296 | Unidentified | 0.031 | 0.098 | 0.030 |
| 586.377 | 19.016 | Unidentified | 0.021 | 0.077 | 0.029 |
| 564.362 | 15.690 | PC (18:1(9E)/2:0) | 0.015 | 0.067 | 0.023 |
| 218.065 | 6.752 | 2-(Hydroxymethyl)-3-(acetamidomethylene)succinate | 0.007 | 0.046 | 0.023 |
| 144.079 | 5.017 | Unidentified | 0.000 | 0.021 | 0.018 |
| 555.127 | 1.776 | 3-O-(6-O-alpha-D-Xylosylphospho-alpha-D-mannopyranosyl)-alpha-D-mannopyranose | 0.018 | 0.072 | 0.017 |
| 151.062 | 5.364 | 1-Methylhypoxanthine | 0.007 | 0.046 | 0.013 |
| 153.041 | 2.293 | Xanthine | 0.001 | 0.021 | 0.012 |
| 183.091 | 6.753 | Unidentified | 0.033 | 0.102 | 0.011 |
| 90.054 | 2.340 | Alanine | 0.003 | 0.031 | 0.007 |
| 86.096 | 4.679 | 2-Methylpyrrolidine | 0.002 | 0.026 | 0.006 |
| 229.095 | 6.755 | Unidentified | 0.006 | 0.043 | 0.003 |
| 197.010 | 18.642 | Allantoin | 0.007 | 0.046 | 0.001 |
| 159.025 | 1.820 | fumarylacetic acid | 0.000 | 0.021 | 0.000 |
| 239.108 | 2.187 | HEPES | 0.031 | 0.098 | -0.002 |
| 421.425 | 20.069 | 27:2(5Z,9Z) (25Me) | 0.009 | 0.050 | -0.003 |
| 637.234 | 6.533 | Unidentified | 0.022 | 0.079 | -0.012 |
| 303.135 | 6.743 | Unidentified | 0.002 | 0.026 | -0.012 |
| 280.312 | 1.409 | Unidentified | 0.033 | 0.102 | -0.013 |
| 337.021 | 14.018 | Unidentified | 0.000 | 0.021 | -0.016 |
| 526.428 | 22.055 | Unidentified | 0.002 | 0.026 | -0.019 |
| 94.042 | 2.222 | Unidentified | 0.003 | 0.033 | -0.019 |
| 186.074 | 2.569 | Unidentified | 0.004 | 0.036 | -0.019 |
| 91.056 | 2.221 | Unidentified | 0.000 | 0.021 | -0.031 |
| 77.039 | 14.015 | Unidentified | 0.048 | 0.129 | -0.034 |
| 274.090 | 1.944 | Pentose + Proline | 0.000 | 0.021 | -0.037 |
| 154.081 | 1.974 | Dopamine | 0.016 | 0.067 | -0.040 |
| 254.099 | 8.874 | Dihydroferuloylglycine | 0.001 | 0.022 | -0.042 |
| 103.111 | 4.294 | 3-Methylpentan-1-ol | 0.018 | 0.072 | -0.044 |
| 220.096 | 14.010 | Unidentified | 0.026 | 0.088 | -0.045 |
| 326.219 | 2.700 | Farnesylcysteine | 0.016 | 0.068 | -0.051 |
| 84.043 | 4.709 | 1-Methylpyrrolinium | 0.006 | 0.043 | -0.059 |
| 773.492 | 20.068 | PA (20:2(11Z,14Z)/22:6(4Z,7Z,10Z,13Z,16Z,19Z)) | 0.019 | 0.074 | -0.068 |
| 447.199 | 1.986 | Unidentified | 0.027 | 0.089 | -0.071 |
| 387.177 | 19.739 | 3,5-Di-O-methyl-8-prenylafzelechin-4beta-ol | 0.021 | 0.076 | -0.079 |
| 175.088 | 3.096 | Unidentified | 0.040 | 0.113 | -0.079 |
| 175.120 | 1.047 | L-Arginine | 0.016 | 0.067 | -0.080 |
| 246.112 | 5.034 | Asparaginyl-Hydroxyproline | 0.005 | 0.038 | -0.080 |
| 276.091 | 1.797 | Unidentified | 0.035 | 0.104 | -0.081 |
| 768.538 | 20.069 | PE (18:0/20:4(5Z,8Z,11Z,14Z))[U] | 0.027 | 0.090 | -0.085 |
| 380.163 | 18.682 | Unidentified | 0.025 | 0.085 | -0.087 |
| 330.058 | 1.959 | cAMP | 0.000 | 0.017 | -0.089 |
| 581.256 | 1.371 | Unidentified | 0.007 | 0.046 | -0.093 |
| 195.136 | 18.684 | Unidentified | 0.006 | 0.044 | -0.093 |
| 249.055 | 3.655 | Unidentified | 0.027 | 0.090 | -0.093 |
| 181.067 | 18.651 | Unidentified | 0.019 | 0.073 | -0.095 |
| 663.444 | 22.730 | Prostaglandin E2-biotin | 0.003 | 0.031 | -0.100 |
| 156.079 | 6.748 | Histidine | 0.015 | 0.065 | -0.105 |
| 436.345 | 21.150 | Unidentified | 0.006 | 0.045 | -0.108 |
| 162.039 | 18.676 | N-Formyl-L-aspartate | 0.001 | 0.021 | -0.111 |
| 374.060 | 1.833 | Unidentified | 0.003 | 0.031 | -0.112 |
| 165.055 | 2.221 | Unidentified | 0.005 | 0.038 | -0.115 |
| 480.423 | 22.436 | PC(O-16:1(9Z)/0:0) [U] | 0.034 | 0.102 | -0.117 |
| 307.108 | 16.256 | Glutathione amide | 0.028 | 0.093 | -0.117 |
| 145.048 | 4.997 | 3-hexenedioic acid | 0.000 | 0.011 | -0.117 |
| 316.133 | 3.219 | Unidentified | 0.010 | 0.052 | -0.118 |
| 96.960 | 4.669 | Methaneselenol | 0.022 | 0.080 | -0.129 |
| 265.020 | 2.473 | Unidentified | 0.011 | 0.056 | -0.131 |
| 267.056 | 2.585 | Unidentified | 0.003 | 0.033 | -0.131 |
| 194.080 | 2.701 | Phenylacetylglycine | 0.002 | 0.026 | -0.132 |
| 464.356 | 22.202 | D-Glucosyldihydrosphingosine | 0.006 | 0.043 | -0.138 |
| 493.198 | 2.561 | Unidentified | 0.001 | 0.022 | -0.140 |
| 260.113 | 1.069 | Unidentified | 0.036 | 0.106 | -0.149 |
| 543.154 | 0.745 | Unidentified | 0.015 | 0.067 | -0.152 |
| 72.082 | 1.092 | Unidentified | 0.031 | 0.098 | -0.154 |
| 630.402 | 19.036 | Unidentified | 0.003 | 0.031 | -0.159 |
| 127.038 | 3.255 | Unidentified | 0.006 | 0.043 | -0.166 |
| 202.108 | 1.394 | (E)-1-(4-methylquinazolin-2(1H)-ylidene) guanidine | 0.001 | 0.021 | -0.168 |
| 331.127 | 6.761 | Unidentified | 0.011 | 0.055 | -0.168 |
| 369.215 | 0.974 | Unidentified | 0.044 | 0.122 | -0.171 |
| 314.084 | 1.945 | Unidentified | 0.001 | 0.021 | -0.174 |
| 332.115 | 6.748 | L, L-Cyclo(leucylprolyl) | 0.041 | 0.115 | -0.176 |
| 302.119 | 1.350 | Unidentified | 0.001 | 0.021 | -0.176 |
| 244.099 | 5.041 | Cytidine | 0.040 | 0.113 | -0.180 |
| 174.111 | 3.299 | Hexanoylglycine | 0.003 | 0.033 | -0.180 |
| 103.054 | 6.539 | Unidentified | 0.018 | 0.072 | -0.185 |
| 344.135 | 2.720 | b-D-Glucopyranosiduronic acid | 0.003 | 0.033 | -0.186 |
| 136.113 | 23.292 | Unidentified | 0.028 | 0.093 | -0.197 |
| 272.085 | 18.623 | Unidentified | 0.008 | 0.049 | -0.210 |
| 95.050 | 2.221 | Phenol | 0.013 | 0.061 | -0.216 |
| 258.133 | 2.437 | Unidentified | 0.010 | 0.053 | -0.222 |
| 123.046 | 2.221 | THIODIGLYCOL | 0.006 | 0.045 | -0.226 |
| 160.095 | 1.401 | N-isovalerylglycine | 0.006 | 0.043 | -0.228 |
| 130.202 | 1.696 | Octylamine | 0.009 | 0.050 | -0.235 |
| 136.078 | 2.221 | 2-Phenylacetamide | 0.005 | 0.043 | -0.237 |
| 198.070 | 7.524 | Unidentified | 0.001 | 0.021 | -0.238 |
| 312.114 | 1.790 | Lys-Gly-OH | 0.038 | 0.110 | -0.243 |
| 280.115 | 2.727 | Unidentified | 0.007 | 0.046 | -0.248 |
| 109.064 | 2.220 | Unidentified | 0.000 | 0.011 | -0.253 |
| 381.147 | 1.646 | 2-Methoxyestrone 3-sulfate | 0.031 | 0.098 | -0.255 |
| 119.048 | 2.221 | Allophanic acid methyl ester | 0.004 | 0.037 | -0.262 |
| 105.043 | 6.540 | 3-Cyanopyridine | 0.006 | 0.045 | -0.264 |
| 228.101 | 5.025 | Unidentified | 0.013 | 0.060 | -0.264 |
| 107.052 | 4.299 | Unidentified | 0.001 | 0.024 | -0.269 |
| 258.110 | 6.751 | Glycerophosphocholine | 0.020 | 0.076 | -0.272 |
| 209.128 | 1.081 | Unidentified | 0.005 | 0.039 | -0.284 |
| 175.007 | 4.312 | Unidentified | 0.035 | 0.106 | -0.289 |
| 426.393 | 22.298 | Vaccenyl carnitine | 0.035 | 0.105 | -0.290 |
| 85.029 | 1.293 | Unidentified | 0.006 | 0.043 | -0.295 |
| 221.092 | 6.552 | 5-Hydroxy-L-tryptophan | 0.026 | 0.087 | -0.296 |
| 167.094 | 1.133 | N-Formyl-4-amino-5-aminomethyl-2-methylpyrimidine | 0.049 | 0.132 | -0.296 |
| 542.357 | 18.984 | PC (20:5(5Z,8Z,11Z,14Z,17Z)/0:0) | 0.001 | 0.021 | -0.316 |
| 447.134 | 6.534 | Unidentified | 0.034 | 0.102 | -0.318 |
| 570.450 | 22.046 | LysoPC(22:5(4Z,7Z,10Z,13Z,16Z)) | 0.047 | 0.127 | -0.323 |
| 454.113 | 9.043 | Unidentified | 0.029 | 0.093 | -0.329 |
| 107.052 | 2.224 | Unidentified | 0.001 | 0.021 | -0.330 |
| 550.210 | 1.783 | Unidentified | 0.048 | 0.129 | -0.343 |
| 124.074 | 5.778 | Unidentified | 0.034 | 0.102 | -0.347 |
| 280.139 | 1.403 | Unidentified | 0.008 | 0.047 | -0.347 |
| 440.369 | 21.718 | PC(O-12:0/O-1:0) | 0.004 | 0.036 | -0.351 |
| 786.205 | 6.741 | Flavin adenine dinucleotide (FAD) | 0.012 | 0.058 | -0.352 |
| 369.110 | 4.301 | Unidentified | 0.002 | 0.030 | -0.357 |
| 105.000 | 1.281 | β-Hydroxypyruvic acid | 0.001 | 0.021 | -0.362 |
| 371.144 | 4.847 | 4beta-Hydroxyobovatachromene | 0.006 | 0.043 | -0.362 |
| 126.960 | 19.775 | Unidentified | 0.021 | 0.076 | -0.366 |
| 515.172 | 15.768 | TyrMe-Nap-OH | 0.017 | 0.071 | -0.369 |
| 139.004 | 1.562 | Unidentified | 0.012 | 0.059 | -0.373 |
| 134.998 | 1.192 | Malic acid | 0.001 | 0.022 | -0.377 |
| 101.004 | 1.135 | Sodium fluoroacetate | 0.015 | 0.065 | -0.384 |
| 597.140 | 2.214 | Unidentified | 0.012 | 0.056 | -0.388 |
| 891.311 | 1.402 | dipalmitoyl phosphatidylinositol 3-phosphate | 0.027 | 0.090 | -0.398 |
| 247.045 | 2.470 | Glutamyl-Threonine | 0.031 | 0.098 | -0.398 |
| 166.412 | 4.299 | Unidentified | 0.019 | 0.073 | -0.398 |
| 205.985 | 1.207 | Unidentified | 0.009 | 0.050 | -0.401 |
| 334.090 | 1.796 | [3,4,5-trihydroxy-6-(hydroxymethyl)oxan-2-yl] 9-(hydroxymethyl)-2,2,6a,6b,9,12a-hexamethyl-10-[3,4,5-trihydroxy-6-[(3,4,5-trihydroxy-6-methyloxan-2-yl)oxymethyl]oxan-2-yl]oxy-1,3,4,5,6,6a,7,8,8a,10,11,12,13,14b-tetradecahydropicene-4a-carboxylate | 0.002 | 0.028 | -0.402 |
| 106.048 | 1.282 | L-Serine | 0.002 | 0.028 | -0.405 |
| 388.158 | 1.269 | Unidentified | 0.002 | 0.025 | -0.407 |
| 221.092 | 4.891 | 5-Hydroxytryptophan | 0.019 | 0.073 | -0.419 |
| 152.038 | 0.774 | Unidentified | 0.005 | 0.043 | -0.423 |
| 277.064 | 0.770 | Unidentified | 0.038 | 0.110 | -0.427 |
| 292.203 | 20.069 | Unidentified | 0.009 | 0.050 | -0.444 |
| 103.054 | 14.021 | Unidentified | 0.039 | 0.113 | -0.457 |
| 134.060 | 1.848 | Indoxyl | 0.000 | 0.007 | -0.457 |
| 482.422 | 22.119 | Ceramide (d18:1/12:0) | 0.011 | 0.055 | -0.457 |
| 384.343 | 22.737 | N-stearoyl valine | 0.038 | 0.111 | -0.459 |
| 102.056 | 1.280 | Unidentified | 0.002 | 0.026 | -0.462 |
| 264.121 | 5.021 | 7-Mercaptoheptanoylthreonine | 0.001 | 0.021 | -0.463 |
| 758.452 | 19.030 | PE (18:4(6Z,9Z,12Z,15Z)/20:5(5Z,8Z,11Z,14Z,17Z)) | 0.004 | 0.036 | -0.467 |
| 204.103 | 5.009 | 5-(2-Furanyl)-1,2,3,4,5,6-hexahydro-7H-cyclopenta[b]pyridin-7-one | 0.023 | 0.081 | -0.473 |
| 334.140 | 10.201 | Unidentified | 0.001 | 0.024 | -0.478 |
| 190.109 | 8.857 | Unidentified | 0.002 | 0.026 | -0.483 |
| 123.107 | 2.228 | Unidentified | 0.001 | 0.021 | -0.487 |
| 280.231 | 1.408 | Linoleamide | 0.013 | 0.061 | -0.493 |
| 708.576 | 22.743 | PS(O-16:0/O-16:0) [U] | 0.021 | 0.078 | -0.493 |
| 276.412 | 3.268 | Arginyl-Threonine | 0.015 | 0.067 | -0.495 |
| 778.209 | 2.210 | PS (14:1(9Z)/22:6(4Z,7Z,10Z,13Z,16Z,19Z)) | 0.004 | 0.038 | -0.498 |
| 104.054 | 1.283 | Biuret | 0.000 | 0.017 | -0.499 |
| 412.377 | 22.049 | PC(O-8:0/2:0) | 0.006 | 0.043 | -0.500 |
| 283.069 | 0.720 | 9-Riburonosylhypoxanthine | 0.000 | 0.010 | -0.517 |
| 262.130 | 1.401 | Unidentified | 0.008 | 0.047 | -0.535 |
| 80.048 | 1.845 | Unidentified | 0.038 | 0.110 | -0.539 |
| 182.083 | 2.208 | L-Tyrosine | 0.001 | 0.024 | -0.549 |
| 262.214 | 1.401 | Unidentified | 0.018 | 0.072 | -0.562 |
| 751.517 | 20.069 | PA (18:1(9Z)/22:4(7Z,10Z,13Z,16Z)) | 0.037 | 0.109 | -0.581 |
| 150.058 | 1.281 | L-Methionine | 0.000 | 0.004 | -0.582 |
| 714.235 | 4.297 | PS (13:0/18:4(6Z,9Z,12Z,15Z)) | 0.020 | 0.075 | -0.583 |
| 157.044 | 7.526 | Unidentified | 0.005 | 0.039 | -0.584 |
| 78.035 | 1.559 | Cysteamine | 0.040 | 0.113 | -0.617 |
| 217.070 | 1.618 | Bisnorbiotin | 0.002 | 0.030 | -0.627 |
| 393.283 | 20.069 | Unidentified | 0.023 | 0.082 | -0.644 |
| 261.146 | 7.254 | L-gamma-glutamyl-L-isoleucine | 0.017 | 0.069 | -0.647 |
| 582.231 | 2.702 | Unidentified | 0.048 | 0.129 | -0.659 |
| 617.243 | 16.215 | Unidentified | 0.004 | 0.036 | -0.675 |
| 404.203 | 19.741 | 16-phenoxy tetranor PGF2α methyl amide | 0.027 | 0.090 | -0.677 |
| 477.177 | 5.771 | Calcium pantothenate | 0.003 | 0.033 | -0.718 |
| 306.115 | 1.430 | Unidentified | 0.030 | 0.097 | -0.731 |
| 670.398 | 18.996 | PE (13:0/18:4(6Z,9Z,12Z,15Z)) | 0.001 | 0.024 | -0.737 |
| 194.119 | 3.935 | Unidentified | 0.009 | 0.050 | -0.738 |
| 238.071 | 1.920 | Unidentified | 0.038 | 0.110 | -0.745 |
| 192.101 | 14.021 | Unidentified | 0.029 | 0.095 | -0.749 |
| 161.068 | 3.290 | N-Nitrosohydroxyproline | 0.010 | 0.052 | -0.779 |
| 598.496 | 22.068 | 14:0 Cholesteryl ester | 0.038 | 0.111 | -0.794 |
| 120.079 | 14.016 | L-Threonine | 0.017 | 0.070 | -0.805 |
| 200.089 | 1.793 | 2-Acetyl-1,5,6,7-tetrahydro-6-hydroxy-7-(hydroxymethyl)-4H-azepine-4-one | 0.024 | 0.083 | -0.821 |
| 115.956 | 1.211 | 3-keto-2-methylbutyrate | 0.021 | 0.076 | -0.853 |
| 335.108 | 12.123 | (S)-a-Amino-2,5-dihydro-5-oxo-4-isoxazolepropanoic acid N2-glucoside | 0.046 | 0.127 | -0.855 |
| 334.975 | 1.198 | Unidentified | 0.011 | 0.056 | -0.934 |
| 112.051 | 1.268 | Cytosine | 0.000 | 0.010 | -0.939 |
| 225.088 | 0.801 | 3-Hydroxy-DL-kynurenine | 0.001 | 0.024 | -0.956 |
| 724.504 | 22.768 | PC (14:1(9Z)/18:4(6Z,9Z,12Z,15Z)) | 0.046 | 0.125 | -0.973 |
| 328.427 | 5.010 | Stearoylethanolamide | 0.013 | 0.061 | -1.024 |
| 233.062 | 2.469 | Unidentified | 0.000 | 0.010 | -1.032 |
| 302.185 | 20.069 | Unidentified | 0.013 | 0.061 | -1.049 |
| 375.995 | 1.654 | Unidentified | 0.021 | 0.076 | -1.062 |
| 301.145 | 2.018 | Unidentified | 0.008 | 0.047 | -1.086 |
| 133.964 | 1.201 | Unidentified | 0.004 | 0.034 | -1.117 |
| 292.119 | 5.037 | Unidentified | 0.002 | 0.026 | -1.132 |
| 144.100 | 3.198 | Unidentified | 0.028 | 0.093 | -1.168 |
| 203.052 | 1.032 | Unidentified | 0.017 | 0.069 | -1.169 |
| 250.109 | 5.008 | Cysteinyl-Lysine | 0.002 | 0.028 | -1.193 |
| 421.319 | 20.069 | Unidentified | 0.021 | 0.076 | -1.201 |
| 149.017 | 2.472 | 2-Oxo-4-methylthiobutanoic acid | 0.017 | 0.070 | -1.310 |
| 113.108 | 3.814 | Hydroxymethylphosphonate | 0.001 | 0.021 | -1.529 |
| 113.107 | 5.786 | 2-Imino-4-methylpiperidine | 0.001 | 0.024 | -1.635 |
| 282.132 | 5.023 | Unidentified | 0.005 | 0.038 | -1.649 |
| 350.119 | 20.060 | Unidentified | 0.031 | 0.098 | -1.890 |
| 376.257 | 20.069 | Unidentified | 0.016 | 0.069 | -1.906 |
| 759.271 | 0.771 | Unidentified | 0.006 | 0.043 | -2.289 |

**Table S2.** Significantly Altered Metabolites in SAECs Exposed to 5 µg/mL PM Collected from the Printer Room During Printing with ABS Filaments.

| m/z | rt | Metabolite Name | p.value | FDR | log2(FC) |
| --- | --- | --- | --- | --- | --- |
| 284.209 | 13.296 | Unidentified | 0.000 | 0.009 | 3.313 |
| 267.181 | 13.297 | Unidentified | 0.000 | 0.009 | 3.168 |
| 289.161 | 13.294 | Arginyl-Asparagine | 0.000 | 0.009 | 3.049 |
| 387.177 | 19.739 | 3,5-Di-O-methyl-8-prenylafzelechin-4beta-ol | 0.006 | 0.052 | 3.005 |
| 404.203 | 19.741 | 16-phenoxy tetranor PGF2α methyl amide | 0.006 | 0.052 | 2.776 |
| 103.075 | 13.296 | 3-Methyl-2-butene-1-thiol | 0.000 | 0.010 | 2.724 |
| 432.238 | 19.741 | 17-phenoxy trinor PGF2α ethyl amide | 0.009 | 0.064 | 2.715 |
| 87.042 | 12.443 | 4-Deoxytetronic acid | 0.011 | 0.072 | 2.455 |
| 493.198 | 2.561 | Unidentified | 0.002 | 0.035 | 2.017 |
| 240.182 | 11.679 | 2-Octyl-4-propylthiazole | 0.001 | 0.032 | 2.015 |
| 223.153 | 11.679 | Unidentified | 0.001 | 0.028 | 1.933 |
| 268.209 | 11.683 | (2R,5S)-2,5-di((E)-pent-2-en-4-yn-1-yl) decahydroquinoline | 0.003 | 0.042 | 1.795 |
| 245.136 | 11.679 | Polyethylene, oxidized | 0.001 | 0.028 | 1.738 |
| 105.071 | 19.736 | L-2,3-DIAMINOPROPIONIC ACID | 0.018 | 0.091 | 1.718 |
| 285.090 | 8.626 | Cysteinyl-Tyrosine | 0.009 | 0.067 | 1.710 |
| 103.075 | 13.294 | Isovaleric acid | 0.001 | 0.032 | 1.682 |
| 261.086 | 2.163 | Unidentified | 0.001 | 0.024 | 1.646 |
| 137.047 | 1.820 | Hypoxanthine | 0.004 | 0.045 | 1.593 |
| 236.112 | 2.569 | N-(9-oxodecyl) acetamide | 0.002 | 0.033 | 1.575 |
| 159.025 | 1.820 | fumarylacetic acid | 0.003 | 0.040 | 1.573 |
| 163.110 | 14.364 | 3-hydroxy-3-methyl-Glutaric acid | 0.006 | 0.050 | 1.531 |
| 147.103 | 11.681 | 2-hydroxy enanthoic acid | 0.001 | 0.032 | 1.510 |
| 608.386 | 16.452 | Unidentified | 0.006 | 0.050 | 1.507 |
| 708.576 | 22.743 | PS(O-16:0/O-16:0) [U] | 0.003 | 0.039 | 1.484 |
| 258.094 | 2.561 | Unidentified | 0.002 | 0.033 | 1.450 |
| 153.041 | 2.293 | Xanthine | 0.002 | 0.034 | 1.437 |
| 110.036 | 2.300 | Hypotaurine | 0.007 | 0.053 | 1.398 |
| 186.074 | 2.569 | Unidentified | 0.005 | 0.050 | 1.368 |
| 217.065 | 16.239 | Unidentified | 0.008 | 0.058 | 1.331 |
| 344.238 | 2.705 | Unidentified | 0.011 | 0.070 | 1.267 |
| 268.136 | 11.140 | Unidentified | 0.001 | 0.028 | 1.249 |
| 663.444 | 22.730 | Prostaglandin E2-biotin | 0.012 | 0.075 | 1.223 |
| 564.362 | 15.690 | PC (18:1(9E)/2:0) | 0.006 | 0.051 | 1.133 |
| 520.332 | 14.860 | 1-Linoleoylglycerophosphocholine | 0.034 | 0.129 | 1.060 |
| 781.252 | 0.776 | N, N'-Diacetylchitobiosyldiphosphodolichol | 0.010 | 0.067 | 1.057 |
| 113.034 | 2.589 | Uracil | 0.003 | 0.039 | 1.026 |
| 217.070 | 1.618 | Bisnorbiotin | 0.012 | 0.074 | 1.020 |
| 708.511 | 22.725 | PS(O-16:0/15:0) | 0.027 | 0.114 | 1.002 |
| 455.166 | 0.981 | Phe-Phe4Cl-OH | 0.002 | 0.034 | 0.931 |
| 306.115 | 1.430 | Unidentified | 0.004 | 0.045 | 0.915 |
| 187.032 | 18.681 | Unidentified | 0.004 | 0.045 | 0.880 |
| 247.138 | 1.632 | L-N2-(2-Carboxyethyl) arginine | 0.020 | 0.097 | 0.880 |
| 521.170 | 0.772 | Unidentified | 0.016 | 0.086 | 0.875 |
| 324.131 | 1.430 | Unidentified | 0.005 | 0.050 | 0.868 |
| 693.216 | 5.019 | Unidentified | 0.001 | 0.024 | 0.839 |
| 549.343 | 1.992 | 5beta-scymnol sulfate | 0.005 | 0.050 | 0.823 |
| 418.252 | 1.990 | 16-phenoxy Prostaglandin F2a ethyl amide | 0.013 | 0.076 | 0.800 |
| 637.234 | 6.533 | Unidentified | 0.008 | 0.061 | 0.794 |
| 679.234 | 5.035 | Unidentified | 0.000 | 0.010 | 0.784 |
| 555.127 | 1.776 | 3-O-(6-O-alpha-D-Xylosylphospho-alpha-D-mannopyranosyl)-alpha-D-mannopyranose | 0.019 | 0.094 | 0.769 |
| 496.474 | 21.797 | PS (16:0/0:0) | 0.037 | 0.135 | 0.765 |
| 165.112 | 6.665 | Unidentified | 0.002 | 0.034 | 0.761 |
| 520.199 | 4.299 | Unidentified | 0.000 | 0.014 | 0.728 |
| 301.061 | 1.799 | Unidentified | 0.005 | 0.050 | 0.720 |
| 301.145 | 2.018 | Unidentified | 0.018 | 0.091 | 0.714 |
| 412.095 | 1.755 | Unidentified | 0.023 | 0.106 | 0.696 |
| 297.038 | 1.765 | Unidentified | 0.011 | 0.072 | 0.683 |
| 267.056 | 2.585 | Unidentified | 0.001 | 0.032 | 0.678 |
| 309.226 | 17.938 | Unidentified | 0.000 | 0.013 | 0.658 |
| 240.123 | 10.723 | Unidentified | 0.009 | 0.064 | 0.653 |
| 394.350 | 22.080 | N-palmitoyl histidine | 0.011 | 0.070 | 0.650 |
| 447.134 | 6.534 | Unidentified | 0.003 | 0.042 | 0.636 |
| 238.071 | 1.920 | Unidentified | 0.027 | 0.115 | 0.630 |
| 651.220 | 6.531 | Unidentified | 0.005 | 0.048 | 0.619 |
| 426.079 | 1.749 | Hyaluronic acid | 0.014 | 0.080 | 0.608 |
| 562.203 | 1.318 | Unidentified | 0.022 | 0.104 | 0.607 |
| 685.284 | 4.297 | gamma-L-Glutamyl-butirosin B | 0.001 | 0.028 | 0.606 |
| 247.045 | 2.470 | Glutamyl-Threonine | 0.046 | 0.157 | 0.594 |
| 541.133 | 1.751 | Unidentified | 0.026 | 0.113 | 0.589 |
| 611.263 | 3.300 | Unidentified | 0.002 | 0.034 | 0.586 |
| 534.192 | 4.296 | Unidentified | 0.000 | 0.009 | 0.584 |
| 324.106 | 1.310 | Unidentified | 0.017 | 0.088 | 0.545 |
| 340.260 | 11.485 | Unidentified | 0.002 | 0.034 | 0.534 |
| 322.248 | 11.494 | Anandamide (18:3, n-6) | 0.011 | 0.072 | 0.527 |
| 453.339 | 14.548 | (17E)-1α,25-dihydroxy-26,27-dimethyl-17,20,22,22,23,23-hexadehydro-24a-homovitamin D3 / (17E)-1α,25-dihydroxy-26,27-dimethyl-17,20,22,22,23,23-hexadehydro-24a-homocholecalciferol | 0.018 | 0.091 | 0.523 |
| 168.065 | 1.436 | Pyridoxal (Vitamin B6) | 0.040 | 0.143 | 0.509 |
| 746.561 | 21.967 | PC (15:0/18:1(9Z))[U] | 0.005 | 0.050 | 0.505 |
| 369.110 | 4.301 | Unidentified | 0.000 | 0.022 | 0.491 |
| 249.055 | 3.655 | Unidentified | 0.003 | 0.044 | 0.476 |
| 482.422 | 22.119 | Ceramide (d18:1/12:0) | 0.033 | 0.129 | 0.475 |
| 205.028 | 1.227 | 3-phenyllactic acid | 0.019 | 0.094 | 0.472 |
| 438.372 | 22.072 | (±) N-(1-methyl-2-hydroxy-2-phenyl-ethyl) arachidonyl amine | 0.027 | 0.114 | 0.468 |
| 86.096 | 4.679 | 2-Methylpyrrolidine | 0.004 | 0.046 | 0.467 |
| 362.241 | 11.485 | 3-Oxohexadecanoic acid glycerides | 0.005 | 0.049 | 0.463 |
| 545.210 | 1.276 | Estriol 3-sulfate 16-glucuronide | 0.032 | 0.126 | 0.460 |
| 540.216 | 1.242 | Unidentified | 0.049 | 0.163 | 0.452 |
| 582.174 | 2.205 | Unidentified | 0.001 | 0.031 | 0.437 |
| 424.162 | 16.360 | Unidentified | 0.019 | 0.094 | 0.427 |
| 400.342 | 21.112 | Palmitoyl-L-carnitine | 0.022 | 0.101 | 0.404 |
| 274.107 | 12.258 | L-Thyronine | 0.000 | 0.024 | 0.385 |
| 338.343 | 21.906 | Unidentified | 0.033 | 0.129 | 0.384 |
| 227.173 | 7.008 | 1,8-Diazacyclotetradecane-2,9-dione | 0.024 | 0.106 | 0.375 |
| 548.170 | 2.168 | Unidentified | 0.006 | 0.052 | 0.366 |
| 628.504 | 22.394 | Cer(t18:0/20:0(2OH)) | 0.019 | 0.094 | 0.362 |
| 482.401 | 22.062 | PC(O-15:0/O-1:0)[U] | 0.017 | 0.088 | 0.361 |
| 210.044 | 7.521 | Unidentified | 0.044 | 0.151 | 0.356 |
| 317.118 | 2.000 | Unidentified | 0.029 | 0.120 | 0.351 |
| 157.044 | 7.526 | Unidentified | 0.017 | 0.088 | 0.351 |
| 410.105 | 1.745 | 1-(5-Phosphoribosyl)-4-(N-succinocarboxamide)-5-aminoimidazole | 0.017 | 0.088 | 0.336 |
| 348.987 | 1.790 | Unidentified | 0.045 | 0.155 | 0.333 |
| 454.387 | 21.954 | PC(O-12:0/O-2:0) | 0.044 | 0.151 | 0.330 |
| 514.410 | 21.803 | Sulfolithocholylglycine | 0.038 | 0.139 | 0.327 |
| 258.066 | 5.776 | Unidentified | 0.008 | 0.058 | 0.325 |
| 197.010 | 18.642 | Allantoin | 0.020 | 0.094 | 0.323 |
| 512.430 | 21.816 | PS(O-18:0/0:0) | 0.040 | 0.143 | 0.316 |
| 304.098 | 0.806 | Unidentified | 0.037 | 0.135 | 0.307 |
| 198.070 | 7.524 | Unidentified | 0.012 | 0.074 | 0.300 |
| 542.188 | 4.300 | cyclic adenosine diphosphate ribose | 0.005 | 0.049 | 0.299 |
| 220.081 | 6.735 | O-Succinyl-L-homoserine | 0.044 | 0.151 | 0.296 |
| 477.177 | 5.771 | Calcium pantothenate | 0.031 | 0.125 | 0.278 |
| 442.388 | 21.800 | Leukotriene E3 | 0.005 | 0.050 | 0.271 |
| 321.144 | 6.739 | Unidentified | 0.036 | 0.135 | 0.269 |
| 440.369 | 21.718 | PC(O-12:0/O-1:0) | 0.000 | 0.013 | 0.258 |
| 305.148 | 6.735 | Unidentified | 0.046 | 0.156 | 0.255 |
| 614.482 | 22.033 | Ferroxamine | 0.036 | 0.135 | 0.239 |
| 70.074 | 1.961 | 1-Pyrroline | 0.014 | 0.080 | 0.237 |
| 526.428 | 22.055 | Unidentified | 0.029 | 0.120 | 0.222 |
| 154.081 | 1.974 | Dopamine | 0.029 | 0.120 | 0.220 |
| 114.046 | 6.538 | Unidentified | 0.000 | 0.024 | 0.209 |
| 248.095 | 1.793 | Glutamyl-Threonine | 0.049 | 0.163 | 0.200 |
| 140.107 | 3.271 | Unidentified | 0.018 | 0.092 | 0.194 |
| 132.102 | 2.014 | L-Isoleucine | 0.003 | 0.044 | 0.190 |
| 72.082 | 1.092 | Unidentified | 0.029 | 0.120 | 0.185 |
| 267.096 | 9.892 | Unidentified | 0.024 | 0.106 | 0.183 |
| 244.099 | 5.041 | Cytidine | 0.012 | 0.074 | 0.182 |
| 144.079 | 5.017 | Unidentified | 0.001 | 0.032 | 0.179 |
| 234.077 | 6.367 | Unidentified | 0.018 | 0.091 | 0.172 |
| 89.037 | 6.539 | Pyruvate | 0.006 | 0.051 | 0.163 |
| 258.133 | 2.437 | Unidentified | 0.043 | 0.151 | 0.163 |
| 77.037 | 6.543 | Unidentified | 0.006 | 0.051 | 0.162 |
| 115.053 | 5.017 | Pyroterebic acid | 0.041 | 0.146 | 0.152 |
| 276.142 | 3.271 | Tryptophyl-Alanine | 0.011 | 0.070 | 0.151 |
| 248.150 | 3.232 | Malonylcarnitine | 0.026 | 0.113 | 0.150 |
| 294.156 | 3.258 | Unidentified | 0.013 | 0.076 | 0.149 |
| 107.047 | 5.018 | Unidentified | 0.004 | 0.045 | 0.146 |
| 276.211 | 3.270 | Unidentified | 0.027 | 0.114 | 0.143 |
| 230.137 | 3.246 | Unidentified | 0.005 | 0.049 | 0.138 |
| 105.047 | 4.298 | Malonic acid | 0.050 | 0.164 | 0.122 |
| 510.487 | 22.505 | Cer(d18:1/14:0) | 0.037 | 0.135 | -0.049 |
| 103.111 | 4.294 | 3-Methylpentan-1-ol | 0.025 | 0.108 | -0.052 |
| 256.082 | 1.914 | Nicotinate D-ribonucleoside | 0.038 | 0.137 | -0.089 |
| 166.204 | 4.293 | Unidentified | 0.032 | 0.126 | -0.108 |
| 202.108 | 1.394 | (E)-1-(4-methylquinazolin-2(1H)-ylidene) guanidine | 0.016 | 0.087 | -0.140 |
| 420.178 | 2.200 | Unidentified | 0.049 | 0.163 | -0.143 |
| 302.119 | 1.350 | Unidentified | 0.004 | 0.045 | -0.144 |
| 105.043 | 6.540 | 3-Cyanopyridine | 0.018 | 0.091 | -0.158 |
| 182.083 | 2.208 | L-Tyrosine | 0.032 | 0.126 | -0.165 |
| 603.102 | 0.831 | Unidentified | 0.012 | 0.074 | -0.177 |
| 603.204 | 0.962 | Unidentified | 0.039 | 0.141 | -0.185 |
| 95.050 | 2.221 | Phenol | 0.003 | 0.044 | -0.193 |
| 165.055 | 2.221 | Unidentified | 0.007 | 0.053 | -0.194 |
| 152.038 | 0.774 | Unidentified | 0.011 | 0.071 | -0.197 |
| 265.020 | 2.473 | Unidentified | 0.048 | 0.160 | -0.198 |
| 123.046 | 2.221 | THIODIGLYCOL | 0.002 | 0.034 | -0.198 |
| 136.078 | 2.221 | 2-Phenylacetamide | 0.005 | 0.048 | -0.200 |
| 119.048 | 2.221 | Allophanic acid methyl ester | 0.006 | 0.050 | -0.203 |
| 297.056 | 2.997 | Unidentified | 0.044 | 0.153 | -0.207 |
| 569.208 | 0.785 | Leukotriene F4 | 0.002 | 0.033 | -0.209 |
| 603.237 | 1.366 | Unidentified | 0.010 | 0.067 | -0.210 |
| 121.067 | 2.224 | 4-deoxy-threonic acid | 0.002 | 0.035 | -0.217 |
| 164.052 | 16.226 | 4-Hydroxy-L-glutamic acid | 0.024 | 0.108 | -0.220 |
| 97.970 | 2.871 | Unidentified | 0.048 | 0.160 | -0.224 |
| 189.071 | 16.249 | Unidentified | 0.035 | 0.131 | -0.227 |
| 280.139 | 1.403 | Unidentified | 0.012 | 0.074 | -0.230 |
| 98.962 | 19.790 | Phosphoric acid | 0.002 | 0.039 | -0.232 |
| 147.046 | 2.223 | Coumaric acid | 0.003 | 0.039 | -0.238 |
| 107.052 | 4.299 | Unidentified | 0.002 | 0.035 | -0.239 |
| 109.064 | 2.220 | Unidentified | 0.000 | 0.009 | -0.244 |
| 152.055 | 1.279 | Guanine | 0.031 | 0.125 | -0.246 |
| 284.110 | 1.362 | Glutamyl-Histidine | 0.005 | 0.050 | -0.249 |
| 140.000 | 7.520 | Unidentified | 0.003 | 0.040 | -0.250 |
| 142.086 | 1.408 | Unidentified | 0.036 | 0.135 | -0.251 |
| 331.001 | 1.670 | Unidentified | 0.044 | 0.151 | -0.252 |
| 91.056 | 2.221 | Unidentified | 0.002 | 0.034 | -0.253 |
| 547.230 | 0.780 | Unidentified | 0.038 | 0.138 | -0.259 |
| 355.066 | 18.653 | Phenolsulfonphthalein | 0.033 | 0.127 | -0.261 |
| 365.101 | 0.961 | Robustone | 0.041 | 0.146 | -0.262 |
| 107.052 | 2.224 | Unidentified | 0.008 | 0.058 | -0.262 |
| 143.056 | 1.404 | Unidentified | 0.029 | 0.120 | -0.262 |
| 104.054 | 1.283 | Biuret | 0.043 | 0.150 | -0.266 |
| 473.177 | 2.162 | C1'-C9-Glycosylated UWM6 | 0.002 | 0.033 | -0.268 |
| 398.987 | 0.896 | Unidentified | 0.002 | 0.034 | -0.269 |
| 167.094 | 1.133 | N-Formyl-4-amino-5-aminomethyl-2-methylpyrimidine | 0.036 | 0.135 | -0.269 |
| 114.091 | 7.095 | Unidentified | 0.001 | 0.024 | -0.275 |
| 601.108 | 0.825 | Protoporphyrin IX | 0.001 | 0.024 | -0.276 |
| 124.074 | 5.778 | Unidentified | 0.015 | 0.083 | -0.276 |
| 102.056 | 1.280 | Unidentified | 0.042 | 0.148 | -0.277 |
| 375.097 | 16.253 | Unidentified | 0.031 | 0.123 | -0.279 |
| 211.167 | 19.450 | 3E,5E-tridecadienoic acid | 0.019 | 0.094 | -0.285 |
| 160.095 | 1.401 | N-isovalerylglycine | 0.030 | 0.122 | -0.287 |
| 262.130 | 1.401 | Unidentified | 0.005 | 0.048 | -0.289 |
| 198.113 | 1.405 | Metanephrine | 0.001 | 0.032 | -0.293 |
| 130.202 | 1.696 | Octylamine | 0.002 | 0.033 | -0.295 |
| 103.054 | 6.539 | Unidentified | 0.003 | 0.039 | -0.297 |
| 284.058 | 16.265 | N2-Acetyl-L-aminoadipyl-δ-phosphate | 0.010 | 0.069 | -0.298 |
| 126.960 | 19.775 | Unidentified | 0.004 | 0.045 | -0.305 |
| 216.124 | 1.399 | Unidentified | 0.006 | 0.052 | -0.315 |
| 161.068 | 1.405 | Unidentified | 0.002 | 0.034 | -0.318 |
| 106.041 | 9.874 | 2-cyano-Pyrimidine | 0.020 | 0.094 | -0.326 |
| 518.237 | 1.366 | PS (18:4(6Z,9Z,12Z,15Z)/0:0) | 0.017 | 0.088 | -0.332 |
| 273.089 | 18.650 | Unidentified | 0.001 | 0.032 | -0.332 |
| 483.040 | 1.184 | Thymidine 5'-triphosphate | 0.022 | 0.101 | -0.335 |
| 524.077 | 1.377 | Unidentified | 0.036 | 0.135 | -0.346 |
| 94.042 | 2.222 | Unidentified | 0.001 | 0.028 | -0.355 |
| 319.035 | 5.778 | Unidentified | 0.013 | 0.076 | -0.358 |
| 261.023 | 18.642 | D-Galactose 6-sulfate | 0.024 | 0.108 | -0.361 |
| 571.141 | 4.297 | Unidentified | 0.006 | 0.052 | -0.362 |
| 128.107 | 3.259 | N-Cyclohexylformamide | 0.017 | 0.088 | -0.369 |
| 134.060 | 1.848 | Indoxyl | 0.005 | 0.050 | -0.381 |
| 340.089 | 4.301 | 6-Hydroxy-5-methoxyindole glucuronide | 0.004 | 0.046 | -0.383 |
| 180.087 | 6.618 | 3-Hydroxymethylglutaric acid | 0.049 | 0.163 | -0.386 |
| 642.519 | 22.062 | GlcCer(d14:2(4E,6E)/16:0) | 0.002 | 0.037 | -0.395 |
| 758.136 | 16.238 | Pelargonidin 3-sophoroside 5-glucoside | 0.004 | 0.045 | -0.397 |
| 392.371 | 22.450 | 7-dehydrocholesterol-d7 | 0.026 | 0.113 | -0.399 |
| 181.067 | 18.651 | Unidentified | 0.047 | 0.158 | -0.400 |
| 283.069 | 0.720 | 9-Riburonosylhypoxanthine | 0.004 | 0.045 | -0.402 |
| 280.231 | 1.408 | Linoleamide | 0.008 | 0.058 | -0.403 |
| 486.412 | 21.852 | Cer(d18:0/12:0) | 0.023 | 0.106 | -0.404 |
| 182.210 | 2.216 | Unidentified | 0.007 | 0.055 | -0.409 |
| 175.088 | 3.096 | Unidentified | 0.025 | 0.108 | -0.412 |
| 153.024 | 7.529 | Unidentified | 0.001 | 0.028 | -0.424 |
| 312.132 | 6.618 | N2, N2-Dimethylguanosine | 0.012 | 0.074 | -0.424 |
| 383.026 | 18.621 | Unidentified | 0.008 | 0.060 | -0.427 |
| 221.092 | 4.891 | 5-Hydroxytryptophan | 0.019 | 0.094 | -0.434 |
| 228.195 | 19.443 | Unidentified | 0.029 | 0.120 | -0.435 |
| 431.262 | 6.548 | 17-phenyl trinor PGF2α isopropyl ester | 0.020 | 0.094 | -0.436 |
| 612.187 | 1.396 | Unidentified | 0.034 | 0.131 | -0.440 |
| 582.165 | 1.068 | Unidentified | 0.039 | 0.141 | -0.451 |
| 223.076 | 4.779 | L-Cystathionine | 0.012 | 0.074 | -0.453 |
| 737.369 | 0.755 | Unidentified | 0.006 | 0.051 | -0.456 |
| 369.075 | 19.950 | 2-Deoxy-4-O-[(2E)-3-(4-hydroxyphenyl)-2-propenoyl]-3-C-(methoxycarbonyl)pentaric acid | 0.004 | 0.045 | -0.459 |
| 226.018 | 7.478 | Se-Propenylselenocysteine Se-oxide | 0.014 | 0.080 | -0.469 |
| 152.016 | 8.310 | 2-Hydroxybenzothiazole | 0.049 | 0.162 | -0.470 |
| 377.142 | 11.918 | Riboflavin | 0.015 | 0.083 | -0.473 |
| 262.214 | 1.401 | Unidentified | 0.005 | 0.050 | -0.485 |
| 175.148 | 19.447 | Unidentified | 0.032 | 0.125 | -0.485 |
| 74.060 | 1.290 | Unidentified | 0.020 | 0.095 | -0.489 |
| 90.054 | 2.340 | Alanine | 0.008 | 0.061 | -0.496 |
| 581.256 | 1.371 | Unidentified | 0.005 | 0.048 | -0.500 |
| 272.085 | 18.623 | Unidentified | 0.005 | 0.050 | -0.500 |
| 125.538 | 9.865 | Unidentified | 0.014 | 0.080 | -0.515 |
| 438.304 | 19.409 | PE(P-16:0/0:0) | 0.015 | 0.082 | -0.515 |
| 187.094 | 6.665 | Unidentified | 0.033 | 0.129 | -0.519 |
| 454.164 | 16.263 | Unidentified | 0.015 | 0.082 | -0.528 |
| 123.107 | 2.228 | Unidentified | 0.000 | 0.009 | -0.531 |
| 245.094 | 18.633 | D-Biotin | 0.021 | 0.099 | -0.534 |
| 423.500 | 16.358 | Unidentified | 0.032 | 0.125 | -0.536 |
| 499.338 | 0.750 | PG (8:0/8:0) | 0.006 | 0.051 | -0.540 |
| 281.210 | 1.404 | 12S-hydroxy-5Z,8E,10E-heptadecatrienoic acid | 0.019 | 0.094 | -0.553 |
| 634.741 | 16.356 | Cer(d18:2/23:0) | 0.010 | 0.069 | -0.554 |
| 369.215 | 0.974 | Unidentified | 0.023 | 0.106 | -0.556 |
| 492.139 | 5.771 | Unidentified | 0.004 | 0.046 | -0.570 |
| 84.043 | 4.709 | 1-Methylpyrrolinium | 0.000 | 0.009 | -0.570 |
| 148.132 | 0.975 | Unidentified | 0.004 | 0.045 | -0.575 |
| 130.064 | 15.207 | 3-Methylene-indolenine | 0.004 | 0.045 | -0.580 |
| 280.312 | 1.409 | Unidentified | 0.013 | 0.075 | -0.580 |
| 268.120 | 4.872 | Unidentified | 0.003 | 0.044 | -0.596 |
| 388.158 | 1.269 | Unidentified | 0.004 | 0.045 | -0.630 |
| 292.551 | 19.530 | Unidentified | 0.024 | 0.106 | -0.633 |
| 163.089 | 4.132 | Unidentified | 0.009 | 0.064 | -0.636 |
| 238.071 | 2.638 | Unidentified | 0.006 | 0.052 | -0.654 |
| 330.058 | 1.959 | cAMP | 0.007 | 0.053 | -0.666 |
| 997.361 | 0.762 | PIP2(16:0/18:1(11Z)) | 0.020 | 0.094 | -0.669 |
| 256.081 | 0.791 | methyl 2-[(2,3-dihydroxybenzoyl) amino]-3-hydroxypropanoate | 0.001 | 0.032 | -0.671 |
| 139.004 | 1.562 | Unidentified | 0.001 | 0.032 | -0.678 |
| 228.085 | 4.069 | L-Arogenate | 0.021 | 0.099 | -0.681 |
| 290.126 | 3.565 | Unidentified | 0.015 | 0.081 | -0.710 |
| 146.082 | 3.542 | Isobutyrylglycine | 0.030 | 0.121 | -0.731 |
| 262.321 | 1.402 | Unidentified | 0.006 | 0.051 | -0.732 |
| 314.084 | 1.945 | Unidentified | 0.000 | 0.009 | -0.770 |
| 94.065 | 4.138 | Unidentified | 0.014 | 0.080 | -0.780 |
| 247.128 | 4.380 | L-beta-aspartyl-L-leucine | 0.006 | 0.052 | -0.792 |
| 172.042 | 1.275 | Unidentified | 0.003 | 0.044 | -0.803 |
| 194.023 | 1.263 | Unidentified | 0.002 | 0.034 | -0.809 |
| 136.078 | 4.131 | L-Homocysteine | 0.022 | 0.101 | -0.814 |
| 423.194 | 1.972 | Unidentified | 0.004 | 0.046 | -0.827 |
| 192.064 | 4.132 | 5-Hydroxyindoleacetic acid | 0.007 | 0.053 | -0.863 |
| 219.097 | 1.135 | Glutamylalanine | 0.025 | 0.108 | -0.865 |
| 209.093 | 4.132 | Kynurenine | 0.013 | 0.076 | -0.879 |
| 274.090 | 1.944 | Pentose + Proline | 0.000 | 0.024 | -0.891 |
| 203.052 | 1.032 | Unidentified | 0.000 | 0.013 | -0.912 |
| 586.377 | 19.016 | Unidentified | 0.037 | 0.135 | -0.929 |
| 146.062 | 4.136 | Unidentified | 0.011 | 0.072 | -0.933 |
| 204.123 | 12.327 | Acetylcarnitine | 0.002 | 0.034 | -0.938 |
| 975.387 | 0.759 | Unidentified | 0.001 | 0.032 | -0.952 |
| 283.085 | 7.454 | Abu-Ala-OH | 0.004 | 0.045 | -0.962 |
| 235.975 | 6.961 | Unidentified | 0.003 | 0.044 | -0.981 |
| 254.099 | 8.874 | Dihydroferuloylglycine | 0.014 | 0.079 | -1.000 |
| 77.039 | 14.015 | Unidentified | 0.011 | 0.072 | -1.004 |
| 86.061 | 3.542 | Unidentified | 0.025 | 0.108 | -1.022 |
| 552.182 | 1.974 | N-Acetyl-N6, N6,O-tridemethylpuromycin-5'-phosphate | 0.001 | 0.028 | -1.027 |
| 103.054 | 14.021 | Unidentified | 0.014 | 0.080 | -1.031 |
| 206.079 | 13.455 | Indolelactic acid | 0.015 | 0.081 | -1.034 |
| 190.109 | 8.857 | Unidentified | 0.001 | 0.028 | -1.081 |
| 120.045 | 9.883 | Aminomalonic acid | 0.037 | 0.135 | -1.082 |
| 158.115 | 12.322 | Unidentified | 0.001 | 0.030 | -1.114 |
| 145.048 | 4.997 | 3-hexenedioic acid | 0.001 | 0.032 | -1.135 |
| 891.311 | 1.402 | dipalmitoyl phosphatidylinositol 3-phosphate | 0.005 | 0.050 | -1.146 |
| 208.099 | 5.024 | N-Acetyl-D-phenylalanine | 0.000 | 0.015 | -1.157 |
| 168.061 | 3.542 | Unidentified | 0.047 | 0.159 | -1.210 |
| 151.062 | 5.364 | 1-Methylhypoxanthine | 0.003 | 0.039 | -1.247 |
| 220.096 | 14.010 | Unidentified | 0.013 | 0.076 | -1.254 |
| 277.120 | 15.210 | Unidentified | 0.029 | 0.120 | -1.406 |
| 605.175 | 1.935 | SP1 | 0.000 | 0.010 | -1.418 |
| 337.021 | 14.018 | Unidentified | 0.001 | 0.032 | -1.487 |
| 120.079 | 14.016 | L-Threonine | 0.013 | 0.076 | -1.502 |
| 113.108 | 3.814 | Hydroxymethylphosphonate | 0.000 | 0.013 | -1.509 |
| 261.146 | 7.254 | L-gamma-glutamyl-L-isoleucine | 0.023 | 0.106 | -1.544 |
| 303.068 | 5.293 | Unidentified | 0.003 | 0.039 | -1.544 |
| 166.086 | 14.016 | L-Phenylalanine | 0.006 | 0.052 | -1.574 |
| 192.101 | 14.021 | Unidentified | 0.020 | 0.094 | -1.607 |
| 113.107 | 5.786 | 2-Imino-4-methylpiperidine | 0.000 | 0.009 | -1.630 |
| 334.140 | 10.201 | Unidentified | 0.001 | 0.032 | -1.735 |
| 295.129 | 8.362 | Glutamylphenylalanine | 0.012 | 0.074 | -1.856 |
| 289.120 | 8.997 | Unidentified | 0.008 | 0.057 | -1.891 |
| 260.091 | 14.014 | Unidentified | 0.010 | 0.069 | -1.904 |
| 528.120 | 14.018 | Unidentified | 0.011 | 0.070 | -2.361 |
| 185.165 | 2.408 | 10-hendecenoic acid | 0.037 | 0.135 | -3.533 |

**Table S3.** Significantly Altered Metabolites in SAECs Exposed to 5 µg/mL PM Collected from the Printer Room During Printing with PLA Filaments.

| m/z | rt | Metabolite Name | p.value | FDR | log2(FC) |
| --- | --- | --- | --- | --- | --- |
| 412.320 | 19.839 | Unidentified | 0.030 | 0.077 | 3.040 |
| 87.042 | 12.443 | 4-Deoxytetronic acid | 0.001 | 0.015 | 2.721 |
| 387.177 | 19.739 | 3,5-Di-O-methyl-8-prenylafzelechin-4beta-ol | 0.006 | 0.028 | 2.463 |
| 404.203 | 19.741 | 16-phenoxy tetranor PGF2α methyl amide | 0.009 | 0.035 | 2.322 |
| 432.238 | 19.741 | 17-phenoxy trinor PGF2α ethyl amide | 0.009 | 0.036 | 2.246 |
| 159.025 | 1.820 | fumarylacetic acid | 0.000 | 0.004 | 1.959 |
| 285.090 | 8.626 | Cysteinyl-Tyrosine | 0.005 | 0.027 | 1.913 |
| 493.198 | 2.561 | Unidentified | 0.000 | 0.009 | 1.900 |
| 137.047 | 1.820 | Hypoxanthine | 0.000 | 0.004 | 1.727 |
| 236.112 | 2.569 | N-(9-oxodecyl) acetamide | 0.000 | 0.004 | 1.636 |
| 153.041 | 2.293 | Xanthine | 0.000 | 0.004 | 1.605 |
| 186.074 | 2.569 | Unidentified | 0.000 | 0.009 | 1.604 |
| 113.034 | 2.589 | Uracil | 0.000 | 0.004 | 1.589 |
| 110.036 | 2.300 | Hypotaurine | 0.000 | 0.010 | 1.582 |
| 163.110 | 14.364 | 3-hydroxy-3-methyl-Glutaric acid | 0.006 | 0.028 | 1.562 |
| 105.071 | 19.736 | L-2,3-DIAMINOPROPIONIC ACID | 0.006 | 0.028 | 1.476 |
| 573.208 | 15.615 | Unidentified | 0.019 | 0.056 | 1.463 |
| 217.070 | 1.618 | Bisnorbiotin | 0.000 | 0.004 | 1.392 |
| 258.094 | 2.561 | Unidentified | 0.000 | 0.009 | 1.384 |
| 306.115 | 1.430 | Unidentified | 0.001 | 0.012 | 1.362 |
| 217.065 | 16.239 | Unidentified | 0.004 | 0.025 | 1.305 |
| 324.131 | 1.430 | Unidentified | 0.002 | 0.017 | 1.286 |
| 168.065 | 1.436 | Pyridoxal (Vitamin B6) | 0.012 | 0.042 | 1.142 |
| 267.056 | 2.585 | Unidentified | 0.000 | 0.009 | 1.127 |
| 460.270 | 20.183 | 17-phenyl trinor Prostaglandin E2 serinol amide | 0.001 | 0.017 | 1.113 |
| 520.332 | 14.860 | 1-Linoleoylglycerophosphocholine | 0.005 | 0.027 | 1.106 |
| 545.210 | 1.276 | Estriol 3-sulfate 16-glucuronide | 0.001 | 0.015 | 1.062 |
| 482.422 | 22.119 | Ceramide (d18:1/12:0) | 0.001 | 0.015 | 1.023 |
| 455.166 | 0.981 | Phe-Phe4Cl-OH | 0.001 | 0.016 | 1.001 |
| 205.028 | 1.227 | 3-phenyllactic acid | 0.008 | 0.032 | 0.927 |
| 119.085 | 20.192 | Succinic acid | 0.014 | 0.047 | 0.893 |
| 161.960 | 1.211 | Unidentified | 0.005 | 0.027 | 0.868 |
| 412.095 | 1.755 | Unidentified | 0.009 | 0.037 | 0.831 |
| 268.136 | 11.140 | Unidentified | 0.005 | 0.027 | 0.827 |
| 555.127 | 1.776 | 3-O-(6-O-alpha-D-Xylosylphospho-alpha-D-mannopyranosyl)-alpha-D-mannopyranose | 0.015 | 0.049 | 0.822 |
| 297.038 | 1.765 | Unidentified | 0.005 | 0.026 | 0.814 |
| 418.252 | 1.990 | 16-phenoxy Prostaglandin F2a ethyl amide | 0.020 | 0.057 | 0.812 |
| 564.362 | 15.690 | PC (18:1(9E)/2:0) | 0.016 | 0.051 | 0.803 |
| 426.079 | 1.749 | Hyaluronic acid | 0.002 | 0.019 | 0.792 |
| 301.061 | 1.799 | Unidentified | 0.012 | 0.043 | 0.787 |
| 342.297 | 20.931 | C-2 Ceramide | 0.038 | 0.088 | 0.786 |
| 284.331 | 20.203 | Unidentified | 0.038 | 0.088 | 0.777 |
| 549.343 | 1.992 | 5beta-scymnol sulfate | 0.007 | 0.030 | 0.743 |
| 94.065 | 4.138 | Unidentified | 0.018 | 0.055 | 0.725 |
| 281.135 | 20.190 | Unidentified | 0.018 | 0.055 | 0.722 |
| 541.133 | 1.751 | Unidentified | 0.014 | 0.047 | 0.700 |
| 441.044 | 1.793 | Unidentified | 0.019 | 0.056 | 0.671 |
| 464.356 | 22.202 | D-Glucosyldihydrosphingosine | 0.031 | 0.077 | 0.669 |
| 526.428 | 22.055 | Unidentified | 0.001 | 0.012 | 0.659 |
| 482.401 | 22.062 | PC(O-15:0/O-1:0) [U] | 0.002 | 0.018 | 0.659 |
| 97.970 | 2.871 | Unidentified | 0.029 | 0.074 | 0.634 |
| 309.226 | 17.938 | Unidentified | 0.004 | 0.024 | 0.621 |
| 367.266 | 18.648 | Unidentified | 0.003 | 0.023 | 0.614 |
| 724.504 | 22.768 | PC (14:1(9Z)/18:4(6Z,9Z,12Z,15Z)) | 0.023 | 0.063 | 0.614 |
| 335.108 | 12.123 | (S)-a-Amino-2,5-dihydro-5-oxo-4-isoxazolepropanoic acid N2-glucoside | 0.001 | 0.015 | 0.608 |
| 397.056 | 1.800 | Unidentified | 0.035 | 0.083 | 0.607 |
| 282.121 | 0.805 | 1-Methyladenosine | 0.007 | 0.032 | 0.604 |
| 438.372 | 22.072 | (±) N-(1-methyl-2-hydroxy-2-phenyl-ethyl) arachidonyl amine | 0.006 | 0.027 | 0.587 |
| 301.145 | 2.018 | Unidentified | 0.002 | 0.017 | 0.584 |
| 112.051 | 1.268 | Cytosine | 0.003 | 0.020 | 0.583 |
| 226.179 | 18.958 | (8S, Z)-6-((S)-3-hydroxy-2-methylpropylidene)-8-methyloctahydroindolizin-8-ol | 0.023 | 0.063 | 0.573 |
| 651.220 | 6.531 | Unidentified | 0.016 | 0.050 | 0.571 |
| 394.350 | 22.080 | N-palmitoyl histidine | 0.016 | 0.051 | 0.569 |
| 249.055 | 3.655 | Unidentified | 0.002 | 0.019 | 0.563 |
| 195.136 | 18.684 | Unidentified | 0.001 | 0.012 | 0.555 |
| 233.062 | 2.474 | Unidentified | 0.020 | 0.058 | 0.554 |
| 637.234 | 6.533 | Unidentified | 0.045 | 0.098 | 0.551 |
| 746.561 | 21.967 | PC (15:0/18:1(9Z))[U] | 0.035 | 0.083 | 0.550 |
| 426.393 | 22.298 | Vaccenyl carnitine | 0.045 | 0.098 | 0.545 |
| 238.071 | 1.920 | Unidentified | 0.026 | 0.069 | 0.541 |
| 571.098 | 1.754 | Unidentified | 0.047 | 0.100 | 0.537 |
| 154.081 | 1.974 | Dopamine | 0.005 | 0.027 | 0.520 |
| 679.234 | 5.035 | Unidentified | 0.000 | 0.009 | 0.518 |
| 534.192 | 4.296 | Unidentified | 0.000 | 0.009 | 0.516 |
| 520.199 | 4.299 | Unidentified | 0.000 | 0.009 | 0.508 |
| 452.390 | 22.438 | PC(P-14:0/0:0) | 0.023 | 0.064 | 0.507 |
| 227.173 | 7.008 | 1,8-Diazacyclotetradecane-2,9-dione | 0.007 | 0.032 | 0.502 |
| 481.036 | 1.715 | Unidentified | 0.023 | 0.064 | 0.500 |
| 401.103 | 2.205 | S-Acetylphosphopantetheine | 0.004 | 0.025 | 0.492 |
| 400.342 | 21.112 | Palmitoyl-L-carnitine | 0.002 | 0.019 | 0.490 |
| 440.369 | 21.718 | PC(O-12:0/O-1:0) | 0.002 | 0.019 | 0.489 |
| 158.155 | 19.484 | Unidentified | 0.007 | 0.030 | 0.487 |
| 247.045 | 2.470 | Glutamyl-Threonine | 0.029 | 0.075 | 0.485 |
| 570.450 | 22.046 | LysoPC(22:5(4Z,7Z,10Z,13Z,16Z)) | 0.025 | 0.066 | 0.485 |
| 70.074 | 1.961 | 1-Pyrroline | 0.002 | 0.017 | 0.484 |
| 514.410 | 21.803 | Sulfolithocholylglycine | 0.036 | 0.085 | 0.481 |
| 348.987 | 1.790 | Unidentified | 0.018 | 0.055 | 0.479 |
| 304.098 | 0.806 | Unidentified | 0.001 | 0.015 | 0.477 |
| 449.344 | 20.076 | Quercitrin | 0.045 | 0.099 | 0.475 |
| 169.035 | 1.719 | Uric acid | 0.034 | 0.081 | 0.474 |
| 454.387 | 21.954 | PC(O-12:0/O-2:0) | 0.004 | 0.025 | 0.471 |
| 448.209 | 2.006 | S-Decyl GSH | 0.005 | 0.027 | 0.469 |
| 104.106 | 0.826 | 2-Amino-3-methyl-1-butanol | 0.001 | 0.014 | 0.465 |
| 643.225 | 19.440 | Unidentified | 0.022 | 0.063 | 0.464 |
| 611.263 | 3.300 | Unidentified | 0.012 | 0.043 | 0.452 |
| 86.096 | 4.679 | 2-Methylpyrrolidine | 0.014 | 0.047 | 0.450 |
| 472.274 | 12.721 | glycolithocholic acid | 0.028 | 0.073 | 0.446 |
| 141.958 | 2.876 | Unidentified | 0.029 | 0.075 | 0.440 |
| 90.055 | 1.828 | Unidentified | 0.021 | 0.059 | 0.435 |
| 412.377 | 22.049 | PC(O-8:0/2:0) | 0.004 | 0.025 | 0.431 |
| 614.482 | 22.033 | Ferroxamine | 0.008 | 0.032 | 0.431 |
| 322.248 | 11.494 | Anandamide (18:3, n-6) | 0.011 | 0.041 | 0.430 |
| 453.339 | 14.548 | (17E)-1α,25-dihydroxy-26,27-dimethyl-17,20,22,22,23,23-hexadehydro-24a-homovitamin D3 / (17E)-1α,25-dihydroxy-26,27-dimethyl-17,20,22,22,23,23-hexadehydro-24a-homocholecalciferol | 0.011 | 0.040 | 0.423 |
| 582.174 | 2.205 | Unidentified | 0.003 | 0.020 | 0.416 |
| 486.412 | 21.852 | Cer(d18:0/12:0) | 0.011 | 0.041 | 0.411 |
| 340.260 | 11.485 | Unidentified | 0.002 | 0.019 | 0.410 |
| 267.005 | 1.782 | Unidentified | 0.038 | 0.088 | 0.409 |
| 685.284 | 4.297 | gamma-L-Glutamyl-butirosin B | 0.004 | 0.025 | 0.408 |
| 317.118 | 2.000 | Unidentified | 0.003 | 0.023 | 0.404 |
| 528.464 | 22.286 | LysoPE(0:0/22:5(4Z,7Z,10Z,13Z,16Z)) | 0.036 | 0.085 | 0.395 |
| 369.110 | 4.301 | Unidentified | 0.000 | 0.009 | 0.394 |
| 264.109 | 0.803 | Unidentified | 0.028 | 0.073 | 0.387 |
| 320.135 | 6.755 | Unidentified | 0.002 | 0.017 | 0.375 |
| 198.070 | 7.524 | Unidentified | 0.005 | 0.027 | 0.369 |
| 548.468 | 21.597 | PC(O-3:1(1E)/O-18:1(9Z)) [S] | 0.020 | 0.057 | 0.369 |
| 96.960 | 4.669 | Methaneselenol | 0.005 | 0.027 | 0.367 |
| 693.216 | 5.019 | Unidentified | 0.016 | 0.050 | 0.355 |
| 118.087 | 1.095 | L-Valine | 0.007 | 0.032 | 0.350 |
| 347.092 | 0.826 | Unidentified | 0.039 | 0.089 | 0.345 |
| 384.343 | 22.737 | N-stearoyl valine | 0.024 | 0.065 | 0.344 |
| 424.162 | 16.360 | Unidentified | 0.037 | 0.088 | 0.343 |
| 554.462 | 22.070 | Cer(d14:1(4E)/20:0(2OH)) | 0.003 | 0.021 | 0.343 |
| 539.149 | 1.737 | Unidentified | 0.029 | 0.075 | 0.342 |
| 221.092 | 6.552 | 5-Hydroxy-L-tryptophan | 0.004 | 0.025 | 0.340 |
| 115.956 | 1.211 | 3-keto-2-methylbutyrate | 0.011 | 0.040 | 0.339 |
| 572.156 | 1.395 | Unidentified | 0.040 | 0.091 | 0.338 |
| 72.082 | 1.092 | Unidentified | 0.011 | 0.041 | 0.331 |
| 197.010 | 18.642 | Allantoin | 0.034 | 0.081 | 0.330 |
| 384.299 | 18.639 | Unidentified | 0.015 | 0.048 | 0.326 |
| 447.199 | 1.986 | Unidentified | 0.027 | 0.070 | 0.325 |
| 210.044 | 7.521 | Unidentified | 0.033 | 0.080 | 0.324 |
| 157.044 | 7.526 | Unidentified | 0.001 | 0.013 | 0.324 |
| 442.388 | 21.800 | Leukotriene E3 | 0.002 | 0.017 | 0.323 |
| 540.451 | 22.395 | Hexacosanoyl carnitine | 0.022 | 0.063 | 0.322 |
| 510.432 | 22.060 | PC(O-10:0/O-8:0) [U] | 0.009 | 0.035 | 0.318 |
| 512.430 | 21.816 | PS(O-18:0/0:0) | 0.024 | 0.064 | 0.301 |
| 548.170 | 2.168 | Unidentified | 0.033 | 0.080 | 0.291 |
| 227.100 | 1.878 | Unidentified | 0.017 | 0.052 | 0.284 |
| 551.256 | 3.552 | Unidentified | 0.009 | 0.036 | 0.283 |
| 408.370 | 21.730 | 7alpha-hydroxy-4-cholesten-3-one-d7 | 0.033 | 0.081 | 0.277 |
| 160.034 | 0.845 | Unidentified | 0.029 | 0.075 | 0.266 |
| 458.382 | 21.709 | Arachidyl carnitine | 0.048 | 0.103 | 0.253 |
| 362.241 | 11.485 | 3-Oxohexadecanoic acid glycerides | 0.031 | 0.077 | 0.253 |
| 428.373 | 21.645 | C8-Dihydroceramide | 0.002 | 0.017 | 0.248 |
| 470.187 | 1.995 | Unidentified | 0.026 | 0.069 | 0.238 |
| 116.071 | 1.672 | L-Proline | 0.007 | 0.032 | 0.235 |
| 225.088 | 0.801 | 3-Hydroxy-DL-kynurenine | 0.023 | 0.064 | 0.234 |
| 356.187 | 1.068 | PC(O-4:0/2:0) [U] | 0.034 | 0.082 | 0.230 |
| 378.356 | 22.069 | N-arachidonoyl dihydroxypropylamine | 0.025 | 0.067 | 0.220 |
| 431.262 | 6.548 | 17-phenyl trinor PGF2α isopropyl ester | 0.004 | 0.024 | 0.220 |
| 410.105 | 1.745 | 1-(5-Phosphoribosyl)-4-(N-succinocarboxamide)-5-aminoimidazole | 0.041 | 0.092 | 0.213 |
| 100.075 | 1.069 | N-Methyl-2-pyrrolidinone | 0.049 | 0.104 | 0.208 |
| 331.166 | 4.297 | 8-Hydroxyclomipramine | 0.005 | 0.027 | 0.205 |
| 132.102 | 2.014 | L-Isoleucine | 0.006 | 0.029 | 0.200 |
| 136.113 | 23.292 | Unidentified | 0.016 | 0.049 | 0.195 |
| 194.119 | 3.935 | Unidentified | 0.021 | 0.061 | 0.190 |
| 133.030 | 1.785 | Unidentified | 0.019 | 0.056 | 0.190 |
| 103.054 | 6.539 | Unidentified | 0.005 | 0.027 | 0.183 |
| 366.094 | 5.041 | 2-S-Glutathionyl acetate | 0.024 | 0.065 | 0.175 |
| 584.185 | 1.227 | Unidentified | 0.045 | 0.099 | 0.175 |
| 134.998 | 1.192 | Malic acid | 0.001 | 0.013 | 0.167 |
| 114.046 | 6.538 | Unidentified | 0.005 | 0.027 | 0.167 |
| 118.066 | 4.301 | Unidentified | 0.019 | 0.056 | 0.162 |
| 101.040 | 4.299 | Succinic anhydride | 0.049 | 0.104 | 0.158 |
| 105.047 | 4.298 | Malonic acid | 0.000 | 0.004 | 0.157 |
| 152.055 | 1.279 | Guanine | 0.005 | 0.027 | 0.155 |
| 175.120 | 1.047 | L-Arginine | 0.023 | 0.064 | 0.152 |
| 86.504 | 1.989 | L-Homocysteine sulfonic acid | 0.013 | 0.045 | 0.141 |
| 150.058 | 1.281 | L-Methionine | 0.008 | 0.034 | 0.132 |
| 552.531 | 23.282 | Cer(d18:1/17:0) | 0.007 | 0.030 | 0.132 |
| 149.061 | 4.298 | Unidentified | 0.000 | 0.004 | 0.128 |
| 93.072 | 4.298 | Unidentified | 0.013 | 0.045 | 0.125 |
| 148.079 | 4.298 | Unidentified | 0.005 | 0.027 | 0.121 |
| 100.037 | 15.369 | Succinimide | 0.006 | 0.030 | 0.121 |
| 107.052 | 4.299 | Unidentified | 0.002 | 0.019 | 0.114 |
| 103.055 | 2.223 | Unidentified | 0.001 | 0.017 | 0.110 |
| 119.075 | 4.299 | Methyl-3-hydroxybutyric acid | 0.002 | 0.019 | 0.099 |
| 131.048 | 4.298 | Unidentified | 0.003 | 0.020 | 0.097 |
| 104.054 | 1.283 | Biuret | 0.019 | 0.056 | 0.093 |
| 102.056 | 1.715 | Unidentified | 0.027 | 0.071 | 0.087 |
| 166.204 | 4.293 | Unidentified | 0.033 | 0.081 | 0.035 |
| 302.119 | 1.350 | Unidentified | 0.044 | 0.098 | -0.041 |
| 264.121 | 5.021 | 7-Mercaptoheptanoylthreonine | 0.012 | 0.042 | -0.083 |
| 310.126 | 5.021 | Unidentified | 0.038 | 0.088 | -0.091 |
| 107.047 | 5.018 | Unidentified | 0.005 | 0.027 | -0.094 |
| 292.119 | 5.037 | Unidentified | 0.048 | 0.102 | -0.104 |
| 105.071 | 5.021 | Unidentified | 0.014 | 0.047 | -0.111 |
| 194.080 | 2.701 | Phenylacetylglycine | 0.021 | 0.061 | -0.112 |
| 344.135 | 2.720 | b-D-Glucopyranosiduronic acid | 0.050 | 0.105 | -0.114 |
| 246.112 | 5.034 | Asparaginyl-Hydroxyproline | 0.035 | 0.083 | -0.119 |
| 97.028 | 5.033 | Methaneselenol | 0.022 | 0.061 | -0.137 |
| 354.167 | 0.838 | Lys-Val-OH | 0.014 | 0.047 | -0.140 |
| 343.027 | 0.856 | Unidentified | 0.041 | 0.092 | -0.142 |
| 260.113 | 1.069 | Unidentified | 0.033 | 0.080 | -0.158 |
| 280.115 | 2.727 | Unidentified | 0.034 | 0.081 | -0.159 |
| 601.108 | 0.825 | Protoporphyrin IX | 0.003 | 0.023 | -0.161 |
| 145.106 | 0.953 | 4-Guanidinobutanamide | 0.019 | 0.056 | -0.163 |
| 398.987 | 0.896 | Unidentified | 0.007 | 0.031 | -0.163 |
| 276.120 | 6.746 | Norophthalmic acid | 0.018 | 0.054 | -0.165 |
| 130.202 | 1.696 | Octylamine | 0.006 | 0.027 | -0.167 |
| 280.139 | 1.403 | Unidentified | 0.030 | 0.076 | -0.168 |
| 114.091 | 7.095 | Unidentified | 0.003 | 0.023 | -0.179 |
| 310.224 | 5.016 | Unidentified | 0.016 | 0.049 | -0.183 |
| 331.127 | 6.761 | Unidentified | 0.048 | 0.102 | -0.188 |
| 516.224 | 1.070 | Unidentified | 0.005 | 0.026 | -0.191 |
| 161.068 | 1.405 | Unidentified | 0.022 | 0.061 | -0.196 |
| 189.071 | 16.249 | Unidentified | 0.012 | 0.042 | -0.196 |
| 152.033 | 1.700 | Unidentified | 0.008 | 0.032 | -0.197 |
| 582.231 | 2.702 | Unidentified | 0.025 | 0.067 | -0.198 |
| 349.136 | 6.750 | Unidentified | 0.020 | 0.058 | -0.201 |
| 367.152 | 6.750 | Unidentified | 0.019 | 0.056 | -0.202 |
| 481.172 | 16.229 | Tyr-TyrMe-OH | 0.006 | 0.027 | -0.202 |
| 603.102 | 0.831 | Unidentified | 0.008 | 0.034 | -0.206 |
| 142.086 | 1.408 | Unidentified | 0.014 | 0.046 | -0.209 |
| 308.114 | 2.737 | Cysteinyl-Tryptophan | 0.017 | 0.052 | -0.211 |
| 297.056 | 2.997 | Unidentified | 0.043 | 0.096 | -0.212 |
| 227.080 | 6.544 | TRYPTOPHAN | 0.039 | 0.089 | -0.212 |
| 220.045 | 16.248 | 4-Amino-2-methyl-5-phosphomethylpyrimidine | 0.002 | 0.020 | -0.213 |
| 571.141 | 4.297 | Unidentified | 0.027 | 0.070 | -0.216 |
| 100.041 | 16.231 | Unidentified | 0.033 | 0.081 | -0.230 |
| 143.056 | 1.404 | Unidentified | 0.005 | 0.027 | -0.232 |
| 393.071 | 0.963 | 5,7,3',4',5'-Pentahydroxy-3,6,8-trimethoxyflavone | 0.004 | 0.025 | -0.233 |
| 328.427 | 5.010 | Stearoylethanolamide | 0.039 | 0.089 | -0.235 |
| 262.130 | 1.401 | Unidentified | 0.010 | 0.038 | -0.235 |
| 216.124 | 1.399 | Unidentified | 0.017 | 0.053 | -0.242 |
| 198.113 | 1.405 | Metanephrine | 0.004 | 0.025 | -0.244 |
| 162.078 | 6.751 | L-2-Aminoadipic acid | 0.014 | 0.047 | -0.248 |
| 123.107 | 2.228 | Unidentified | 0.027 | 0.072 | -0.250 |
| 473.177 | 2.162 | C1'-C9-Glycosylated UWM6 | 0.032 | 0.079 | -0.251 |
| 187.094 | 6.665 | Unidentified | 0.026 | 0.069 | -0.262 |
| 388.158 | 1.269 | Unidentified | 0.040 | 0.090 | -0.273 |
| 362.037 | 16.238 | Unidentified | 0.030 | 0.076 | -0.277 |
| 273.089 | 18.650 | Unidentified | 0.015 | 0.048 | -0.278 |
| 312.132 | 6.618 | N2, N2-Dimethylguanosine | 0.036 | 0.085 | -0.283 |
| 458.692 | 17.498 | 7-methylguanosine 5'-diphosphate | 0.016 | 0.050 | -0.284 |
| 375.995 | 1.654 | Unidentified | 0.049 | 0.103 | -0.284 |
| 220.081 | 6.735 | O-Succinyl-L-homoserine | 0.046 | 0.099 | -0.284 |
| 316.078 | 1.070 | Unidentified | 0.020 | 0.058 | -0.286 |
| 326.119 | 7.458 | Citalopram (propionic acid derivative) | 0.031 | 0.077 | -0.286 |
| 603.204 | 0.962 | Unidentified | 0.019 | 0.056 | -0.286 |
| 203.052 | 1.032 | Unidentified | 0.002 | 0.018 | -0.290 |
| 268.120 | 4.872 | Unidentified | 0.006 | 0.027 | -0.299 |
| 80.048 | 1.845 | Unidentified | 0.010 | 0.038 | -0.305 |
| 229.095 | 6.755 | Unidentified | 0.003 | 0.023 | -0.313 |
| 375.097 | 16.253 | Unidentified | 0.003 | 0.022 | -0.314 |
| 284.058 | 16.265 | N2-Acetyl-L-aminoadipyl-δ-phosphate | 0.008 | 0.034 | -0.315 |
| 114.036 | 18.678 | Unidentified | 0.007 | 0.031 | -0.315 |
| 162.039 | 18.676 | N-Formyl-L-aspartate | 0.015 | 0.048 | -0.315 |
| 350.119 | 20.060 | Unidentified | 0.005 | 0.027 | -0.317 |
| 272.085 | 18.623 | Unidentified | 0.036 | 0.085 | -0.318 |
| 803.234 | 0.783 | Unidentified | 0.035 | 0.083 | -0.325 |
| 311.229 | 5.014 | 9-hydroperoxy-10E,12,15Z-octadecatrienoic acid | 0.008 | 0.032 | -0.328 |
| 581.256 | 1.371 | Unidentified | 0.027 | 0.071 | -0.332 |
| 181.067 | 18.651 | Unidentified | 0.000 | 0.009 | -0.332 |
| 300.103 | 1.069 | 8-hydroxy Guanosine | 0.002 | 0.017 | -0.339 |
| 168.079 | 6.753 | N-Trimethyl-2-aminoethylphosphonate | 0.005 | 0.026 | -0.341 |
| 84.043 | 4.709 | 1-Methylpyrrolinium | 0.001 | 0.015 | -0.342 |
| 218.065 | 6.752 | 2-(Hydroxymethyl)-3-(acetamidomethylene)succinate | 0.005 | 0.027 | -0.344 |
| 136.077 | 16.229 | N-Acetylarylamine | 0.002 | 0.017 | -0.345 |
| 280.231 | 1.408 | Linoleamide | 0.011 | 0.041 | -0.345 |
| 177.102 | 16.232 | Unidentified | 0.013 | 0.044 | -0.351 |
| 331.001 | 1.670 | Unidentified | 0.033 | 0.081 | -0.354 |
| 322.087 | 1.067 | Deoxy-5-methylcytidylate | 0.004 | 0.026 | -0.355 |
| 167.094 | 1.133 | N-Formyl-4-amino-5-aminomethyl-2-methylpyrimidine | 0.006 | 0.028 | -0.357 |
| 310.315 | 5.011 | Unidentified | 0.038 | 0.088 | -0.357 |
| 238.071 | 2.638 | Unidentified | 0.014 | 0.047 | -0.361 |
| 384.022 | 18.618 | Unidentified | 0.033 | 0.080 | -0.363 |
| 308.169 | 5.486 | Unidentified | 0.022 | 0.061 | -0.363 |
| 383.026 | 18.621 | Unidentified | 0.015 | 0.048 | -0.365 |
| 275.138 | 16.232 | Glutaminyl-Glutamine | 0.003 | 0.023 | -0.370 |
| 134.060 | 1.848 | Indoxyl | 0.001 | 0.011 | -0.376 |
| 91.055 | 5.019 | Unidentified | 0.003 | 0.023 | -0.380 |
| 569.208 | 0.785 | Leukotriene F4 | 0.000 | 0.010 | -0.381 |
| 483.040 | 1.184 | Thymidine 5'-triphosphate | 0.007 | 0.030 | -0.382 |
| 106.048 | 1.282 | L-Serine | 0.004 | 0.026 | -0.383 |
| 263.122 | 16.226 | Nopalinic acid | 0.002 | 0.019 | -0.385 |
| 128.053 | 16.230 | Unidentified | 0.004 | 0.025 | -0.387 |
| 164.052 | 16.226 | 4-Hydroxy-L-glutamic acid | 0.000 | 0.010 | -0.388 |
| 174.056 | 16.229 | Quinaldic acid | 0.003 | 0.022 | -0.395 |
| 191.082 | 16.230 | 2-Oxo-7-methylthioheptanoic acid | 0.000 | 0.009 | -0.395 |
| 172.042 | 1.275 | Unidentified | 0.011 | 0.040 | -0.397 |
| 96.080 | 1.849 | Unidentified | 0.005 | 0.027 | -0.398 |
| 669.206 | 20.541 | Unidentified | 0.016 | 0.050 | -0.402 |
| 228.085 | 4.069 | L-Arogenate | 0.002 | 0.019 | -0.403 |
| 408.181 | 1.850 | Unidentified | 0.025 | 0.067 | -0.405 |
| 518.237 | 1.366 | PS(18:4(6Z,9Z,12Z,15Z)/0:0) | 0.002 | 0.019 | -0.409 |
| 355.066 | 18.653 | Phenolsulfonphthalein | 0.002 | 0.019 | -0.416 |
| 535.535 | 18.184 | 1-(8E-octadecenyl)-heptadecanoate | 0.017 | 0.051 | -0.420 |
| 170.079 | 1.848 | Pyridoxine (Vitamin B6) | 0.004 | 0.025 | -0.422 |
| 146.082 | 3.542 | Isobutyrylglycine | 0.046 | 0.099 | -0.422 |
| 515.211 | 3.547 | Unidentified | 0.003 | 0.020 | -0.423 |
| 538.202 | 1.069 | Unidentified | 0.011 | 0.041 | -0.427 |
| 454.164 | 16.263 | Unidentified | 0.039 | 0.089 | -0.428 |
| 633.078 | 1.673 | Unidentified | 0.010 | 0.037 | -0.433 |
| 736.214 | 4.295 | Unidentified | 0.025 | 0.066 | -0.437 |
| 203.082 | 16.242 | Unidentified | 0.005 | 0.027 | -0.448 |
| 543.154 | 0.745 | Unidentified | 0.000 | 0.009 | -0.450 |
| 274.107 | 12.258 | L-Thyronine | 0.001 | 0.015 | -0.450 |
| 212.002 | 1.154 | Se-Methylselenomethionine | 0.018 | 0.055 | -0.454 |
| 371.144 | 4.847 | 4beta-Hydroxyobovatachromene | 0.004 | 0.025 | -0.457 |
| 309.128 | 0.790 | Unidentified | 0.008 | 0.034 | -0.459 |
| 256.081 | 0.791 | methyl 2-[(2,3-dihydroxybenzoyl) amino]-3-hydroxypropanoate | 0.016 | 0.050 | -0.468 |
| 367.225 | 6.741 | 1,2-dihydroxyheptadec-16-en-4-yl acetate | 0.008 | 0.032 | -0.468 |
| 353.113 | 16.244 | Penicilloic G acid | 0.002 | 0.019 | -0.477 |
| 535.198 | 18.189 | 5''-Phosphoribostamycin | 0.000 | 0.005 | -0.477 |
| 291.099 | 4.839 | N2-Malonyl-D-tryptophan | 0.021 | 0.061 | -0.482 |
| 330.058 | 1.959 | cAMP | 0.004 | 0.024 | -0.482 |
| 505.037 | 1.662 | Thiamin triphosphate | 0.003 | 0.023 | -0.483 |
| 124.074 | 5.778 | Unidentified | 0.003 | 0.020 | -0.485 |
| 261.023 | 18.642 | D-Galactose 6-sulfate | 0.001 | 0.015 | -0.486 |
| 194.023 | 1.263 | Unidentified | 0.006 | 0.027 | -0.489 |
| 153.075 | 1.844 | Ribitol | 0.001 | 0.015 | -0.492 |
| 410.140 | 16.029 | Unidentified | 0.012 | 0.042 | -0.494 |
| 781.252 | 0.776 | N, N'-Diacetylchitobiosyldiphosphodolichol | 0.005 | 0.027 | -0.495 |
| 152.016 | 8.310 | 2-Hydroxybenzothiazole | 0.045 | 0.098 | -0.498 |
| 102.057 | 9.879 | Unidentified | 0.006 | 0.027 | -0.500 |
| 148.132 | 0.975 | Unidentified | 0.027 | 0.071 | -0.500 |
| 369.215 | 0.974 | Unidentified | 0.029 | 0.075 | -0.505 |
| 354.147 | 11.344 | Unidentified | 0.032 | 0.079 | -0.507 |
| 389.132 | 6.749 | TyrMe-Ala-OH | 0.018 | 0.055 | -0.514 |
| 175.148 | 19.447 | Unidentified | 0.042 | 0.094 | -0.515 |
| 152.038 | 0.774 | Unidentified | 0.033 | 0.080 | -0.516 |
| 617.243 | 16.215 | Unidentified | 0.001 | 0.013 | -0.517 |
| 344.238 | 2.705 | Unidentified | 0.041 | 0.092 | -0.520 |
| 133.964 | 1.201 | Unidentified | 0.002 | 0.019 | -0.523 |
| 423.194 | 1.972 | Unidentified | 0.006 | 0.027 | -0.530 |
| 342.086 | 16.069 | Unidentified | 0.031 | 0.077 | -0.536 |
| 192.054 | 9.894 | Unidentified | 0.016 | 0.049 | -0.538 |
| 367.318 | 6.744 | Unidentified | 0.023 | 0.064 | -0.539 |
| 534.863 | 18.177 | LysoPE(0:0/22:2(13Z,16Z)) | 0.018 | 0.054 | -0.539 |
| 258.111 | 1.797 | Glycerophosphocholine | 0.019 | 0.056 | -0.544 |
| 582.165 | 1.068 | Unidentified | 0.003 | 0.022 | -0.568 |
| 337.175 | 0.774 | Unidentified | 0.039 | 0.089 | -0.574 |
| 143.119 | 0.767 | Unidentified | 0.004 | 0.025 | -0.574 |
| 402.246 | 19.772 | Unidentified | 0.040 | 0.090 | -0.575 |
| 714.235 | 4.297 | PS (13:0/18:4(6Z,9Z,12Z,15Z)) | 0.047 | 0.101 | -0.588 |
| 148.053 | 9.879 | L-Glutamate | 0.001 | 0.015 | -0.591 |
| 438.304 | 19.409 | PE(P-16:0/0:0) | 0.002 | 0.019 | -0.594 |
| 226.018 | 7.478 | Se-Propenylselenocysteine Se-oxide | 0.008 | 0.032 | -0.596 |
| 356.075 | 1.826 | Asn-Asp-OH | 0.001 | 0.015 | -0.599 |
| 280.312 | 1.409 | Unidentified | 0.011 | 0.041 | -0.599 |
| 281.210 | 1.404 | 12S-hydroxy-5Z,8E,10E-heptadecatrienoic acid | 0.013 | 0.045 | -0.604 |
| 184.098 | 5.776 | 3-phenyllactic acid | 0.003 | 0.021 | -0.606 |
| 130.048 | 9.874 | Pyroglutamic acid | 0.001 | 0.013 | -0.609 |
| 274.090 | 1.944 | Pentose + Proline | 0.001 | 0.012 | -0.613 |
| 241.030 | 14.023 | L-Cystine | 0.045 | 0.098 | -0.618 |
| 290.126 | 3.565 | Unidentified | 0.000 | 0.009 | -0.619 |
| 86.061 | 3.542 | Unidentified | 0.046 | 0.099 | -0.620 |
| 423.500 | 16.358 | Unidentified | 0.009 | 0.037 | -0.628 |
| 247.128 | 4.380 | L-beta-aspartyl-L-leucine | 0.003 | 0.023 | -0.635 |
| 353.217 | 16.244 | 2-Pyrrolidinone, 1-ethyl-4-[2-[(2-hydroxyethyl) amino]ethyl]-3,3-diphenyl- (7CI,8CI,9CI) | 0.004 | 0.025 | -0.639 |
| 214.106 | 1.069 | Unidentified | 0.007 | 0.030 | -0.640 |
| 547.230 | 0.780 | Unidentified | 0.013 | 0.044 | -0.643 |
| 160.043 | 14.016 | Unidentified | 0.050 | 0.105 | -0.651 |
| 326.219 | 2.700 | Farnesylcysteine | 0.010 | 0.039 | -0.664 |
| 221.066 | 16.053 | Unidentified | 0.013 | 0.045 | -0.669 |
| 283.085 | 7.454 | Abu-Ala-OH | 0.001 | 0.017 | -0.675 |
| 670.398 | 18.996 | PE (13:0/18:4(6Z,9Z,12Z,15Z)) | 0.032 | 0.079 | -0.676 |
| 786.205 | 6.741 | Flavin adenine dinucleotide (FAD) | 0.007 | 0.030 | -0.682 |
| 577.221 | 1.068 | Formononetin 7-O-rutinoside | 0.000 | 0.009 | -0.688 |
| 758.136 | 16.238 | Pelargonidin 3-sophoroside 5-glucoside | 0.000 | 0.004 | -0.690 |
| 197.102 | 2.896 | L-(+)-Arginine | 0.014 | 0.046 | -0.695 |
| 314.084 | 1.945 | Unidentified | 0.000 | 0.004 | -0.699 |
| 176.055 | 9.874 | N-Formyl-L-glutamate | 0.000 | 0.004 | -0.712 |
| 220.116 | 5.776 | Pantothenic Acid | 0.015 | 0.048 | -0.715 |
| 407.157 | 0.766 | Unidentified | 0.007 | 0.030 | -0.724 |
| 237.000 | 1.613 | Unidentified | 0.008 | 0.032 | -0.737 |
| 90.054 | 2.340 | Alanine | 0.004 | 0.024 | -0.746 |
| 283.069 | 0.720 | 9-Riburonosylhypoxanthine | 0.032 | 0.079 | -0.747 |
| 202.108 | 1.394 | (E)-1-(4-methylquinazolin-2(1H)-ylidene) guanidine | 0.011 | 0.040 | -0.748 |
| 262.321 | 1.402 | Unidentified | 0.005 | 0.027 | -0.756 |
| 354.075 | 4.780 | Unidentified | 0.006 | 0.029 | -0.769 |
| 369.075 | 19.950 | 2-Deoxy-4-O-[(2E)-3-(4-hydroxyphenyl)-2-propenoyl]-3-C-(methoxycarbonyl)pentaric acid | 0.000 | 0.004 | -0.774 |
| 106.041 | 9.874 | 2-cyano-Pyrimidine | 0.000 | 0.009 | -0.788 |
| 552.182 | 1.974 | N-Acetyl-N6, N6, O-tridemethylpuromycin-5'-phosphate | 0.001 | 0.013 | -0.790 |
| 440.151 | 15.704 | Unidentified | 0.015 | 0.049 | -0.795 |
| 319.035 | 5.778 | Unidentified | 0.009 | 0.037 | -0.810 |
| 542.357 | 18.984 | PC (20:5(5Z,8Z,11Z,14Z,17Z)/0:0) | 0.030 | 0.077 | -0.815 |
| 634.741 | 16.356 | Cer(d18:2/23:0) | 0.003 | 0.023 | -0.817 |
| 313.105 | 9.874 | Thr-Ala-OH | 0.003 | 0.022 | -0.828 |
| 642.519 | 22.062 | GlcCer(d14:2(4E,6E)/16:0) | 0.002 | 0.019 | -0.829 |
| 262.214 | 1.401 | Unidentified | 0.001 | 0.012 | -0.829 |
| 152.118 | 1.842 | Unidentified | 0.002 | 0.019 | -0.835 |
| 279.059 | 0.769 | Unidentified | 0.002 | 0.019 | -0.842 |
| 454.113 | 9.043 | Unidentified | 0.012 | 0.042 | -0.851 |
| 130.064 | 15.207 | 3-Methylene-indolenine | 0.001 | 0.013 | -0.852 |
| 223.076 | 4.779 | L-Cystathionine | 0.001 | 0.017 | -0.862 |
| 231.114 | 15.199 | Unidentified | 0.004 | 0.025 | -0.879 |
| 295.093 | 9.876 | 4-(o-Carboxybenzamido)glutaramic acid | 0.000 | 0.004 | -0.881 |
| 521.170 | 0.772 | Unidentified | 0.004 | 0.024 | -0.882 |
| 661.236 | 16.249 | Unidentified | 0.001 | 0.015 | -0.889 |
| 125.538 | 9.865 | Unidentified | 0.004 | 0.025 | -0.892 |
| 277.064 | 0.770 | Unidentified | 0.006 | 0.028 | -0.894 |
| 515.172 | 15.768 | TyrMe-Nap-OH | 0.004 | 0.026 | -0.928 |
| 267.096 | 9.892 | Unidentified | 0.002 | 0.019 | -0.953 |
| 891.311 | 1.402 | dipalmitoyl phosphatidylinositol 3-phosphate | 0.010 | 0.040 | -0.954 |
| 267.146 | 3.039 | Unidentified | 0.013 | 0.045 | -0.956 |
| 190.109 | 8.857 | Unidentified | 0.003 | 0.022 | -0.963 |
| 157.136 | 0.772 | β-nonylenic acid | 0.005 | 0.027 | -0.966 |
| 353.315 | 16.232 | PGE2 | 0.004 | 0.025 | -0.969 |
| 261.086 | 2.163 | Unidentified | 0.002 | 0.019 | -0.973 |
| 113.107 | 5.786 | 2-Imino-4-methylpiperidine | 0.000 | 0.009 | -1.024 |
| 242.099 | 5.776 | Unidentified | 0.012 | 0.042 | -1.028 |
| 113.108 | 3.814 | Hydroxymethylphosphonate | 0.000 | 0.004 | -1.044 |
| 759.271 | 0.771 | Unidentified | 0.002 | 0.017 | -1.055 |
| 442.148 | 9.874 | Folic acid | 0.000 | 0.002 | -1.059 |
| 151.062 | 5.364 | 1-Methylhypoxanthine | 0.001 | 0.012 | -1.081 |
| 303.068 | 5.293 | Unidentified | 0.000 | 0.009 | -1.089 |
| 120.045 | 9.883 | Aminomalonic acid | 0.021 | 0.061 | -1.094 |
| 674.429 | 19.078 | Unidentified | 0.007 | 0.030 | -1.096 |
| 145.048 | 4.997 | 3-hexenedioic acid | 0.001 | 0.017 | -1.101 |
| 687.285 | 19.381 | Unidentified | 0.005 | 0.026 | -1.106 |
| 168.061 | 3.542 | Unidentified | 0.006 | 0.029 | -1.107 |
| 413.220 | 0.764 | Unidentified | 0.010 | 0.040 | -1.112 |
| 277.120 | 12.502 | Unidentified | 0.004 | 0.026 | -1.115 |
| 355.118 | 18.623 | Unidentified | 0.008 | 0.032 | -1.121 |
| 461.212 | 5.776 | Unidentified | 0.014 | 0.047 | -1.123 |
| 558.349 | 18.655 | Unidentified | 0.041 | 0.092 | -1.159 |
| 381.147 | 1.646 | 2-Methoxyestrone 3-sulfate | 0.004 | 0.025 | -1.165 |
| 515.165 | 0.769 | Unidentified | 0.005 | 0.027 | -1.173 |
| 163.089 | 4.132 | Unidentified | 0.001 | 0.013 | -1.183 |
| 232.118 | 1.068 | Suberylglycine | 0.030 | 0.076 | -1.212 |
| 605.175 | 1.935 | SP1 | 0.000 | 0.009 | -1.212 |
| 477.208 | 0.768 | Unidentified | 0.033 | 0.080 | -1.236 |
| 715.307 | 0.769 | Unidentified | 0.044 | 0.097 | -1.241 |
| 394.176 | 0.765 | Unidentified | 0.010 | 0.037 | -1.259 |
| 235.975 | 6.961 | Unidentified | 0.006 | 0.028 | -1.262 |
| 705.228 | 16.255 | Unidentified | 0.000 | 0.009 | -1.264 |
| 499.189 | 0.769 | Unidentified | 0.005 | 0.026 | -1.272 |
| 204.123 | 12.327 | Acetylcarnitine | 0.000 | 0.009 | -1.281 |
| 492.139 | 5.771 | Unidentified | 0.018 | 0.055 | -1.328 |
| 146.062 | 4.136 | Unidentified | 0.002 | 0.019 | -1.351 |
| 158.115 | 12.322 | Unidentified | 0.000 | 0.009 | -1.405 |
| 263.094 | 5.756 | Unidentified | 0.002 | 0.017 | -1.407 |
| 136.078 | 4.131 | L-Homocysteine | 0.002 | 0.019 | -1.415 |
| 192.064 | 4.132 | 5-Hydroxyindoleacetic acid | 0.001 | 0.015 | -1.431 |
| 737.284 | 0.767 | Unidentified | 0.015 | 0.048 | -1.439 |
| 748.279 | 0.759 | PE (15:1(9Z)/22:6(4Z,7Z,10Z,13Z,16Z,19Z)) | 0.001 | 0.017 | -1.497 |
| 334.140 | 10.201 | Unidentified | 0.002 | 0.019 | -1.519 |
| 261.146 | 7.254 | L-gamma-glutamyl-L-isoleucine | 0.000 | 0.010 | -1.540 |
| 753.262 | 0.765 | Unidentified | 0.006 | 0.027 | -1.556 |
| 77.039 | 14.015 | Unidentified | 0.001 | 0.015 | -1.619 |
| 997.361 | 0.762 | PIP2(16:0/18:1(11Z)) | 0.011 | 0.040 | -1.643 |
| 279.097 | 4.456 | gamma-Glutamylmethionine | 0.016 | 0.050 | -1.649 |
| 977.603 | 17.844 | Unidentified | 0.037 | 0.088 | -1.668 |
| 209.093 | 4.132 | Kynurenine | 0.002 | 0.017 | -1.683 |
| 206.079 | 13.455 | Indolelactic acid | 0.010 | 0.038 | -1.704 |
| 261.215 | 0.748 | Unidentified | 0.004 | 0.025 | -1.720 |
| 103.054 | 14.021 | Unidentified | 0.001 | 0.017 | -1.735 |
| 441.152 | 5.224 | (+)-12a-Hydroxyerythynone | 0.006 | 0.028 | -1.742 |
| 254.099 | 8.874 | Dihydroferuloylglycine | 0.005 | 0.027 | -1.760 |
| 575.129 | 14.016 | Unidentified | 0.006 | 0.030 | -1.780 |
| 337.021 | 14.018 | Unidentified | 0.000 | 0.005 | -1.826 |
| 208.099 | 5.024 | N-Acetyl-D-phenylalanine | 0.000 | 0.009 | -1.836 |
| 295.129 | 8.362 | Glutamylphenylalanine | 0.008 | 0.032 | -1.971 |
| 220.096 | 14.010 | Unidentified | 0.000 | 0.009 | -2.015 |
| 968.448 | 17.840 | Trihexosylceramide (d18:1/12:0) | 0.044 | 0.097 | -2.100 |
| 968.782 | 17.841 | TG (20:0/20:1(11Z)/20:0) (d5) | 0.042 | 0.094 | -2.154 |
| 277.120 | 15.210 | Unidentified | 0.000 | 0.009 | -2.191 |
| 271.275 | 19.000 | 2-keto palmitic acid | 0.010 | 0.039 | -2.224 |
| 975.387 | 0.759 | Unidentified | 0.012 | 0.041 | -2.294 |
| 120.079 | 14.016 | L-Threonine | 0.000 | 0.009 | -2.360 |
| 166.086 | 14.016 | L-Phenylalanine | 0.000 | 0.009 | -2.421 |
| 499.338 | 0.750 | PG (8:0/8:0) | 0.005 | 0.027 | -2.433 |
| 192.101 | 14.021 | Unidentified | 0.000 | 0.008 | -2.505 |
| 408.105 | 4.195 | S-Succinylglutathione | 0.010 | 0.038 | -2.687 |
| 260.091 | 14.014 | Unidentified | 0.001 | 0.017 | -2.987 |
| 737.369 | 0.755 | Unidentified | 0.001 | 0.017 | -3.085 |
| 528.120 | 14.018 | Unidentified | 0.000 | 0.007 | -3.903 |
| 185.165 | 2.408 | 10-hendecenoic acid | 0.025 | 0.066 | -3.925 |

**Table S4.** Significantly Altered Metabolites in SAECs Exposed to 10 µg/mL PM from the Control Room.

| m/z | rt | Metabolite Name | p.value | FDR | log2(FC) |
| --- | --- | --- | --- | --- | --- |
| 786.602 | 21.302 | PC (18:1(9Z)/18:1(9Z)) [S] | 0.003 | 0.032 | 3.769 |
| 387.177 | 19.739 | 3,5-Di-O-methyl-8-prenylafzelechin-4beta-ol | 0.001 | 0.018 | 3.574 |
| 404.203 | 19.741 | 16-phenoxy tetranor PGF2α methyl amide | 0.001 | 0.018 | 3.376 |
| 432.238 | 19.741 | 17-phenoxy trinor PGF2α ethyl amide | 0.001 | 0.020 | 3.336 |
| 284.209 | 13.296 | Unidentified | 0.000 | 0.012 | 3.244 |
| 267.181 | 13.297 | Unidentified | 0.000 | 0.010 | 3.090 |
| 289.161 | 13.294 | Arginyl-Asparagine | 0.000 | 0.009 | 2.884 |
| 103.075 | 13.294 | Isovaleric acid | 0.000 | 0.014 | 2.824 |
| 766.535 | 21.462 | PC (15:0/20:5(5Z,8Z,11Z,14Z,17Z)) | 0.001 | 0.020 | 2.758 |
| 105.071 | 19.736 | L-2,3-DIAMINOPROPIONIC ACID | 0.000 | 0.012 | 2.221 |
| 590.426 | 21.548 | CerP(d18:1/14:0) | 0.023 | 0.103 | 1.973 |
| 652.412 | 17.163 | PS (14:0/12:0) | 0.001 | 0.018 | 1.943 |
| 240.182 | 11.679 | 2-Octyl-4-propylthiazole | 0.002 | 0.022 | 1.860 |
| 223.153 | 11.679 | Unidentified | 0.001 | 0.019 | 1.806 |
| 163.110 | 14.364 | 2-Hydroxyadipic acid | 0.003 | 0.029 | 1.804 |
| 268.209 | 11.683 | (2R,5S)-2,5-di((E)-pent-2-en-4-yn-1-yl) decahydroquinoline | 0.001 | 0.019 | 1.727 |
| 464.356 | 22.202 | D-Glucosyldihydrosphingosine | 0.001 | 0.020 | 1.571 |
| 245.136 | 11.679 | Polyethylene, oxidized | 0.001 | 0.019 | 1.551 |
| 546.398 | 21.549 | LysoPC(20:3(5Z,8Z,11Z)) | 0.008 | 0.058 | 1.485 |
| 608.386 | 16.452 | Unidentified | 0.001 | 0.019 | 1.429 |
| 147.103 | 11.681 | 2-hydroxy enanthoic acid | 0.003 | 0.031 | 1.381 |
| 217.070 | 1.618 | Bisnorbiotin | 0.002 | 0.028 | 1.365 |
| 493.198 | 2.561 | Unidentified | 0.009 | 0.061 | 1.359 |
| 285.090 | 8.626 | Cysteinyl-Tyrosine | 0.016 | 0.081 | 1.348 |
| 584.185 | 1.227 | Unidentified | 0.016 | 0.081 | 1.284 |
| 217.070 | 0.978 | 1,3,7-trimethyl-3,7-dihydro-1H-purine-2,6-dione | 0.000 | 0.014 | 1.166 |
| 258.094 | 2.561 | Unidentified | 0.011 | 0.067 | 1.116 |
| 268.136 | 11.140 | Unidentified | 0.002 | 0.027 | 1.069 |
| 187.094 | 6.665 | Unidentified | 0.012 | 0.068 | 1.023 |
| 87.042 | 12.443 | 4-Deoxytetronic acid | 0.015 | 0.078 | 1.021 |
| 492.388 | 22.203 | Glutathionylspermine | 0.014 | 0.077 | 1.001 |
| 186.074 | 2.569 | Unidentified | 0.004 | 0.039 | 0.959 |
| 236.112 | 2.569 | N-(9-oxodecyl) acetamide | 0.004 | 0.037 | 0.958 |
| 230.247 | 19.281 | Phosphoribosylamine | 0.037 | 0.135 | 0.928 |
| 482.422 | 22.119 | Ceramide (d18:1/12:0) | 0.002 | 0.024 | 0.905 |
| 564.362 | 15.690 | PC (18:1(9E)/2:0) | 0.000 | 0.014 | 0.901 |
| 904.403 | 3.290 | PS (22:0/22:0) | 0.040 | 0.139 | 0.891 |
| 574.434 | 21.550 | PS (22:4(7Z,10Z,13Z,16Z)/0:0) | 0.010 | 0.064 | 0.890 |
| 476.309 | 13.949 | N-docosahexaenoyl phenylalanine | 0.015 | 0.079 | 0.886 |
| 352.095 | 5.055 | Unidentified | 0.001 | 0.020 | 0.869 |
| 651.220 | 6.531 | Unidentified | 0.005 | 0.045 | 0.860 |
| 589.149 | 1.101 | 25-hydroxyvitamin D2 25-(β-glucuronide) / 25 hydroxyergocalciferol 25-(β-glucuronide) | 0.009 | 0.062 | 0.853 |
| 319.072 | 1.814 | Melanin | 0.012 | 0.069 | 0.849 |
| 455.166 | 0.981 | Phe-Phe4Cl-OH | 0.006 | 0.049 | 0.837 |
| 453.339 | 14.548 | (17E)-1α,25-dihydroxy-26,27-dimethyl-17,20,22,22,23,23-hexadehydro-24a-homovitamin D3 / (17E)-1α,25-dihydroxy-26,27-dimethyl-17,20,22,22,23,23-hexadehydro-24a-homocholecalciferol | 0.039 | 0.138 | 0.811 |
| 562.203 | 1.318 | Unidentified | 0.006 | 0.052 | 0.799 |
| 637.234 | 6.533 | Unidentified | 0.024 | 0.103 | 0.786 |
| 113.034 | 2.589 | Uracil | 0.000 | 0.014 | 0.774 |
| 233.062 | 2.474 | Unidentified | 0.000 | 0.012 | 0.766 |
| 679.234 | 5.035 | Unidentified | 0.000 | 0.002 | 0.760 |
| 247.045 | 2.470 | Glutamyl-Threonine | 0.013 | 0.073 | 0.759 |
| 324.106 | 1.310 | Unidentified | 0.002 | 0.023 | 0.755 |
| 418.252 | 1.990 | 16-phenoxy Prostaglandin F2a ethyl amide | 0.017 | 0.083 | 0.728 |
| 693.216 | 5.019 | Unidentified | 0.001 | 0.018 | 0.722 |
| 918.398 | 3.275 | Unidentified | 0.044 | 0.146 | 0.722 |
| 255.159 | 19.569 | Unidentified | 0.015 | 0.079 | 0.707 |
| 625.247 | 3.269 | Unidentified | 0.003 | 0.033 | 0.701 |
| 708.576 | 22.743 | PS(O-16:0/O-16:0) [U] | 0.002 | 0.025 | 0.701 |
| 663.444 | 22.730 | Prostaglandin E2-biotin | 0.039 | 0.138 | 0.677 |
| 157.044 | 7.526 | Unidentified | 0.000 | 0.004 | 0.667 |
| 226.106 | 8.129 | L-4-Hydroxy-3-methoxy-a-methylphenylalanine | 0.044 | 0.146 | 0.665 |
| 111.525 | 5.783 | Unidentified | 0.000 | 0.018 | 0.659 |
| 530.404 | 21.559 | Unidentified | 0.001 | 0.018 | 0.659 |
| 412.095 | 1.755 | Unidentified | 0.022 | 0.099 | 0.659 |
| 167.998 | 1.802 | 2,3-Pyridinedicarboxylic acid | 0.007 | 0.054 | 0.659 |
| 153.041 | 2.293 | Xanthine | 0.003 | 0.033 | 0.658 |
| 555.127 | 1.776 | 3-O-(6-O-alpha-D-Xylosylphospho-alpha-D-mannopyranosyl)-alpha-D-mannopyranose | 0.024 | 0.103 | 0.656 |
| 438.372 | 22.072 | (±) N-(1-methyl-2-hydroxy-2-phenyl-ethyl) arachidonyl amine | 0.004 | 0.036 | 0.651 |
| 680.472 | 22.775 | PS(O-16:0/13:0) | 0.024 | 0.103 | 0.638 |
| 297.038 | 1.765 | Unidentified | 0.009 | 0.062 | 0.635 |
| 165.112 | 6.665 | Unidentified | 0.015 | 0.078 | 0.627 |
| 520.199 | 4.299 | Unidentified | 0.001 | 0.018 | 0.624 |
| 326.119 | 7.458 | Citalopram (propionic acid derivative) | 0.001 | 0.018 | 0.623 |
| 168.065 | 1.436 | Pyridoxal (Vitamin B6) | 0.028 | 0.110 | 0.621 |
| 301.061 | 1.799 | Unidentified | 0.009 | 0.062 | 0.606 |
| 267.056 | 2.585 | Unidentified | 0.010 | 0.063 | 0.604 |
| 408.230 | 21.881 | Unidentified | 0.043 | 0.144 | 0.601 |
| 197.112 | 7.449 | Unidentified | 0.011 | 0.067 | 0.595 |
| 447.134 | 6.534 | Unidentified | 0.014 | 0.077 | 0.591 |
| 436.345 | 21.150 | Unidentified | 0.002 | 0.026 | 0.589 |
| 573.208 | 15.615 | Unidentified | 0.039 | 0.138 | 0.585 |
| 394.350 | 22.080 | N-palmitoyl histidine | 0.015 | 0.078 | 0.584 |
| 549.343 | 1.992 | 5beta-scymnol sulfate | 0.010 | 0.064 | 0.584 |
| 306.115 | 1.430 | Unidentified | 0.034 | 0.126 | 0.579 |
| 482.401 | 22.062 | PC(O-15:0/O-1:0) [U] | 0.002 | 0.027 | 0.574 |
| 724.504 | 22.768 | PC (14:1(9Z)/18:4(6Z,9Z,12Z,15Z)) | 0.040 | 0.139 | 0.573 |
| 198.070 | 7.524 | Unidentified | 0.001 | 0.019 | 0.570 |
| 110.974 | 1.231 | Unidentified | 0.023 | 0.102 | 0.567 |
| 293.172 | 1.067 | (E)-2-decylpent-2-enedioic acid | 0.001 | 0.019 | 0.567 |
| 110.036 | 2.300 | Hypotaurine | 0.005 | 0.045 | 0.560 |
| 238.071 | 2.638 | Unidentified | 0.043 | 0.144 | 0.555 |
| 301.145 | 2.018 | Unidentified | 0.001 | 0.020 | 0.550 |
| 137.047 | 1.820 | Hypoxanthine | 0.013 | 0.071 | 0.538 |
| 111.011 | 4.001 | Pyruvic acid | 0.012 | 0.069 | 0.538 |
| 210.044 | 7.521 | Unidentified | 0.005 | 0.046 | 0.538 |
| 369.110 | 4.301 | Unidentified | 0.001 | 0.018 | 0.536 |
| 496.474 | 21.797 | PS (16:0/0:0) | 0.001 | 0.020 | 0.536 |
| 426.079 | 1.749 | Hyaluronic acid | 0.021 | 0.097 | 0.535 |
| 309.226 | 17.938 | Unidentified | 0.027 | 0.108 | 0.530 |
| 209.128 | 1.081 | Unidentified | 0.001 | 0.020 | 0.528 |
| 658.502 | 22.003 | PE (12:0/18:3(6Z,9Z,12Z)) | 0.035 | 0.131 | 0.528 |
| 242.062 | 7.529 | N-(2,3-Dihydroxybenzoyl)-L-serine | 0.010 | 0.064 | 0.519 |
| 540.216 | 1.242 | Unidentified | 0.017 | 0.083 | 0.518 |
| 414.359 | 21.464 | Heptadecanoyl carnitine | 0.018 | 0.086 | 0.516 |
| 278.065 | 1.744 | 2-Phthalimidoglutaric acid | 0.002 | 0.024 | 0.515 |
| 534.192 | 4.296 | Unidentified | 0.015 | 0.078 | 0.505 |
| 174.088 | 1.074 | 2-Oxoarginine | 0.006 | 0.052 | 0.500 |
| 159.025 | 1.820 | fumarylacetic acid | 0.003 | 0.029 | 0.497 |
| 348.987 | 1.790 | Unidentified | 0.040 | 0.140 | 0.496 |
| 258.066 | 5.776 | Unidentified | 0.029 | 0.111 | 0.495 |
| 746.561 | 21.967 | PC (15:0/18:1(9Z))[U] | 0.000 | 0.014 | 0.489 |
| 323.161 | 6.721 | Nordurlettone | 0.038 | 0.137 | 0.488 |
| 514.410 | 21.803 | Sulfolithocholylglycine | 0.002 | 0.022 | 0.488 |
| 541.133 | 1.751 | Unidentified | 0.049 | 0.158 | 0.473 |
| 401.103 | 2.205 | S-Acetylphosphopantetheine | 0.001 | 0.018 | 0.469 |
| 305.148 | 6.735 | Unidentified | 0.050 | 0.158 | 0.465 |
| 195.136 | 18.684 | Unidentified | 0.031 | 0.119 | 0.465 |
| 526.428 | 22.055 | Unidentified | 0.004 | 0.038 | 0.462 |
| 226.179 | 18.958 | (8S, Z)-6-((S)-3-hydroxy-2-methylpropylidene)-8-methyloctahydroindolizin-8-ol | 0.030 | 0.117 | 0.447 |
| 324.131 | 1.430 | Unidentified | 0.040 | 0.139 | 0.432 |
| 315.151 | 1.067 | Unidentified | 0.005 | 0.046 | 0.426 |
| 162.078 | 6.751 | L-2-Aminoadipic acid | 0.019 | 0.092 | 0.409 |
| 685.284 | 4.297 | gamma-L-Glutamyl-butirosin B | 0.043 | 0.144 | 0.405 |
| 375.995 | 1.654 | Unidentified | 0.000 | 0.014 | 0.396 |
| 672.531 | 22.365 | GlcCer(d18:1/14:0) | 0.018 | 0.086 | 0.393 |
| 241.100 | 7.442 | Unidentified | 0.008 | 0.060 | 0.393 |
| 400.342 | 21.112 | Palmitoyl-L-carnitine | 0.018 | 0.086 | 0.393 |
| 604.214 | 2.645 | Unidentified | 0.036 | 0.133 | 0.392 |
| 340.260 | 11.485 | Unidentified | 0.039 | 0.139 | 0.389 |
| 582.174 | 2.205 | Unidentified | 0.002 | 0.027 | 0.388 |
| 384.343 | 22.737 | N-stearoyl valine | 0.008 | 0.058 | 0.386 |
| 498.417 | 21.722 | Cer(d18:0/13:0) | 0.039 | 0.138 | 0.375 |
| 321.144 | 6.739 | Unidentified | 0.009 | 0.061 | 0.369 |
| 258.110 | 6.751 | Glycerophosphocholine | 0.027 | 0.108 | 0.354 |
| 516.303 | 13.725 | Taurallocholic acid | 0.005 | 0.043 | 0.351 |
| 176.119 | 4.799 | 7-Methylthioheptanaldoxime | 0.001 | 0.020 | 0.350 |
| 358.298 | 20.278 | N-palmitoyl threonine | 0.009 | 0.062 | 0.350 |
| 275.162 | 1.070 | Unidentified | 0.027 | 0.108 | 0.350 |
| 628.504 | 22.394 | Cer(t18:0/20:0(2OH)) | 0.022 | 0.099 | 0.349 |
| 249.055 | 3.655 | Unidentified | 0.026 | 0.105 | 0.346 |
| 218.136 | 3.155 | Propionyl-L-carnitine | 0.015 | 0.078 | 0.336 |
| 100.037 | 15.369 | Succinimide | 0.007 | 0.052 | 0.333 |
| 442.388 | 21.800 | Leukotriene E3 | 0.001 | 0.018 | 0.323 |
| 367.266 | 18.648 | Unidentified | 0.038 | 0.138 | 0.321 |
| 303.135 | 6.743 | Unidentified | 0.025 | 0.104 | 0.304 |
| 460.151 | 1.038 | Phe-Trp-OH | 0.023 | 0.103 | 0.299 |
| 72.082 | 1.092 | Unidentified | 0.006 | 0.051 | 0.295 |
| 338.343 | 21.906 | Unidentified | 0.025 | 0.104 | 0.288 |
| 548.170 | 2.168 | Unidentified | 0.011 | 0.067 | 0.286 |
| 118.087 | 1.095 | L-Valine | 0.019 | 0.089 | 0.281 |
| 402.246 | 19.772 | Unidentified | 0.040 | 0.139 | 0.274 |
| 250.109 | 5.008 | Cysteinyl-Lysine | 0.001 | 0.020 | 0.268 |
| 156.079 | 6.748 | Histidine | 0.041 | 0.140 | 0.267 |
| 225.088 | 0.801 | 3-Hydroxy-DL-kynurenine | 0.011 | 0.067 | 0.259 |
| 276.120 | 6.746 | Norophthalmic acid | 0.050 | 0.158 | 0.259 |
| 114.091 | 7.095 | Unidentified | 0.041 | 0.140 | 0.251 |
| 412.377 | 22.049 | PC(O-8:0/2:0) | 0.029 | 0.111 | 0.250 |
| 86.096 | 4.679 | 2-Methylpyrrolidine | 0.001 | 0.019 | 0.245 |
| 642.519 | 22.062 | GlcCer(d14:2(4E,6E)/16:0) | 0.043 | 0.144 | 0.240 |
| 366.094 | 5.041 | 2-S-Glutathionyl acetate | 0.003 | 0.034 | 0.238 |
| 228.101 | 5.025 | Unidentified | 0.003 | 0.031 | 0.236 |
| 428.373 | 21.645 | C8-Dihydroceramide | 0.049 | 0.156 | 0.229 |
| 337.175 | 0.774 | Unidentified | 0.016 | 0.083 | 0.217 |
| 357.160 | 1.664 | xi-3-Hydroxy-5-phenylpentanoic acid O-beta-D-Glucopyranoside | 0.036 | 0.133 | 0.216 |
| 117.056 | 5.014 | α-ketoisovaleric acid | 0.026 | 0.105 | 0.211 |
| 232.118 | 1.068 | Suberylglycine | 0.018 | 0.088 | 0.207 |
| 115.053 | 5.012 | Unidentified | 0.010 | 0.064 | 0.189 |
| 144.079 | 5.017 | Unidentified | 0.002 | 0.027 | 0.187 |
| 174.015 | 1.671 | Adenine | 0.048 | 0.155 | 0.187 |
| 168.079 | 6.753 | N-Trimethyl-2-aminoethylphosphonate | 0.042 | 0.140 | 0.170 |
| 261.086 | 2.163 | Unidentified | 0.005 | 0.042 | 0.163 |
| 442.164 | 2.186 | N-(4-hydroxyphenethyl)-2-(2,3,4,9-tetramethyl-7-oxo-7H-furo[2,3-f] chromen-8-yl) acetamide | 0.045 | 0.147 | 0.163 |
| 105.043 | 6.540 | 3-Cyanopyridine | 0.025 | 0.104 | 0.159 |
| 156.101 | 1.395 | Unidentified | 0.048 | 0.155 | 0.151 |
| 260.113 | 1.069 | Unidentified | 0.041 | 0.140 | 0.147 |
| 91.055 | 5.016 | Unidentified | 0.005 | 0.043 | 0.141 |
| 309.128 | 0.790 | Unidentified | 0.002 | 0.028 | 0.136 |
| 132.082 | 5.010 | Unidentified | 0.021 | 0.099 | 0.135 |
| 221.092 | 6.552 | 5-Hydroxy-L-tryptophan | 0.024 | 0.103 | 0.133 |
| 520.215 | 0.785 | Unidentified | 0.025 | 0.104 | 0.127 |
| 310.126 | 5.021 | Unidentified | 0.041 | 0.140 | 0.123 |
| 264.121 | 5.021 | 7-Mercaptoheptanoylthreonine | 0.023 | 0.102 | 0.123 |
| 212.127 | 3.269 | Unidentified | 0.027 | 0.109 | 0.119 |
| 328.139 | 5.014 | PC(O-2:0/2:0) | 0.010 | 0.064 | 0.116 |
| 162.091 | 5.016 | 1-(2,3-Dihydro-1H-pyrrolizin-5-yl)-2-propen-1-one | 0.041 | 0.140 | 0.115 |
| 114.046 | 6.538 | Unidentified | 0.019 | 0.089 | 0.110 |
| 132.102 | 2.014 | L-Isoleucine | 0.017 | 0.083 | 0.100 |
| 102.056 | 1.715 | Unidentified | 0.024 | 0.103 | 0.096 |
| 246.112 | 5.034 | Asparaginyl-Hydroxyproline | 0.027 | 0.109 | 0.095 |
| 174.092 | 5.029 | Unidentified | 0.045 | 0.149 | 0.069 |
| 105.071 | 5.016 | Unidentified | 0.032 | 0.121 | 0.055 |
| 97.028 | 5.033 | Methaneselenol | 0.045 | 0.147 | 0.049 |
| 103.111 | 4.294 | 3-Methylpentan-1-ol | 0.049 | 0.157 | -0.037 |
| 107.052 | 4.299 | Unidentified | 0.025 | 0.103 | -0.054 |
| 70.074 | 1.961 | 1-Pyrroline | 0.046 | 0.150 | -0.063 |
| 86.504 | 1.989 | L-Homocysteine sulfonic acid | 0.037 | 0.134 | -0.094 |
| 535.198 | 18.189 | 5''-Phosphoribostamycin | 0.045 | 0.149 | -0.114 |
| 334.140 | 10.201 | Unidentified | 0.004 | 0.036 | -0.125 |
| 398.987 | 0.896 | Unidentified | 0.014 | 0.076 | -0.133 |
| 106.066 | 1.848 | 3-Methylthiopropanamine | 0.013 | 0.073 | -0.138 |
| 152.038 | 0.774 | Unidentified | 0.006 | 0.047 | -0.144 |
| 277.064 | 0.770 | Unidentified | 0.003 | 0.032 | -0.159 |
| 182.083 | 2.208 | L-Tyrosine | 0.012 | 0.069 | -0.160 |
| 95.050 | 2.221 | Phenol | 0.012 | 0.069 | -0.172 |
| 165.055 | 2.221 | Unidentified | 0.023 | 0.103 | -0.173 |
| 473.177 | 2.162 | C1'-C9-Glycosylated UWM6 | 0.010 | 0.063 | -0.174 |
| 114.091 | 1.402 | Unidentified | 0.001 | 0.018 | -0.174 |
| 130.048 | 9.874 | Pyroglutamic acid | 0.038 | 0.137 | -0.175 |
| 123.046 | 2.221 | THIODIGLYCOL | 0.011 | 0.067 | -0.175 |
| 363.157 | 2.202 | Unidentified | 0.014 | 0.077 | -0.177 |
| 603.204 | 0.962 | Unidentified | 0.048 | 0.156 | -0.179 |
| 136.078 | 2.221 | 2-Phenylacetamide | 0.016 | 0.082 | -0.181 |
| 117.058 | 2.220 | Ketoisovaleric acid | 0.027 | 0.109 | -0.182 |
| 143.056 | 1.404 | Unidentified | 0.024 | 0.103 | -0.188 |
| 147.046 | 2.223 | Coumaric acid | 0.025 | 0.104 | -0.190 |
| 261.023 | 18.642 | D-Galactose 6-sulfate | 0.028 | 0.110 | -0.191 |
| 331.166 | 4.297 | 8-Hydroxyclomipramine | 0.016 | 0.083 | -0.192 |
| 94.042 | 2.222 | Unidentified | 0.003 | 0.034 | -0.193 |
| 91.056 | 2.221 | Unidentified | 0.008 | 0.060 | -0.198 |
| 119.048 | 2.221 | Allophanic acid methyl ester | 0.022 | 0.101 | -0.198 |
| 458.692 | 17.498 | 7-methylguanosine 5'-diphosphate | 0.005 | 0.045 | -0.200 |
| 203.052 | 1.032 | Unidentified | 0.011 | 0.067 | -0.207 |
| 107.052 | 2.221 | Unidentified | 0.047 | 0.154 | -0.211 |
| 77.039 | 14.015 | Unidentified | 0.033 | 0.122 | -0.220 |
| 121.067 | 2.224 | 4-deoxy-threonic acid | 0.009 | 0.061 | -0.225 |
| 355.066 | 18.653 | Phenolsulfonphthalein | 0.014 | 0.076 | -0.226 |
| 280.139 | 1.403 | Unidentified | 0.022 | 0.101 | -0.231 |
| 758.136 | 16.238 | Peonidin 3-sambubioside 5-glucoside | 0.001 | 0.020 | -0.242 |
| 180.101 | 1.403 | Unidentified | 0.008 | 0.060 | -0.243 |
| 319.035 | 5.778 | Unidentified | 0.028 | 0.110 | -0.253 |
| 145.048 | 4.997 | 3-hexenedioic acid | 0.006 | 0.046 | -0.254 |
| 687.212 | 19.401 | Unidentified | 0.028 | 0.110 | -0.264 |
| 127.038 | 1.410 | Unidentified | 0.009 | 0.061 | -0.266 |
| 353.113 | 16.244 | Penicilloic G acid | 0.030 | 0.115 | -0.268 |
| 262.130 | 1.401 | Unidentified | 0.017 | 0.083 | -0.268 |
| 161.068 | 1.405 | Unidentified | 0.007 | 0.053 | -0.270 |
| 571.141 | 4.297 | Unidentified | 0.003 | 0.031 | -0.272 |
| 211.167 | 19.450 | 3E,5E-tridecadienoic acid | 0.014 | 0.076 | -0.273 |
| 105.000 | 1.281 | β-Hydroxypyruvic acid | 0.024 | 0.103 | -0.274 |
| 90.054 | 2.340 | Alanine | 0.003 | 0.032 | -0.274 |
| 130.202 | 1.696 | Octylamine | 0.023 | 0.102 | -0.275 |
| 334.975 | 1.198 | Unidentified | 0.029 | 0.113 | -0.277 |
| 171.982 | 4.190 | Calcium L-aspartate | 0.028 | 0.110 | -0.278 |
| 85.029 | 1.293 | Unidentified | 0.002 | 0.027 | -0.280 |
| 216.124 | 1.399 | Unidentified | 0.017 | 0.083 | -0.284 |
| 134.998 | 1.192 | Malic acid | 0.000 | 0.008 | -0.287 |
| 101.004 | 1.135 | Sodium fluoroacetate | 0.011 | 0.067 | -0.289 |
| 198.113 | 1.405 | Metanephrine | 0.007 | 0.057 | -0.290 |
| 79.022 | 1.135 | Unidentified | 0.009 | 0.061 | -0.292 |
| 139.004 | 1.562 | Unidentified | 0.048 | 0.154 | -0.294 |
| 202.108 | 1.394 | (E)-1-(4-methylquinazolin-2(1H)-ylidene)guanidine | 0.000 | 0.014 | -0.297 |
| 244.121 | 1.406 | Glutaminyl-Proline | 0.033 | 0.123 | -0.299 |
| 152.055 | 1.279 | Guanine | 0.000 | 0.008 | -0.300 |
| 290.126 | 3.565 | Unidentified | 0.010 | 0.064 | -0.317 |
| 228.085 | 4.069 | L-Arogenate | 0.001 | 0.019 | -0.327 |
| 384.022 | 18.618 | Unidentified | 0.041 | 0.140 | -0.331 |
| 234.135 | 1.398 | Unidentified | 0.012 | 0.070 | -0.331 |
| 549.157 | 4.298 | Daidzein 7-O-glucoside-4'-O-apioside | 0.018 | 0.086 | -0.333 |
| 175.088 | 3.096 | Unidentified | 0.000 | 0.002 | -0.334 |
| 120.045 | 9.883 | Aminomalonic acid | 0.000 | 0.012 | -0.338 |
| 204.123 | 12.327 | Acetylcarnitine | 0.025 | 0.103 | -0.355 |
| 454.113 | 9.043 | Unidentified | 0.006 | 0.046 | -0.355 |
| 106.048 | 1.282 | L-Serine | 0.010 | 0.064 | -0.357 |
| 106.041 | 9.874 | 2-cyano-Pyrimidine | 0.032 | 0.120 | -0.359 |
| 369.075 | 19.950 | 2-Deoxy-4-O-[(2E)-3-(4-hydroxyphenyl)-2-propenoyl]-3-C-(methoxycarbonyl)pentaric acid | 0.002 | 0.027 | -0.368 |
| 492.139 | 5.771 | Unidentified | 0.010 | 0.064 | -0.370 |
| 133.034 | 1.283 | Unidentified | 0.000 | 0.002 | -0.376 |
| 284.058 | 16.265 | N2-Acetyl-L-aminoadipyl-δ-phosphate | 0.041 | 0.140 | -0.377 |
| 150.058 | 1.281 | L-Methionine | 0.000 | 0.007 | -0.379 |
| 714.235 | 4.297 | PS (13:0/18:4(6Z,9Z,12Z,15Z)) | 0.008 | 0.060 | -0.396 |
| 182.210 | 2.216 | Unidentified | 0.004 | 0.036 | -0.399 |
| 102.056 | 1.283 | Unidentified | 0.000 | 0.002 | -0.400 |
| 280.231 | 1.408 | Linoleamide | 0.021 | 0.097 | -0.405 |
| 104.054 | 1.283 | Biuret | 0.000 | 0.009 | -0.407 |
| 313.105 | 9.874 | Thr-Ala-OH | 0.001 | 0.019 | -0.412 |
| 421.425 | 20.069 | 27:2(5Z,9Z) (25Me) | 0.010 | 0.064 | -0.414 |
| 295.093 | 9.876 | 4-(o-Carboxybenzamido)glutaramic acid | 0.009 | 0.063 | -0.416 |
| 442.148 | 9.874 | Folic acid | 0.024 | 0.103 | -0.416 |
| 223.076 | 4.779 | L-Cystathionine | 0.010 | 0.064 | -0.421 |
| 142.123 | 3.529 | Unidentified | 0.001 | 0.018 | -0.421 |
| 148.053 | 9.879 | L-Glutamate | 0.005 | 0.042 | -0.422 |
| 102.057 | 9.879 | Unidentified | 0.007 | 0.052 | -0.438 |
| 238.071 | 1.920 | Unidentified | 0.010 | 0.064 | -0.443 |
| 74.060 | 1.290 | Unidentified | 0.003 | 0.029 | -0.445 |
| 167.094 | 1.133 | N-Formyl-4-amino-5-aminomethyl-2-methylpyrimidine | 0.004 | 0.036 | -0.449 |
| 354.075 | 4.780 | Unidentified | 0.012 | 0.069 | -0.449 |
| 581.256 | 1.371 | Unidentified | 0.050 | 0.158 | -0.466 |
| 196.099 | 1.414 | L-Tyrosine methyl ester | 0.008 | 0.060 | -0.469 |
| 84.043 | 4.709 | 1-Methylpyrrolinium | 0.017 | 0.084 | -0.490 |
| 123.107 | 2.228 | Unidentified | 0.000 | 0.009 | -0.490 |
| 643.225 | 19.440 | Unidentified | 0.000 | 0.008 | -0.515 |
| 241.078 | 1.632 | Unidentified | 0.001 | 0.019 | -0.520 |
| 612.187 | 1.396 | Unidentified | 0.022 | 0.101 | -0.524 |
| 483.040 | 1.184 | Thymidine 5'-triphosphate | 0.008 | 0.060 | -0.524 |
| 281.210 | 1.404 | 12S-hydroxy-5Z,8E,10E-heptadecatrienoic acid | 0.039 | 0.138 | -0.538 |
| 280.312 | 1.409 | Unidentified | 0.050 | 0.158 | -0.570 |
| 158.155 | 19.484 | Unidentified | 0.010 | 0.063 | -0.597 |
| 303.068 | 5.293 | Unidentified | 0.000 | 0.014 | -0.613 |
| 386.212 | 19.422 | Unidentified | 0.037 | 0.135 | -0.630 |
| 670.398 | 18.996 | PE (13:0/18:4(6Z,9Z,12Z,15Z)) | 0.033 | 0.124 | -0.651 |
| 103.054 | 14.021 | Unidentified | 0.013 | 0.071 | -0.652 |
| 262.321 | 1.402 | Unidentified | 0.029 | 0.113 | -0.658 |
| 267.096 | 9.892 | Unidentified | 0.006 | 0.052 | -0.675 |
| 388.158 | 1.269 | Unidentified | 0.002 | 0.023 | -0.679 |
| 194.023 | 1.263 | Unidentified | 0.010 | 0.064 | -0.687 |
| 112.051 | 1.268 | Cytosine | 0.001 | 0.018 | -0.693 |
| 120.079 | 14.016 | L-Threonine | 0.025 | 0.104 | -0.710 |
| 256.082 | 1.914 | Nicotinate D-ribonucleoside | 0.002 | 0.022 | -0.722 |
| 240.230 | 19.002 | Unidentified | 0.023 | 0.103 | -0.740 |
| 125.538 | 9.865 | Unidentified | 0.003 | 0.034 | -0.745 |
| 221.092 | 4.891 | 5-Hydroxytryptophan | 0.039 | 0.138 | -0.758 |
| 438.304 | 19.409 | PE(P-16:0/0:0) | 0.007 | 0.052 | -0.762 |
| 238.108 | 14.016 | N-lactoyl-phenylalanine | 0.008 | 0.060 | -0.762 |
| 166.086 | 14.016 | L-Phenylalanine | 0.015 | 0.079 | -0.768 |
| 192.101 | 14.021 | Unidentified | 0.014 | 0.075 | -0.794 |
| 440.325 | 19.239 | PC(O-12:0/O-1:0) | 0.000 | 0.014 | -0.803 |
| 330.058 | 1.959 | cAMP | 0.001 | 0.018 | -0.822 |
| 314.084 | 1.945 | Unidentified | 0.000 | 0.007 | -0.832 |
| 337.021 | 14.018 | Unidentified | 0.002 | 0.022 | -0.845 |
| 172.042 | 1.275 | Unidentified | 0.002 | 0.029 | -0.895 |
| 145.106 | 0.953 | 4-Guanidinobutanamide | 0.005 | 0.045 | -0.940 |
| 674.429 | 19.078 | Unidentified | 0.019 | 0.088 | -0.948 |
| 274.090 | 1.944 | Pentose + Proline | 0.001 | 0.018 | -0.968 |
| 260.091 | 14.014 | Unidentified | 0.035 | 0.129 | -0.974 |
| 398.242 | 20.069 | PC (9:0/0:0) | 0.011 | 0.068 | -0.981 |
| 630.402 | 19.036 | Unidentified | 0.017 | 0.083 | -0.993 |
| 144.100 | 8.861 | Unidentified | 0.000 | 0.002 | -1.022 |
| 208.099 | 8.873 | 4-(2-Aminophenyl)-2,4-dioxobutanoic acid | 0.005 | 0.043 | -1.035 |
| 891.311 | 1.402 | dipalmitoyl phosphatidylinositol 3-phosphate | 0.007 | 0.052 | -1.070 |
| 784.562 | 22.733 | Arachidonoyl thio-PC | 0.001 | 0.020 | -1.081 |
| 542.357 | 18.984 | PC (20:5(5Z,8Z,11Z,14Z,17Z)/0:0) | 0.004 | 0.038 | -1.082 |
| 423.194 | 1.972 | Unidentified | 0.001 | 0.019 | -1.083 |
| 552.182 | 1.974 | N-Acetyl-N6, N6,O-tridemethylpuromycin-5'-phosphate | 0.001 | 0.019 | -1.085 |
| 270.314 | 19.617 | Unidentified | 0.032 | 0.121 | -1.135 |
| 432.181 | 20.065 | Lys-TyrMe-OH | 0.044 | 0.146 | -1.156 |
| 254.099 | 8.874 | Dihydroferuloylglycine | 0.001 | 0.020 | -1.157 |
| 190.109 | 8.857 | Unidentified | 0.001 | 0.018 | -1.193 |
| 348.123 | 20.069 | Unidentified | 0.032 | 0.121 | -1.208 |
| 350.119 | 20.060 | Unidentified | 0.012 | 0.069 | -1.337 |
| 528.120 | 14.018 | Unidentified | 0.002 | 0.027 | -1.382 |
| 605.175 | 1.935 | SP1 | 0.001 | 0.020 | -1.424 |
| 289.120 | 8.997 | Unidentified | 0.040 | 0.139 | -1.634 |
| 113.108 | 3.814 | Hydroxymethylphosphonate | 0.002 | 0.022 | -1.684 |
| 271.275 | 19.000 | 2-keto palmitic acid | 0.020 | 0.092 | -1.822 |
| 773.492 | 20.068 | PA (20:2(11Z,14Z)/22:6(4Z,7Z,10Z,13Z,16Z,19Z)) | 0.022 | 0.101 | -1.884 |
| 113.107 | 5.786 | 2-Imino-4-methylpiperidine | 0.002 | 0.022 | -1.908 |
| 504.176 | 1.260 | TyrMe-Trp-OH | 0.001 | 0.018 | -2.241 |

**Table S5.** Significantly Altered Metabolites in SAECs Exposed to 10 µg/mL PM Collected from the Printer Room During Printing with ABS Filaments.

| m/z | rt | Metabolite Name | p.value | FDR | log2(FC) |
| --- | --- | --- | --- | --- | --- |
| 387.177 | 19.739 | 3,5-Di-O-methyl-8-prenylafzelechin-4beta-ol | 0.000 | 0.014 | 4.907 |
| 786.602 | 21.302 | PC (18:1(9Z)/18:1(9Z))[S] | 0.000 | 0.007 | 4.665 |
| 404.203 | 19.741 | 16-phenoxy tetranor PGF2α methyl amide | 0.001 | 0.015 | 4.661 |
| 432.238 | 19.741 | 17-phenoxy trinor PGF2α ethyl amide | 0.001 | 0.016 | 4.325 |
| 284.209 | 13.296 | Unidentified | 0.000 | 0.013 | 3.783 |
| 87.042 | 12.443 | 4-Deoxytetronic acid | 0.005 | 0.038 | 3.638 |
| 412.320 | 19.839 | Unidentified | 0.021 | 0.086 | 3.636 |
| 267.181 | 13.297 | Unidentified | 0.000 | 0.013 | 3.545 |
| 289.161 | 13.294 | Arginyl-Asparagine | 0.000 | 0.008 | 3.476 |
| 105.071 | 19.736 | L-2,3-DIAMINOPROPIONIC ACID | 0.001 | 0.014 | 3.470 |
| 103.075 | 13.294 | Isovaleric acid | 0.000 | 0.013 | 3.132 |
| 766.535 | 21.462 | PC (15:0/20:5(5Z,8Z,11Z,14Z,17Z)) | 0.004 | 0.032 | 2.741 |
| 110.036 | 2.300 | Hypotaurine | 0.019 | 0.081 | 2.569 |
| 708.576 | 22.743 | PS(O-16:0/O-16:0) [U] | 0.026 | 0.096 | 2.445 |
| 652.412 | 17.163 | PS (14:0/12:0) | 0.000 | 0.013 | 2.433 |
| 240.182 | 11.679 | 2-Octyl-4-propylthiazole | 0.002 | 0.027 | 2.284 |
| 268.209 | 11.683 | (2R,5S)-2,5-di((E)-pent-2-en-4-yn-1-yl) decahydroquinoline | 0.000 | 0.014 | 2.206 |
| 223.153 | 11.679 | Unidentified | 0.002 | 0.027 | 2.204 |
| 268.102 | 3.877 | Adenosine | 0.002 | 0.023 | 2.037 |
| 245.136 | 11.679 | Polyethylene, oxidized | 0.002 | 0.027 | 1.995 |
| 493.198 | 2.561 | Unidentified | 0.000 | 0.014 | 1.992 |
| 163.110 | 14.364 | 2-Hydroxyadipic acid | 0.001 | 0.017 | 1.984 |
| 590.426 | 21.548 | CerP(d18:1/14:0) | 0.001 | 0.014 | 1.950 |
| 147.103 | 11.681 | 2-hydroxy enanthoic acid | 0.006 | 0.038 | 1.683 |
| 546.398 | 21.549 | LysoPC(20:3(5Z,8Z,11Z)) | 0.004 | 0.035 | 1.680 |
| 159.025 | 1.820 | fumarylacetic acid | 0.000 | 0.005 | 1.641 |
| 564.362 | 15.690 | PC (18:1(9E)/2:0) | 0.011 | 0.057 | 1.631 |
| 476.309 | 13.949 | N-docosahexaenoyl phenylalanine | 0.007 | 0.044 | 1.609 |
| 608.386 | 16.452 | Unidentified | 0.002 | 0.023 | 1.584 |
| 399.250 | 21.164 | 4,5-epoxy-17R-HDHA | 0.047 | 0.133 | 1.567 |
| 137.047 | 1.820 | Hypoxanthine | 0.000 | 0.007 | 1.542 |
| 444.310 | 21.161 | 17-phenyl trinor PGF2α diethyl amide | 0.032 | 0.107 | 1.518 |
| 384.115 | 6.653 | Succinoadenosine | 0.046 | 0.132 | 1.474 |
| 153.041 | 2.293 | Xanthine | 0.000 | 0.006 | 1.468 |
| 589.149 | 1.101 | 25-hydroxyvitamin D2 25-(β-glucuronide) / 25 hydroxyergocalciferol 25-(β-glucuronide) | 0.006 | 0.038 | 1.456 |
| 258.094 | 2.561 | Unidentified | 0.000 | 0.011 | 1.450 |
| 236.112 | 2.569 | N-(9-oxodecyl) acetamide | 0.000 | 0.013 | 1.429 |
| 663.444 | 22.730 | Prostaglandin E2-biotin | 0.004 | 0.032 | 1.394 |
| 186.074 | 2.569 | Unidentified | 0.001 | 0.015 | 1.388 |
| 680.472 | 22.775 | PS(O-16:0/13:0) | 0.004 | 0.035 | 1.264 |
| 464.356 | 22.202 | D-Glucosyldihydrosphingosine | 0.004 | 0.033 | 1.213 |
| 300.199 | 9.258 | Unidentified | 0.032 | 0.106 | 1.207 |
| 453.339 | 14.548 | (17E)-1α,25-dihydroxy-26,27-dimethyl-17,20,22,22,23,23-hexadehydro-24a-homovitamin D3 / (17E)-1α,25-dihydroxy-26,27-dimethyl-17,20,22,22,23,23-hexadehydro-24a-homocholecalciferol | 0.016 | 0.071 | 1.168 |
| 584.185 | 1.227 | Unidentified | 0.028 | 0.100 | 1.157 |
| 217.070 | 1.618 | Bisnorbiotin | 0.038 | 0.117 | 1.121 |
| 168.065 | 1.436 | Pyridoxal (Vitamin B6) | 0.020 | 0.082 | 1.107 |
| 708.511 | 22.725 | PS(O-16:0/15:0) | 0.024 | 0.091 | 1.103 |
| 230.247 | 19.281 | Phosphoribosylamine | 0.020 | 0.082 | 1.087 |
| 324.131 | 1.430 | Unidentified | 0.003 | 0.028 | 1.054 |
| 306.115 | 1.430 | Unidentified | 0.007 | 0.042 | 1.021 |
| 460.270 | 20.183 | 17-phenyl trinor Prostaglandin E2 serinol amide | 0.031 | 0.105 | 1.017 |
| 268.136 | 11.140 | Unidentified | 0.000 | 0.012 | 1.009 |
| 187.094 | 6.665 | Unidentified | 0.005 | 0.036 | 0.992 |
| 209.137 | 8.613 | 2,4-Diphenyl-1-butene | 0.001 | 0.015 | 0.983 |
| 637.234 | 6.533 | Unidentified | 0.004 | 0.033 | 0.975 |
| 111.011 | 4.001 | Pyruvic acid | 0.023 | 0.090 | 0.964 |
| 165.112 | 6.665 | Unidentified | 0.010 | 0.051 | 0.944 |
| 381.337 | 22.291 | 15-oxo-18Z-tetracosenoic acid | 0.006 | 0.039 | 0.931 |
| 482.422 | 22.119 | Ceramide (d18:1/12:0) | 0.002 | 0.025 | 0.907 |
| 113.034 | 2.589 | Uracil | 0.022 | 0.087 | 0.885 |
| 110.974 | 1.231 | Unidentified | 0.042 | 0.125 | 0.879 |
| 574.434 | 21.550 | PS (22:4(7Z,10Z,13Z,16Z)/0:0) | 0.030 | 0.103 | 0.875 |
| 736.536 | 22.760 | PS(O-18:0/15:0) | 0.029 | 0.102 | 0.848 |
| 651.220 | 6.531 | Unidentified | 0.001 | 0.022 | 0.845 |
| 167.998 | 1.802 | 2,3-Pyridinedicarboxylic acid | 0.004 | 0.033 | 0.841 |
| 298.117 | 5.348 | 1-Methylguanosine | 0.010 | 0.051 | 0.838 |
| 237.085 | 4.957 | N, N-Dihydroxy-L-tryptophan | 0.011 | 0.054 | 0.831 |
| 176.119 | 4.799 | 7-Methylthioheptanaldoxime | 0.005 | 0.037 | 0.816 |
| 418.252 | 1.990 | 16-phenoxy Prostaglandin F2a ethyl amide | 0.012 | 0.060 | 0.815 |
| 255.159 | 19.569 | Unidentified | 0.005 | 0.038 | 0.801 |
| 187.032 | 18.681 | Unidentified | 0.036 | 0.114 | 0.800 |
| 520.199 | 4.299 | Unidentified | 0.000 | 0.007 | 0.796 |
| 555.127 | 1.776 | 3-O-(6-O-alpha-D-Xylosylphospho-alpha-D-mannopyranosyl)-alpha-D-mannopyranose | 0.017 | 0.074 | 0.791 |
| 352.095 | 5.055 | Unidentified | 0.002 | 0.024 | 0.775 |
| 297.038 | 1.765 | Unidentified | 0.008 | 0.048 | 0.773 |
| 319.072 | 1.814 | Melanin | 0.018 | 0.077 | 0.762 |
| 344.238 | 2.705 | Unidentified | 0.023 | 0.091 | 0.749 |
| 278.065 | 1.744 | 2-Phthalimidoglutaric acid | 0.005 | 0.038 | 0.749 |
| 157.044 | 7.526 | Unidentified | 0.003 | 0.029 | 0.744 |
| 549.343 | 1.992 | 5beta-scymnol sulfate | 0.005 | 0.037 | 0.738 |
| 412.095 | 1.755 | Unidentified | 0.017 | 0.073 | 0.738 |
| 530.404 | 21.559 | Unidentified | 0.043 | 0.127 | 0.734 |
| 492.388 | 22.203 | Glutathionylspermine | 0.037 | 0.115 | 0.730 |
| 301.061 | 1.799 | Unidentified | 0.009 | 0.048 | 0.729 |
| 679.234 | 5.035 | Unidentified | 0.002 | 0.023 | 0.710 |
| 482.401 | 22.062 | PC(O-15:0/O-1:0) [U] | 0.002 | 0.027 | 0.707 |
| 368.425 | 21.310 | Unidentified | 0.049 | 0.136 | 0.707 |
| 162.039 | 18.676 | N-Formyl-L-aspartate | 0.003 | 0.028 | 0.706 |
| 693.216 | 5.019 | Unidentified | 0.014 | 0.066 | 0.691 |
| 401.103 | 2.205 | S-Acetylphosphopantetheine | 0.000 | 0.007 | 0.683 |
| 301.145 | 2.018 | Unidentified | 0.003 | 0.028 | 0.680 |
| 380.163 | 18.682 | Unidentified | 0.044 | 0.129 | 0.678 |
| 324.106 | 1.310 | Unidentified | 0.009 | 0.050 | 0.678 |
| 238.071 | 2.638 | Unidentified | 0.013 | 0.063 | 0.673 |
| 242.062 | 7.529 | N-(2,3-Dihydroxybenzoyl)-L-serine | 0.000 | 0.011 | 0.673 |
| 426.079 | 1.749 | Hyaluronic acid | 0.016 | 0.072 | 0.669 |
| 394.350 | 22.080 | N-palmitoyl histidine | 0.016 | 0.072 | 0.668 |
| 438.372 | 22.072 | (±) N-(1-methyl-2-hydroxy-2-phenyl-ethyl) arachidonyl amine | 0.003 | 0.031 | 0.668 |
| 541.133 | 1.751 | Unidentified | 0.031 | 0.105 | 0.664 |
| 447.134 | 6.534 | Unidentified | 0.013 | 0.063 | 0.663 |
| 236.128 | 7.254 | Unidentified | 0.031 | 0.104 | 0.652 |
| 414.359 | 21.464 | Heptadecanoyl carnitine | 0.009 | 0.050 | 0.650 |
| 426.393 | 22.298 | Vaccenyl carnitine | 0.027 | 0.098 | 0.638 |
| 658.502 | 22.003 | PE (12:0/18:3(6Z,9Z,12Z)) | 0.023 | 0.090 | 0.626 |
| 918.398 | 3.275 | Unidentified | 0.034 | 0.112 | 0.623 |
| 385.132 | 16.606 | S-Adenosylhomocysteine | 0.002 | 0.028 | 0.622 |
| 205.028 | 1.227 | 3-phenyllactic acid | 0.036 | 0.115 | 0.618 |
| 436.345 | 21.150 | Unidentified | 0.006 | 0.039 | 0.612 |
| 625.247 | 3.269 | Unidentified | 0.008 | 0.048 | 0.603 |
| 307.109 | 18.676 | Unidentified | 0.042 | 0.125 | 0.602 |
| 326.119 | 7.458 | Citalopram (propionic acid derivative) | 0.014 | 0.066 | 0.598 |
| 534.192 | 4.296 | Unidentified | 0.000 | 0.006 | 0.598 |
| 449.344 | 20.076 | Quercitrin | 0.030 | 0.103 | 0.581 |
| 210.044 | 7.521 | Unidentified | 0.002 | 0.025 | 0.577 |
| 685.284 | 4.297 | gamma-L-Glutamyl-butirosin B | 0.002 | 0.025 | 0.572 |
| 526.428 | 22.055 | Unidentified | 0.006 | 0.040 | 0.571 |
| 430.185 | 1.946 | Unidentified | 0.001 | 0.017 | 0.570 |
| 256.081 | 2.648 | Unidentified | 0.027 | 0.098 | 0.569 |
| 114.091 | 7.095 | Unidentified | 0.007 | 0.042 | 0.564 |
| 227.100 | 1.878 | Unidentified | 0.033 | 0.108 | 0.564 |
| 198.070 | 7.524 | Unidentified | 0.003 | 0.030 | 0.550 |
| 309.226 | 17.938 | Unidentified | 0.000 | 0.007 | 0.548 |
| 369.110 | 4.301 | Unidentified | 0.000 | 0.008 | 0.538 |
| 582.174 | 2.205 | Unidentified | 0.001 | 0.020 | 0.517 |
| 235.130 | 20.218 | 2,6-Diamino-7-hydroxy-azelaic acid | 0.010 | 0.051 | 0.509 |
| 104.106 | 0.826 | 2-Amino-3-methyl-1-butanol | 0.032 | 0.105 | 0.501 |
| 335.108 | 12.123 | (S)-a-Amino-2,5-dihydro-5-oxo-4-isoxazolepropanoic acid N2-glucoside | 0.026 | 0.095 | 0.487 |
| 86.096 | 4.679 | 2-Methylpyrrolidine | 0.000 | 0.007 | 0.466 |
| 111.525 | 5.783 | Unidentified | 0.010 | 0.054 | 0.466 |
| 477.177 | 5.771 | Calcium pantothenate | 0.046 | 0.132 | 0.457 |
| 340.260 | 11.485 | Unidentified | 0.003 | 0.028 | 0.440 |
| 169.035 | 1.719 | Uric acid | 0.041 | 0.122 | 0.436 |
| 514.410 | 21.803 | Sulfolithocholylglycine | 0.003 | 0.028 | 0.428 |
| 249.159 | 8.141 | Unidentified | 0.027 | 0.098 | 0.417 |
| 322.248 | 11.494 | Anandamide (18:3, n-6) | 0.020 | 0.082 | 0.400 |
| 410.105 | 1.745 | 1-(5-Phosphoribosyl)-4-(N-succinocarboxamide)-5-aminoimidazole | 0.018 | 0.077 | 0.373 |
| 195.136 | 18.684 | Unidentified | 0.007 | 0.044 | 0.368 |
| 440.369 | 21.718 | PC(O-12:0/O-1:0) | 0.001 | 0.023 | 0.357 |
| 282.121 | 0.805 | 1-Methyladenosine | 0.048 | 0.135 | 0.356 |
| 442.338 | 19.082 | Unidentified | 0.010 | 0.051 | 0.353 |
| 512.430 | 21.816 | PS(O-18:0/0:0) | 0.042 | 0.125 | 0.348 |
| 221.092 | 6.552 | 5-Hydroxy-L-tryptophan | 0.003 | 0.028 | 0.340 |
| 448.209 | 2.006 | S-Decyl GSH | 0.033 | 0.109 | 0.338 |
| 614.482 | 22.033 | Ferroxamine | 0.014 | 0.066 | 0.334 |
| 542.188 | 4.300 | cyclic adenosine diphosphate ribose | 0.028 | 0.099 | 0.330 |
| 227.173 | 7.008 | 1,8-Diazacyclotetradecane-2,9-dione | 0.011 | 0.057 | 0.328 |
| 442.388 | 21.800 | Leukotriene E3 | 0.013 | 0.063 | 0.328 |
| 293.172 | 1.067 | (E)-2-decylpent-2-enedioic acid | 0.013 | 0.064 | 0.321 |
| 116.071 | 1.672 | L-Proline | 0.024 | 0.091 | 0.303 |
| 746.561 | 21.967 | PC (15:0/18:1(9Z))[U] | 0.003 | 0.028 | 0.301 |
| 118.087 | 1.095 | L-Valine | 0.027 | 0.098 | 0.299 |
| 496.474 | 21.797 | PS (16:0/0:0) | 0.004 | 0.033 | 0.295 |
| 412.377 | 22.049 | PC(O-8:0/2:0) | 0.004 | 0.032 | 0.293 |
| 362.241 | 11.485 | 3-Oxohexadecanoic acid glycerides | 0.004 | 0.033 | 0.287 |
| 72.082 | 1.092 | Unidentified | 0.036 | 0.115 | 0.283 |
| 357.160 | 1.664 | xi-3-Hydroxy-5-phenylpentanoic acid O-beta-D-Glucopyranoside | 0.046 | 0.132 | 0.276 |
| 209.128 | 1.081 | Unidentified | 0.017 | 0.074 | 0.270 |
| 114.046 | 6.538 | Unidentified | 0.007 | 0.042 | 0.265 |
| 70.074 | 1.961 | 1-Pyrroline | 0.001 | 0.023 | 0.241 |
| 103.054 | 6.539 | Unidentified | 0.002 | 0.027 | 0.224 |
| 454.387 | 21.954 | PC(O-12:0/O-2:0) | 0.039 | 0.118 | 0.208 |
| 132.102 | 2.014 | L-Isoleucine | 0.004 | 0.035 | 0.190 |
| 552.531 | 23.282 | Cer(d18:1/17:0) | 0.001 | 0.023 | 0.190 |
| 510.487 | 22.505 | Cer(d18:1/14:0) | 0.007 | 0.042 | 0.176 |
| 431.262 | 6.548 | 17-phenyl trinor PGF2α isopropyl ester | 0.037 | 0.116 | 0.173 |
| 102.047 | 4.300 | 1-Aminocyclopropane-1-carboxylic acid | 0.008 | 0.046 | 0.137 |
| 89.037 | 6.539 | Pyruvate | 0.015 | 0.067 | 0.133 |
| 346.158 | 6.541 | Unidentified | 0.039 | 0.119 | 0.123 |
| 174.111 | 3.299 | Hexanoylglycine | 0.016 | 0.072 | 0.123 |
| 174.092 | 5.029 | Unidentified | 0.044 | 0.128 | 0.108 |
| 212.127 | 3.269 | Unidentified | 0.012 | 0.059 | 0.104 |
| 105.047 | 4.298 | Malonic acid | 0.024 | 0.091 | 0.102 |
| 261.086 | 2.163 | Unidentified | 0.033 | 0.109 | 0.099 |
| 79.022 | 1.135 | Unidentified | 0.038 | 0.117 | 0.097 |
| 119.075 | 4.299 | Methyl-3-hydroxybutyric acid | 0.024 | 0.091 | 0.083 |
| 442.164 | 2.186 | N-(4-hydroxyphenethyl)-2-(2,3,4,9-tetramethyl-7-oxo-7H-furo[2,3-f] chromen-8-yl)acetamide | 0.043 | 0.127 | 0.059 |
| 239.108 | 2.187 | HEPES | 0.027 | 0.098 | -0.072 |
| 152.033 | 1.700 | Unidentified | 0.043 | 0.127 | -0.077 |
| 105.071 | 5.016 | Unidentified | 0.016 | 0.072 | -0.094 |
| 94.042 | 2.222 | Unidentified | 0.031 | 0.104 | -0.106 |
| 182.083 | 2.208 | L-Tyrosine | 0.010 | 0.051 | -0.110 |
| 107.052 | 4.299 | Unidentified | 0.027 | 0.098 | -0.143 |
| 189.071 | 16.249 | Unidentified | 0.042 | 0.125 | -0.150 |
| 103.055 | 2.223 | Unidentified | 0.029 | 0.102 | -0.153 |
| 549.157 | 4.298 | Daidzein 7-O-glucoside-4'-O-apioside | 0.043 | 0.127 | -0.160 |
| 165.055 | 2.221 | Unidentified | 0.006 | 0.038 | -0.161 |
| 109.064 | 2.220 | Unidentified | 0.001 | 0.018 | -0.163 |
| 150.058 | 1.281 | L-Methionine | 0.006 | 0.038 | -0.165 |
| 133.034 | 1.283 | Unidentified | 0.003 | 0.029 | -0.169 |
| 107.052 | 2.221 | Unidentified | 0.002 | 0.026 | -0.172 |
| 95.050 | 2.221 | Phenol | 0.000 | 0.014 | -0.177 |
| 134.060 | 1.848 | Indoxyl | 0.047 | 0.133 | -0.178 |
| 152.055 | 1.279 | Guanine | 0.001 | 0.015 | -0.178 |
| 123.046 | 2.221 | THIODIGLYCOL | 0.002 | 0.027 | -0.179 |
| 643.225 | 19.440 | Unidentified | 0.017 | 0.074 | -0.179 |
| 98.962 | 19.790 | Phosphoric acid | 0.033 | 0.109 | -0.180 |
| 153.075 | 1.844 | Ribitol | 0.036 | 0.115 | -0.182 |
| 136.078 | 2.221 | 2-Phenylacetamide | 0.001 | 0.023 | -0.185 |
| 106.066 | 1.848 | 3-Methylthiopropanamine | 0.004 | 0.033 | -0.191 |
| 338.343 | 21.906 | Unidentified | 0.049 | 0.135 | -0.197 |
| 119.048 | 2.221 | Allophanic acid methyl ester | 0.003 | 0.029 | -0.198 |
| 136.077 | 16.229 | N-Acetylarylamine | 0.031 | 0.105 | -0.199 |
| 458.692 | 17.498 | 7-methylguanosine 5'-diphosphate | 0.006 | 0.039 | -0.200 |
| 121.067 | 2.224 | 4-deoxy-threonic acid | 0.002 | 0.023 | -0.204 |
| 124.074 | 5.778 | Unidentified | 0.017 | 0.074 | -0.207 |
| 104.054 | 1.283 | Biuret | 0.005 | 0.035 | -0.208 |
| 603.102 | 0.831 | Unidentified | 0.014 | 0.066 | -0.212 |
| 147.046 | 2.223 | Coumaric acid | 0.002 | 0.025 | -0.224 |
| 91.056 | 2.221 | Unidentified | 0.001 | 0.018 | -0.224 |
| 117.058 | 2.220 | Ketoisovaleric acid | 0.017 | 0.074 | -0.225 |
| 80.048 | 1.845 | Unidentified | 0.004 | 0.033 | -0.225 |
| 597.140 | 2.214 | Unidentified | 0.046 | 0.132 | -0.228 |
| 375.097 | 16.253 | Unidentified | 0.022 | 0.087 | -0.230 |
| 263.122 | 16.226 | Nopalinic acid | 0.050 | 0.137 | -0.240 |
| 365.101 | 0.961 | Unidentified | 0.027 | 0.098 | -0.240 |
| 319.035 | 5.778 | Unidentified | 0.024 | 0.091 | -0.242 |
| 473.177 | 2.162 | C1'-C9-Glycosylated UWM6 | 0.001 | 0.018 | -0.243 |
| 353.113 | 16.244 | Penicilloic G acid | 0.047 | 0.132 | -0.245 |
| 203.052 | 1.032 | Unidentified | 0.012 | 0.061 | -0.251 |
| 100.037 | 15.369 | Succinimide | 0.030 | 0.104 | -0.255 |
| 232.118 | 1.068 | Suberylglycine | 0.021 | 0.086 | -0.255 |
| 102.056 | 1.283 | Unidentified | 0.002 | 0.025 | -0.258 |
| 261.023 | 18.642 | D-Galactose 6-sulfate | 0.029 | 0.102 | -0.264 |
| 454.113 | 9.043 | Unidentified | 0.037 | 0.115 | -0.270 |
| 340.089 | 4.301 | 6-Hydroxy-5-methoxyindole glucuronide | 0.028 | 0.100 | -0.271 |
| 145.048 | 4.997 | 3-hexenedioic acid | 0.047 | 0.133 | -0.272 |
| 284.110 | 1.362 | Glutamyl-Histidine | 0.016 | 0.071 | -0.274 |
| 256.081 | 0.791 | methyl 2-[(2,3-dihydroxybenzoyl) amino]-3-hydroxypropanoate | 0.029 | 0.102 | -0.280 |
| 569.208 | 0.785 | Leukotriene F4 | 0.001 | 0.023 | -0.280 |
| 307.108 | 16.256 | Glutathione amide | 0.005 | 0.035 | -0.283 |
| 355.066 | 18.653 | Phenolsulfonphthalein | 0.014 | 0.065 | -0.284 |
| 102.057 | 9.879 | Unidentified | 0.034 | 0.111 | -0.285 |
| 134.998 | 1.192 | Malic acid | 0.003 | 0.028 | -0.296 |
| 180.087 | 6.618 | 3-Hydroxymethylglutaric acid | 0.046 | 0.131 | -0.300 |
| 543.154 | 0.745 | Unidentified | 0.003 | 0.028 | -0.300 |
| 272.085 | 18.623 | Unidentified | 0.012 | 0.061 | -0.303 |
| 687.212 | 19.401 | Unidentified | 0.033 | 0.109 | -0.306 |
| 388.158 | 1.269 | Unidentified | 0.030 | 0.104 | -0.307 |
| 105.000 | 1.281 | β-Hydroxypyruvic acid | 0.001 | 0.019 | -0.312 |
| 130.048 | 9.874 | Pyroglutamic acid | 0.034 | 0.111 | -0.313 |
| 130.202 | 1.696 | Octylamine | 0.006 | 0.039 | -0.317 |
| 257.095 | 18.646 | Unidentified | 0.007 | 0.044 | -0.318 |
| 156.101 | 1.395 | Unidentified | 0.012 | 0.059 | -0.320 |
| 308.169 | 5.486 | Unidentified | 0.048 | 0.133 | -0.324 |
| 516.224 | 1.070 | Unidentified | 0.019 | 0.079 | -0.328 |
| 191.082 | 16.230 | 2-Oxo-7-methylthioheptanoic acid | 0.001 | 0.023 | -0.331 |
| 284.058 | 16.265 | N2-Acetyl-L-aminoadipyl-δ-phosphate | 0.008 | 0.046 | -0.358 |
| 142.086 | 1.408 | Unidentified | 0.002 | 0.024 | -0.371 |
| 601.108 | 0.825 | Protoporphyrin IX | 0.021 | 0.086 | -0.372 |
| 74.060 | 1.290 | Unidentified | 0.008 | 0.048 | -0.373 |
| 202.108 | 1.394 | (E)-1-(4-methylquinazolin-2(1H)-ylidene) guanidine | 0.000 | 0.011 | -0.375 |
| 302.119 | 1.350 | Unidentified | 0.013 | 0.064 | -0.376 |
| 127.038 | 1.410 | Unidentified | 0.002 | 0.027 | -0.381 |
| 114.091 | 1.402 | Unidentified | 0.014 | 0.066 | -0.383 |
| 180.101 | 1.403 | Unidentified | 0.017 | 0.074 | -0.390 |
| 190.991 | 1.143 | Quinol sulfate | 0.037 | 0.115 | -0.392 |
| 211.167 | 19.450 | 3E,5E-tridecadienoic acid | 0.007 | 0.042 | -0.393 |
| 369.075 | 19.950 | 2-Deoxy-4-O-[(2E)-3-(4-hydroxyphenyl)-2-propenoyl]-3-C-(methoxycarbonyl)pentaric acid | 0.014 | 0.066 | -0.395 |
| 258.111 | 1.797 | Glycerophosphocholine | 0.048 | 0.134 | -0.398 |
| 778.209 | 2.210 | PS (14:1(9Z)/22:6(4Z,7Z,10Z,13Z,16Z,19Z)) | 0.024 | 0.091 | -0.398 |
| 273.089 | 18.650 | Unidentified | 0.001 | 0.014 | -0.401 |
| 354.147 | 11.344 | Unidentified | 0.022 | 0.088 | -0.410 |
| 275.138 | 16.232 | Glutaminyl-Glutamine | 0.002 | 0.026 | -0.418 |
| 139.004 | 1.562 | Unidentified | 0.009 | 0.050 | -0.422 |
| 280.139 | 1.403 | Unidentified | 0.006 | 0.039 | -0.429 |
| 196.099 | 1.414 | L-Tyrosine methyl ester | 0.023 | 0.090 | -0.432 |
| 226.506 | 18.662 | Unidentified | 0.047 | 0.133 | -0.438 |
| 151.062 | 5.364 | 1-Methylhypoxanthine | 0.022 | 0.087 | -0.441 |
| 160.095 | 1.401 | N-Isovalerylglycine | 0.010 | 0.054 | -0.443 |
| 152.118 | 1.842 | Unidentified | 0.039 | 0.119 | -0.447 |
| 167.094 | 1.133 | N-Formyl-4-amino-5-aminomethyl-2-methylpyrimidine | 0.012 | 0.058 | -0.453 |
| 244.121 | 1.406 | Glutaminyl-Proline | 0.022 | 0.089 | -0.457 |
| 84.043 | 4.709 | 1-Methylpyrrolinium | 0.002 | 0.026 | -0.458 |
| 173.960 | 2.494 | Unidentified | 0.049 | 0.136 | -0.464 |
| 560.189 | 1.070 | Unidentified | 0.042 | 0.125 | -0.472 |
| 228.195 | 19.443 | Unidentified | 0.021 | 0.085 | -0.479 |
| 161.068 | 1.405 | Unidentified | 0.004 | 0.033 | -0.480 |
| 262.130 | 1.401 | Unidentified | 0.005 | 0.036 | -0.492 |
| 216.124 | 1.399 | Unidentified | 0.003 | 0.029 | -0.497 |
| 106.041 | 9.874 | 2-cyano-Pyrimidine | 0.039 | 0.118 | -0.498 |
| 194.023 | 1.263 | Unidentified | 0.005 | 0.038 | -0.499 |
| 234.135 | 1.398 | Unidentified | 0.003 | 0.029 | -0.511 |
| 312.132 | 6.618 | N2, N2-Dimethylguanosine | 0.047 | 0.133 | -0.512 |
| 198.113 | 1.405 | Metanephrine | 0.000 | 0.014 | -0.512 |
| 247.128 | 4.380 | L-beta-aspartyl-L-leucine | 0.002 | 0.023 | -0.514 |
| 524.077 | 1.377 | Unidentified | 0.028 | 0.099 | -0.515 |
| 245.094 | 18.633 | D-Biotin | 0.022 | 0.087 | -0.516 |
| 492.139 | 5.771 | Unidentified | 0.009 | 0.050 | -0.518 |
| 228.085 | 4.069 | L-Arogenate | 0.003 | 0.028 | -0.521 |
| 268.120 | 4.872 | Unidentified | 0.004 | 0.032 | -0.522 |
| 547.230 | 0.780 | Unidentified | 0.009 | 0.049 | -0.530 |
| 384.022 | 18.618 | Unidentified | 0.008 | 0.046 | -0.531 |
| 182.210 | 2.216 | Unidentified | 0.000 | 0.007 | -0.533 |
| 175.148 | 19.447 | Unidentified | 0.018 | 0.078 | -0.538 |
| 518.237 | 1.366 | PS (18:4(6Z,9Z,12Z,15Z)/0:0) | 0.009 | 0.049 | -0.538 |
| 460.151 | 1.038 | Phe-Trp-OH | 0.038 | 0.117 | -0.538 |
| 582.165 | 1.068 | Unidentified | 0.050 | 0.137 | -0.540 |
| 204.123 | 12.327 | Acetylcarnitine | 0.024 | 0.091 | -0.547 |
| 176.055 | 9.874 | N-Formyl-L-glutamate | 0.009 | 0.050 | -0.556 |
| 172.042 | 1.275 | Unidentified | 0.008 | 0.046 | -0.557 |
| 90.054 | 2.340 | Alanine | 0.031 | 0.104 | -0.557 |
| 613.188 | 1.398 | Unidentified | 0.028 | 0.099 | -0.566 |
| 354.075 | 4.780 | Unidentified | 0.011 | 0.054 | -0.571 |
| 758.136 | 16.238 | Peonidin 3-sambubioside 5-glucoside | 0.000 | 0.006 | -0.580 |
| 223.076 | 4.779 | L-Cystathionine | 0.012 | 0.059 | -0.583 |
| 438.304 | 19.409 | PE(P-16:0/0:0) | 0.005 | 0.036 | -0.584 |
| 353.315 | 16.232 | PGE2 | 0.042 | 0.125 | -0.585 |
| 334.975 | 1.198 | Unidentified | 0.006 | 0.040 | -0.591 |
| 256.082 | 1.914 | Nicotinate D-ribonucleoside | 0.001 | 0.015 | -0.605 |
| 238.071 | 1.920 | Unidentified | 0.002 | 0.028 | -0.611 |
| 192.054 | 9.894 | Unidentified | 0.024 | 0.091 | -0.612 |
| 383.026 | 18.621 | Unidentified | 0.021 | 0.085 | -0.623 |
| 148.053 | 9.879 | L-Glutamate | 0.027 | 0.098 | -0.627 |
| 123.107 | 2.228 | Unidentified | 0.000 | 0.006 | -0.631 |
| 438.173 | 1.023 | Unidentified | 0.028 | 0.100 | -0.642 |
| 158.115 | 12.322 | Unidentified | 0.025 | 0.095 | -0.644 |
| 393.071 | 0.963 | 5,7,3',4',5'-Pentahydroxy-3,6,8-trimethoxyflavone | 0.009 | 0.049 | -0.668 |
| 736.214 | 4.295 | Unidentified | 0.025 | 0.094 | -0.673 |
| 146.028 | 1.178 | Unidentified | 0.029 | 0.101 | -0.685 |
| 163.089 | 4.132 | Unidentified | 0.009 | 0.050 | -0.690 |
| 283.069 | 0.720 | 9-Riburonosylhypoxanthine | 0.045 | 0.131 | -0.695 |
| 254.099 | 8.874 | Dihydroferuloylglycine | 0.000 | 0.002 | -0.697 |
| 240.230 | 19.002 | Unidentified | 0.041 | 0.122 | -0.700 |
| 208.099 | 8.873 | 4-(2-Aminophenyl)-2,4-dioxobutanoic acid | 0.000 | 0.013 | -0.716 |
| 158.155 | 19.484 | Unidentified | 0.003 | 0.028 | -0.723 |
| 280.231 | 1.408 | Linoleamide | 0.006 | 0.038 | -0.743 |
| 277.120 | 15.210 | Unidentified | 0.038 | 0.117 | -0.747 |
| 130.064 | 15.207 | 3-Methylene-indolenine | 0.036 | 0.115 | -0.762 |
| 386.212 | 19.422 | Unidentified | 0.005 | 0.038 | -0.784 |
| 267.096 | 9.892 | Unidentified | 0.005 | 0.037 | -0.787 |
| 626.372 | 18.951 | Unidentified | 0.039 | 0.118 | -0.787 |
| 197.102 | 2.896 | L-(+)-Arginine | 0.009 | 0.049 | -0.791 |
| 612.187 | 1.396 | Unidentified | 0.007 | 0.042 | -0.802 |
| 148.132 | 0.975 | Unidentified | 0.020 | 0.082 | -0.815 |
| 687.285 | 19.381 | Unidentified | 0.021 | 0.085 | -0.822 |
| 262.214 | 1.401 | Unidentified | 0.007 | 0.041 | -0.822 |
| 94.065 | 4.138 | Unidentified | 0.008 | 0.047 | -0.823 |
| 582.231 | 2.702 | Unidentified | 0.009 | 0.049 | -0.824 |
| 714.235 | 4.297 | PS (13:0/18:4(6Z,9Z,12Z,15Z)) | 0.037 | 0.116 | -0.834 |
| 199.132 | 18.942 | Unidentified | 0.032 | 0.105 | -0.836 |
| 581.256 | 1.371 | Unidentified | 0.014 | 0.065 | -0.839 |
| 758.452 | 19.030 | PE (18:4(6Z,9Z,12Z,15Z)/20:5(5Z,8Z,11Z,14Z,17Z)) | 0.006 | 0.039 | -0.843 |
| 454.105 | 17.750 | Unidentified | 0.000 | 0.006 | -0.848 |
| 575.271 | 0.770 | Tetrahydrofolyl-[Glu](2) | 0.038 | 0.117 | -0.852 |
| 292.551 | 19.530 | Unidentified | 0.024 | 0.091 | -0.853 |
| 146.062 | 4.136 | Unidentified | 0.012 | 0.061 | -0.875 |
| 483.040 | 1.184 | Thymidine 5'-triphosphate | 0.002 | 0.027 | -0.878 |
| 355.118 | 18.623 | Unidentified | 0.024 | 0.091 | -0.889 |
| 515.172 | 15.768 | TyrMe-Nap-OH | 0.024 | 0.091 | -0.893 |
| 314.084 | 1.945 | Unidentified | 0.000 | 0.005 | -0.894 |
| 192.064 | 4.132 | 5-Hydroxyindoleacetic acid | 0.005 | 0.036 | -0.909 |
| 670.398 | 18.996 | PE (13:0/18:4(6Z,9Z,12Z,15Z)) | 0.000 | 0.007 | -0.941 |
| 281.210 | 1.404 | 12S-hydroxy-5Z,8E,10E-heptadecatrienoic acid | 0.006 | 0.038 | -0.942 |
| 330.058 | 1.959 | cAMP | 0.004 | 0.033 | -0.942 |
| 334.140 | 10.201 | Unidentified | 0.036 | 0.114 | -0.960 |
| 274.090 | 1.944 | Pentose + Proline | 0.000 | 0.007 | -0.970 |
| 136.078 | 4.131 | L-Homocysteine | 0.008 | 0.048 | -0.984 |
| 423.194 | 1.972 | Unidentified | 0.000 | 0.011 | -0.995 |
| 270.314 | 19.617 | Unidentified | 0.046 | 0.132 | -1.010 |
| 552.182 | 1.974 | N-Acetyl-N6,N6,O-tridemethylpuromycin-5'-phosphate | 0.002 | 0.027 | -1.013 |
| 209.093 | 4.132 | Kynurenine | 0.006 | 0.039 | -1.025 |
| 261.146 | 7.254 | L-gamma-glutamyl-L-isoleucine | 0.005 | 0.038 | -1.041 |
| 220.096 | 14.010 | Unidentified | 0.010 | 0.051 | -1.065 |
| 280.312 | 1.409 | Unidentified | 0.004 | 0.033 | -1.089 |
| 262.321 | 1.402 | Unidentified | 0.004 | 0.035 | -1.154 |
| 77.039 | 14.015 | Unidentified | 0.006 | 0.039 | -1.161 |
| 303.068 | 5.293 | Unidentified | 0.004 | 0.033 | -1.261 |
| 642.366 | 18.625 | Unidentified | 0.018 | 0.076 | -1.272 |
| 575.129 | 14.016 | Unidentified | 0.020 | 0.083 | -1.275 |
| 891.311 | 1.402 | dipalmitoyl phosphatidylinositol 3-phosphate | 0.003 | 0.030 | -1.291 |
| 295.129 | 8.362 | Glutamylphenylalanine | 0.006 | 0.040 | -1.297 |
| 277.120 | 12.502 | Unidentified | 0.003 | 0.029 | -1.297 |
| 630.402 | 19.036 | Unidentified | 0.002 | 0.026 | -1.322 |
| 586.377 | 19.016 | Unidentified | 0.008 | 0.046 | -1.330 |
| 490.210 | 0.770 | Unidentified | 0.001 | 0.020 | -1.353 |
| 103.054 | 14.021 | Unidentified | 0.004 | 0.032 | -1.397 |
| 542.357 | 18.984 | PC(20:5(5Z,8Z,11Z,14Z,17Z)/0:0) | 0.000 | 0.013 | -1.502 |
| 271.275 | 19.000 | 2-keto palmitic acid | 0.035 | 0.114 | -1.539 |
| 605.175 | 1.935 | SP1 | 0.000 | 0.007 | -1.600 |
| 674.429 | 19.078 | Unidentified | 0.002 | 0.027 | -1.619 |
| 337.021 | 14.018 | Unidentified | 0.000 | 0.009 | -1.630 |
| 166.086 | 14.016 | L-Phenylalanine | 0.001 | 0.023 | -1.684 |
| 504.176 | 1.260 | TyrMe-Trp-OH | 0.000 | 0.013 | -1.716 |
| 120.079 | 14.016 | L-Threonine | 0.002 | 0.023 | -1.738 |
| 192.101 | 14.021 | Unidentified | 0.003 | 0.028 | -1.793 |
| 113.107 | 5.786 | 2-Imino-4-methylpiperidine | 0.000 | 0.007 | -1.808 |
| 238.108 | 14.016 | N-lactoyl-phenylalanine | 0.001 | 0.023 | -1.827 |
| 113.108 | 3.814 | Hydroxymethylphosphonate | 0.000 | 0.005 | -1.857 |
| 260.091 | 14.014 | Unidentified | 0.003 | 0.029 | -2.154 |
| 528.120 | 14.018 | Unidentified | 0.002 | 0.023 | -2.809 |

**Table S6.** Significantly Altered Metabolites in SAECs Exposed to 10 µg/mL PM Collected from the Printer Room During Printing with PLA Filaments.

| m/z | rt | Metabolite Name | p.value | FDR | log2(FC) |
| --- | --- | --- | --- | --- | --- |
| 412.320 | 19.839 | Unidentified | 0.029 | 0.103 | 5.643 |
| 786.602 | 21.302 | PC (18:1(9Z)/18:1(9Z))[S] | 0.000 | 0.008 | 4.577 |
| 387.177 | 19.739 | 3,5-Di-O-methyl-8-prenylafzelechin-4beta-ol | 0.002 | 0.026 | 4.386 |
| 404.203 | 19.741 | 16-phenoxy tetranor PGF2α methyl amide | 0.003 | 0.031 | 4.199 |
| 87.042 | 12.443 | 4-Deoxytetronic acid | 0.000 | 0.013 | 4.141 |
| 432.238 | 19.741 | 17-phenoxy trinor PGF2α ethyl amide | 0.002 | 0.026 | 3.775 |
| 766.535 | 21.462 | PC (15:0/20:5(5Z,8Z,11Z,14Z,17Z)) | 0.001 | 0.016 | 3.763 |
| 284.209 | 13.296 | Unidentified | 0.000 | 0.001 | 3.760 |
| 267.181 | 13.297 | Unidentified | 0.000 | 0.000 | 3.643 |
| 289.161 | 13.294 | Arginyl-Asparagine | 0.000 | 0.004 | 3.453 |
| 103.075 | 13.294 | Isovaleric acid | 0.000 | 0.004 | 3.317 |
| 268.102 | 3.877 | Adenosine | 0.017 | 0.077 | 3.174 |
| 298.093 | 7.494 | 5'-S-Methylthioadenosine; LC-ESI-QTOF; MS2; CE | 0.013 | 0.068 | 3.113 |
| 105.071 | 19.736 | L-2,3-DIAMINOPROPIONIC ACID | 0.001 | 0.020 | 3.021 |
| 110.036 | 2.300 | Hypotaurine | 0.000 | 0.004 | 2.639 |
| 546.398 | 21.549 | LysoPC(20:3(5Z,8Z,11Z)) | 0.000 | 0.004 | 2.574 |
| 590.426 | 21.548 | CerP(d18:1/14:0) | 0.000 | 0.013 | 2.569 |
| 240.182 | 11.679 | 2-Octyl-4-propylthiazole | 0.001 | 0.013 | 2.541 |
| 223.153 | 11.679 | Unidentified | 0.001 | 0.013 | 2.486 |
| 493.198 | 2.561 | Unidentified | 0.001 | 0.016 | 2.463 |
| 159.025 | 1.820 | fumarylacetic acid | 0.000 | 0.008 | 2.419 |
| 268.209 | 11.683 | (2R,5S)-2,5-di((E)-pent-2-en-4-yn-1-yl) decahydroquinoline | 0.000 | 0.011 | 2.412 |
| 245.136 | 11.679 | Polyethylene, oxidized | 0.001 | 0.016 | 2.367 |
| 652.412 | 17.163 | PS (14:0/12:0) | 0.005 | 0.042 | 2.365 |
| 163.110 | 14.364 | 2-Hydroxyadipic acid | 0.001 | 0.018 | 2.198 |
| 137.047 | 1.820 | Hypoxanthine | 0.001 | 0.013 | 2.189 |
| 147.103 | 11.681 | 2-hydroxy enanthoic acid | 0.000 | 0.013 | 2.130 |
| 186.074 | 2.569 | Unidentified | 0.000 | 0.009 | 2.109 |
| 589.149 | 1.101 | 25-hydroxyvitamin D2 25-(β-glucuronide) / 25 hydroxyergocalciferol 25-(β-glucuronide) | 0.000 | 0.006 | 2.097 |
| 236.112 | 2.569 | N-(9-oxodecyl) acetamide | 0.000 | 0.004 | 1.867 |
| 573.209 | 16.361 | Unidentified | 0.024 | 0.093 | 1.857 |
| 153.041 | 2.293 | Xanthine | 0.000 | 0.008 | 1.824 |
| 384.115 | 6.653 | Succinoadenosine | 0.012 | 0.064 | 1.740 |
| 247.138 | 1.632 | L-N2-(2-Carboxyethyl) arginine | 0.003 | 0.032 | 1.701 |
| 258.094 | 2.561 | Unidentified | 0.001 | 0.020 | 1.696 |
| 110.974 | 1.231 | Unidentified | 0.019 | 0.083 | 1.621 |
| 464.356 | 22.202 | D-Glucosyldihydrosphingosine | 0.011 | 0.061 | 1.608 |
| 574.434 | 21.550 | PS (22:4(7Z,10Z,13Z,16Z)/0:0) | 0.002 | 0.024 | 1.549 |
| 205.028 | 1.227 | 3-phenyllactic acid | 0.048 | 0.128 | 1.478 |
| 168.065 | 1.436 | Pyridoxal (Vitamin B6) | 0.012 | 0.064 | 1.439 |
| 530.404 | 21.559 | Unidentified | 0.000 | 0.004 | 1.422 |
| 324.131 | 1.430 | Unidentified | 0.002 | 0.026 | 1.272 |
| 492.388 | 22.203 | Glutathionylspermine | 0.025 | 0.095 | 1.248 |
| 278.065 | 1.744 | 2-Phthalimidoglutaric acid | 0.017 | 0.078 | 1.245 |
| 381.337 | 22.291 | 15-oxo-18Z-tetracosenoic acid | 0.023 | 0.091 | 1.229 |
| 520.332 | 14.860 | 1-Linoleoylglycerophosphocholine | 0.006 | 0.045 | 1.225 |
| 161.960 | 1.211 | Unidentified | 0.049 | 0.130 | 1.224 |
| 165.112 | 6.665 | Unidentified | 0.000 | 0.006 | 1.210 |
| 482.422 | 22.119 | Ceramide (d18:1/12:0) | 0.004 | 0.035 | 1.210 |
| 564.362 | 15.690 | PC (18:1(9E)/2:0) | 0.005 | 0.039 | 1.166 |
| 460.270 | 20.183 | 17-phenyl trinor Prostaglandin E2 serinol amide | 0.035 | 0.111 | 1.162 |
| 306.115 | 1.430 | Unidentified | 0.003 | 0.030 | 1.157 |
| 319.072 | 1.814 | Melanin | 0.041 | 0.121 | 1.150 |
| 176.119 | 4.799 | 7-Methylthioheptanaldoxime | 0.000 | 0.004 | 1.150 |
| 187.094 | 6.665 | Unidentified | 0.002 | 0.024 | 1.127 |
| 412.095 | 1.755 | Unidentified | 0.041 | 0.122 | 1.118 |
| 241.078 | 1.632 | Unidentified | 0.002 | 0.021 | 1.092 |
| 167.998 | 1.802 | 2,3-Pyridinedicarboxylic acid | 0.047 | 0.128 | 1.091 |
| 426.079 | 1.749 | Hyaluronic acid | 0.028 | 0.101 | 1.089 |
| 297.038 | 1.765 | Unidentified | 0.026 | 0.097 | 1.085 |
| 104.106 | 0.826 | 2-Amino-3-methyl-1-butanol | 0.023 | 0.090 | 1.084 |
| 298.117 | 5.348 | 1-Methylguanosine | 0.008 | 0.053 | 1.082 |
| 206.079 | 13.455 | Indolelactic acid | 0.046 | 0.127 | 1.076 |
| 113.034 | 2.589 | Uracil | 0.000 | 0.010 | 1.065 |
| 335.108 | 12.123 | (S)-a-Amino-2,5-dihydro-5-oxo-4-isoxazolepropanoic acid N2-glucoside | 0.003 | 0.033 | 1.049 |
| 663.444 | 22.730 | Prostaglandin E2-biotin | 0.004 | 0.033 | 1.036 |
| 555.127 | 1.776 | 3-O-(6-O-alpha-D-Xylosylphospho-alpha-D-mannopyranosyl)-alpha-D-mannopyranose | 0.031 | 0.106 | 1.031 |
| 541.133 | 1.751 | Unidentified | 0.047 | 0.128 | 1.013 |
| 476.309 | 13.949 | N-docosahexaenoyl phenylalanine | 0.008 | 0.055 | 0.988 |
| 426.393 | 22.298 | Vaccenyl carnitine | 0.036 | 0.113 | 0.983 |
| 111.011 | 4.001 | Pyruvic acid | 0.013 | 0.068 | 0.965 |
| 371.224 | 11.839 | 17-phenyl trinor-13,14-dihydro Prostaglandin A2 | 0.001 | 0.013 | 0.964 |
| 84.043 | 4.709 | 1-Methylpyrrolinium | 0.010 | 0.058 | 0.956 |
| 388.253 | 11.845 | 17-phenyl trinor PGF2α amide | 0.005 | 0.042 | 0.951 |
| 342.208 | 0.789 | PC(O-6:0/0:0) [U] | 0.010 | 0.058 | 0.945 |
| 209.137 | 8.613 | 2,4-Diphenyl-1-butene | 0.006 | 0.045 | 0.921 |
| 268.136 | 11.140 | Unidentified | 0.012 | 0.064 | 0.918 |
| 187.032 | 18.681 | Unidentified | 0.005 | 0.043 | 0.897 |
| 680.472 | 22.775 | PS(O-16:0/13:0) | 0.021 | 0.086 | 0.866 |
| 496.474 | 21.797 | PS (16:0/0:0) | 0.037 | 0.116 | 0.849 |
| 436.345 | 21.150 | Unidentified | 0.021 | 0.087 | 0.845 |
| 708.511 | 22.725 | PS(O-16:0/15:0) | 0.012 | 0.064 | 0.843 |
| 169.035 | 1.719 | Uric acid | 0.012 | 0.064 | 0.830 |
| 210.044 | 7.521 | Unidentified | 0.013 | 0.068 | 0.794 |
| 195.136 | 18.684 | Unidentified | 0.026 | 0.095 | 0.792 |
| 242.062 | 7.529 | N-(2,3-Dihydroxybenzoyl)-L-serine | 0.025 | 0.094 | 0.782 |
| 198.070 | 7.524 | Unidentified | 0.015 | 0.074 | 0.768 |
| 309.226 | 17.938 | Unidentified | 0.039 | 0.118 | 0.768 |
| 512.430 | 21.816 | PS(O-18:0/0:0) | 0.008 | 0.054 | 0.754 |
| 232.155 | 5.597 | Butyryl-L-carnitine | 0.009 | 0.055 | 0.748 |
| 514.410 | 21.803 | Sulfolithocholylglycine | 0.031 | 0.106 | 0.745 |
| 394.350 | 22.080 | N-palmitoyl histidine | 0.025 | 0.094 | 0.731 |
| 227.100 | 1.878 | Unidentified | 0.000 | 0.013 | 0.725 |
| 301.145 | 2.018 | Unidentified | 0.000 | 0.013 | 0.725 |
| 414.359 | 21.464 | Heptadecanoyl carnitine | 0.047 | 0.128 | 0.719 |
| 628.504 | 22.394 | Cer(t18:0/20:0(2OH)) | 0.027 | 0.099 | 0.718 |
| 658.502 | 22.003 | PE (12:0/18:3(6Z,9Z,12Z)) | 0.020 | 0.085 | 0.716 |
| 453.339 | 14.548 | (17E)-1α,25-dihydroxy-26,27-dimethyl-17,20,22,22,23,23-hexadehydro-24a-homovitamin D3 / (17E)-1α,25-dihydroxy-26,27-dimethyl-17,20,22,22,23,23-hexadehydro-24a-homocholecalciferol | 0.009 | 0.055 | 0.715 |
| 367.266 | 18.648 | Unidentified | 0.022 | 0.090 | 0.714 |
| 237.085 | 4.957 | N, N-Dihydroxy-L-tryptophan | 0.002 | 0.022 | 0.705 |
| 352.095 | 5.055 | Unidentified | 0.006 | 0.045 | 0.693 |
| 481.036 | 1.715 | Unidentified | 0.033 | 0.108 | 0.691 |
| 442.388 | 21.800 | Leukotriene E3 | 0.031 | 0.106 | 0.667 |
| 217.070 | 0.978 | 1,3,7-trimethyl-3,7-dihydro-1H-purine-2,6-dione | 0.003 | 0.033 | 0.667 |
| 584.185 | 1.227 | Unidentified | 0.038 | 0.117 | 0.661 |
| 548.170 | 2.168 | Unidentified | 0.003 | 0.030 | 0.656 |
| 412.377 | 22.049 | PC(O-8:0/2:0) | 0.009 | 0.055 | 0.640 |
| 452.390 | 22.438 | PC(P-14:0/0:0) | 0.047 | 0.128 | 0.635 |
| 378.356 | 22.069 | N-arachidonoyl dihydroxypropylamine | 0.042 | 0.122 | 0.630 |
| 708.576 | 22.743 | PS(O-16:0/O-16:0) [U] | 0.002 | 0.024 | 0.630 |
| 157.044 | 7.526 | Unidentified | 0.045 | 0.126 | 0.621 |
| 520.199 | 4.299 | Unidentified | 0.004 | 0.033 | 0.620 |
| 401.103 | 2.205 | S-Acetylphosphopantetheine | 0.001 | 0.016 | 0.608 |
| 672.531 | 22.365 | GlcCer(d18:1/14:0) | 0.037 | 0.115 | 0.604 |
| 407.157 | 0.766 | Unidentified | 0.003 | 0.033 | 0.600 |
| 406.242 | 20.519 | 4-hydroxy-3,3,5-trimethyl-4-[(E)-3-[3,4,5-trihydroxy-6-(hydroxymethyl)oxan-2-yl]oxybut-1-enyl]cyclohexan-1-one | 0.048 | 0.128 | 0.597 |
| 418.252 | 1.990 | 16-phenoxy Prostaglandin F2a ethyl amide | 0.037 | 0.115 | 0.575 |
| 736.536 | 22.760 | PS(O-18:0/15:0) | 0.021 | 0.087 | 0.568 |
| 454.387 | 21.954 | PC(O-12:0/O-2:0) | 0.040 | 0.121 | 0.562 |
| 113.962 | 3.978 | Unidentified | 0.042 | 0.122 | 0.562 |
| 482.401 | 22.062 | PC(O-15:0/O-1:0) [U] | 0.038 | 0.116 | 0.560 |
| 614.482 | 22.033 | Ferroxamine | 0.012 | 0.066 | 0.557 |
| 235.130 | 20.218 | 2,6-Diamino-7-hydroxy-azelaic acid | 0.033 | 0.108 | 0.556 |
| 549.343 | 1.992 | 5beta-scymnol sulfate | 0.010 | 0.060 | 0.552 |
| 685.284 | 4.297 | gamma-L-Glutamyl-butirosin B | 0.000 | 0.011 | 0.540 |
| 86.096 | 4.679 | 2-Methylpyrrolidine | 0.000 | 0.006 | 0.517 |
| 369.110 | 4.301 | Unidentified | 0.034 | 0.110 | 0.515 |
| 391.284 | 21.148 | 3α-Hydroxy-6-oxo-5β-cholan-24-oic Acid | 0.046 | 0.127 | 0.514 |
| 226.179 | 18.958 | (8S, Z)-6-((S)-3-hydroxy-2-methylpropylidene)-8-methyloctahydroindolizin-8-ol | 0.019 | 0.083 | 0.512 |
| 384.343 | 22.737 | N-stearoyl valine | 0.043 | 0.123 | 0.504 |
| 582.174 | 2.205 | Unidentified | 0.001 | 0.016 | 0.489 |
| 282.121 | 0.805 | 1-Methyladenosine | 0.045 | 0.126 | 0.480 |
| 410.105 | 1.745 | 1-(5-Phosphoribosyl)-4-(N-succinocarboxamide)-5-aminoimidazole | 0.030 | 0.106 | 0.477 |
| 746.561 | 21.967 | PC (15:0/18:1(9Z))[U] | 0.046 | 0.127 | 0.474 |
| 449.344 | 20.076 | Quercitrin | 0.047 | 0.128 | 0.450 |
| 221.092 | 6.552 | 5-Hydroxy-L-tryptophan | 0.001 | 0.016 | 0.446 |
| 442.338 | 19.082 | Unidentified | 0.013 | 0.068 | 0.446 |
| 317.118 | 2.000 | Unidentified | 0.017 | 0.078 | 0.443 |
| 534.192 | 4.296 | Unidentified | 0.004 | 0.033 | 0.440 |
| 448.209 | 2.006 | S-Decyl GSH | 0.019 | 0.081 | 0.433 |
| 118.087 | 1.095 | L-Valine | 0.006 | 0.043 | 0.429 |
| 72.082 | 1.092 | Unidentified | 0.001 | 0.019 | 0.419 |
| 116.071 | 1.672 | L-Proline | 0.029 | 0.104 | 0.414 |
| 197.112 | 7.449 | Unidentified | 0.015 | 0.075 | 0.398 |
| 447.134 | 6.534 | Unidentified | 0.041 | 0.121 | 0.383 |
| 145.106 | 0.953 | 4-Guanidinobutanamide | 0.028 | 0.100 | 0.368 |
| 114.046 | 6.538 | Unidentified | 0.016 | 0.076 | 0.302 |
| 140.000 | 7.520 | Unidentified | 0.014 | 0.071 | 0.302 |
| 148.079 | 4.298 | Unidentified | 0.023 | 0.092 | 0.289 |
| 346.158 | 6.541 | Unidentified | 0.022 | 0.088 | 0.283 |
| 358.298 | 20.278 | N-palmitoyl threonine | 0.042 | 0.122 | 0.270 |
| 175.120 | 1.047 | L-Arginine | 0.009 | 0.055 | 0.263 |
| 89.037 | 6.539 | Pyruvate | 0.040 | 0.121 | 0.204 |
| 132.102 | 2.014 | L-Isoleucine | 0.000 | 0.013 | 0.200 |
| 127.053 | 6.537 | Thymine | 0.026 | 0.097 | 0.192 |
| 70.074 | 1.961 | 1-Pyrroline | 0.015 | 0.072 | 0.135 |
| 120.501 | 4.296 | Unidentified | 0.030 | 0.104 | 0.096 |
| 175.088 | 3.096 | Unidentified | 0.000 | 0.013 | -0.149 |
| 133.030 | 1.785 | Unidentified | 0.049 | 0.130 | -0.149 |
| 96.080 | 1.849 | Unidentified | 0.041 | 0.121 | -0.159 |
| 997.361 | 0.762 | PIP2(16:0/18:1(11Z)) | 0.011 | 0.061 | -0.163 |
| 202.108 | 1.394 | (E)-1-(4-methylquinazolin-2(1H)-ylidene) guanidine | 0.002 | 0.027 | -0.175 |
| 140.107 | 3.271 | Unidentified | 0.042 | 0.122 | -0.193 |
| 152.033 | 1.700 | Unidentified | 0.000 | 0.013 | -0.193 |
| 123.107 | 2.228 | Unidentified | 0.024 | 0.093 | -0.204 |
| 543.154 | 0.745 | Unidentified | 0.023 | 0.090 | -0.210 |
| 321.144 | 6.739 | Unidentified | 0.047 | 0.128 | -0.214 |
| 256.081 | 0.791 | methyl 2-[(2,3-dihydroxybenzoyl) amino]-3-hydroxypropanoate | 0.038 | 0.116 | -0.221 |
| 375.995 | 1.654 | Unidentified | 0.023 | 0.092 | -0.222 |
| 128.107 | 3.259 | N-Cyclohexylformamide | 0.033 | 0.108 | -0.226 |
| 341.026 | 0.851 | Unidentified | 0.031 | 0.106 | -0.231 |
| 337.175 | 0.774 | Unidentified | 0.017 | 0.079 | -0.232 |
| 172.042 | 1.275 | Unidentified | 0.048 | 0.128 | -0.236 |
| 328.139 | 5.014 | PC(O-2:0/2:0) | 0.039 | 0.117 | -0.240 |
| 284.058 | 16.265 | N2-Acetyl-L-aminoadipyl-δ-phosphate | 0.016 | 0.077 | -0.251 |
| 145.048 | 4.997 | 3-hexenedioic acid | 0.011 | 0.062 | -0.252 |
| 103.054 | 5.014 | N-Formiminoglycine | 0.016 | 0.077 | -0.254 |
| 127.038 | 3.255 | Unidentified | 0.006 | 0.045 | -0.260 |
| 102.057 | 9.879 | Unidentified | 0.038 | 0.117 | -0.261 |
| 633.078 | 1.673 | Unidentified | 0.042 | 0.122 | -0.269 |
| 79.022 | 1.135 | Unidentified | 0.045 | 0.126 | -0.281 |
| 144.079 | 5.017 | Unidentified | 0.024 | 0.093 | -0.282 |
| 601.108 | 0.825 | Protoporphyrin IX | 0.031 | 0.106 | -0.285 |
| 174.092 | 5.029 | Unidentified | 0.010 | 0.058 | -0.293 |
| 134.998 | 1.192 | Malic acid | 0.001 | 0.016 | -0.296 |
| 132.082 | 5.010 | Unidentified | 0.041 | 0.122 | -0.296 |
| 343.027 | 0.856 | Unidentified | 0.004 | 0.033 | -0.305 |
| 246.112 | 5.034 | Asparaginyl-Hydroxyproline | 0.007 | 0.051 | -0.306 |
| 162.057 | 1.783 | Unidentified | 0.002 | 0.022 | -0.306 |
| 310.126 | 5.021 | Unidentified | 0.044 | 0.125 | -0.323 |
| 282.132 | 5.023 | Unidentified | 0.047 | 0.128 | -0.324 |
| 230.084 | 1.798 | Unidentified | 0.016 | 0.075 | -0.324 |
| 298.129 | 2.722 | Phenethylamine glucuronide | 0.045 | 0.126 | -0.324 |
| 95.050 | 2.221 | Phenol | 0.033 | 0.108 | -0.325 |
| 97.028 | 5.033 | Methaneselenol | 0.001 | 0.013 | -0.340 |
| 264.121 | 5.021 | 7-Mercaptoheptanoylthreonine | 0.025 | 0.094 | -0.341 |
| 261.023 | 18.642 | D-Galactose 6-sulfate | 0.042 | 0.122 | -0.343 |
| 303.135 | 6.743 | Unidentified | 0.044 | 0.125 | -0.343 |
| 398.987 | 0.896 | Unidentified | 0.045 | 0.126 | -0.345 |
| 362.037 | 16.238 | Unidentified | 0.018 | 0.079 | -0.346 |
| 191.082 | 16.230 | 2-Oxo-7-methylthioheptanoic acid | 0.005 | 0.038 | -0.347 |
| 116.105 | 3.190 | Unidentified | 0.024 | 0.094 | -0.358 |
| 505.037 | 1.662 | Thiamin triphosphate | 0.014 | 0.068 | -0.360 |
| 98.097 | 3.190 | Deoxycytosine | 0.040 | 0.119 | -0.362 |
| 375.097 | 16.253 | Unidentified | 0.003 | 0.031 | -0.362 |
| 458.692 | 17.498 | 7-methylguanosine 5'-diphosphate | 0.020 | 0.085 | -0.362 |
| 292.119 | 5.037 | Unidentified | 0.034 | 0.108 | -0.363 |
| 272.085 | 18.623 | Unidentified | 0.005 | 0.042 | -0.365 |
| 354.167 | 0.838 | Lys-Val-OH | 0.001 | 0.016 | -0.371 |
| 244.099 | 5.041 | Cytidine | 0.001 | 0.013 | -0.374 |
| 203.082 | 16.242 | Unidentified | 0.006 | 0.045 | -0.376 |
| 535.198 | 18.189 | 5''-Phosphoribostamycin | 0.000 | 0.004 | -0.376 |
| 162.078 | 6.751 | L-2-Aminoadipic acid | 0.025 | 0.094 | -0.386 |
| 290.126 | 3.565 | Unidentified | 0.018 | 0.080 | -0.394 |
| 126.066 | 1.803 | 5-Methylcytosine | 0.030 | 0.104 | -0.394 |
| 245.094 | 18.633 | D-Biotin | 0.035 | 0.110 | -0.412 |
| 643.225 | 19.440 | Unidentified | 0.008 | 0.053 | -0.420 |
| 101.004 | 1.135 | Sodium fluoroacetate | 0.008 | 0.055 | -0.424 |
| 203.052 | 1.032 | Unidentified | 0.001 | 0.015 | -0.426 |
| 420.064 | 6.747 | Unidentified | 0.032 | 0.107 | -0.428 |
| 257.095 | 18.646 | Unidentified | 0.018 | 0.081 | -0.433 |
| 139.004 | 1.562 | Unidentified | 0.042 | 0.122 | -0.439 |
| 344.135 | 2.720 | b-D-Glucopyranosiduronic acid | 0.019 | 0.081 | -0.441 |
| 308.114 | 2.737 | Cysteinyl-Tryptophan | 0.017 | 0.079 | -0.442 |
| 319.035 | 5.778 | Unidentified | 0.021 | 0.087 | -0.444 |
| 91.056 | 2.221 | Unidentified | 0.034 | 0.108 | -0.444 |
| 156.101 | 1.395 | Unidentified | 0.004 | 0.036 | -0.446 |
| 273.089 | 18.650 | Unidentified | 0.000 | 0.013 | -0.447 |
| 136.077 | 16.229 | N-Acetylarylamine | 0.018 | 0.080 | -0.449 |
| 189.071 | 16.249 | Unidentified | 0.018 | 0.081 | -0.450 |
| 194.080 | 2.701 | Phenylacetylglycine | 0.011 | 0.062 | -0.451 |
| 276.120 | 6.746 | Norophthalmic acid | 0.033 | 0.108 | -0.463 |
| 275.138 | 16.232 | Glutaminyl-Glutamine | 0.011 | 0.063 | -0.466 |
| 408.181 | 1.850 | Unidentified | 0.013 | 0.068 | -0.467 |
| 232.118 | 1.068 | Suberylglycine | 0.001 | 0.017 | -0.473 |
| 353.113 | 16.244 | Penicilloic G acid | 0.034 | 0.108 | -0.475 |
| 307.108 | 16.256 | Glutathione amide | 0.012 | 0.064 | -0.475 |
| 326.119 | 7.458 | Citalopram (propionic acid derivative) | 0.027 | 0.100 | -0.476 |
| 274.107 | 12.258 | L-Thyronine | 0.004 | 0.035 | -0.477 |
| 228.085 | 4.069 | L-Arogenate | 0.039 | 0.117 | -0.479 |
| 389.132 | 6.749 | TyrMe-Ala-OH | 0.044 | 0.125 | -0.482 |
| 197.102 | 2.896 | L-(+)-Arginine | 0.044 | 0.125 | -0.484 |
| 167.094 | 1.133 | N-Formyl-4-amino-5-aminomethyl-2-methylpyrimidine | 0.017 | 0.079 | -0.493 |
| 575.271 | 0.770 | Tetrahydrofolyl-[Glu](2) | 0.016 | 0.077 | -0.495 |
| 156.079 | 6.748 | Histidine | 0.009 | 0.055 | -0.495 |
| 124.074 | 5.778 | Unidentified | 0.010 | 0.058 | -0.496 |
| 220.081 | 6.735 | O-Succinyl-L-homoserine | 0.018 | 0.080 | -0.499 |
| 114.091 | 1.402 | Unidentified | 0.001 | 0.014 | -0.501 |
| 180.101 | 1.403 | Unidentified | 0.001 | 0.015 | -0.508 |
| 256.082 | 1.914 | Nicotinate D-ribonucleoside | 0.019 | 0.083 | -0.513 |
| 177.102 | 16.232 | Unidentified | 0.034 | 0.110 | -0.514 |
| 217.065 | 16.239 | Unidentified | 0.031 | 0.106 | -0.515 |
| 975.387 | 0.759 | Unidentified | 0.019 | 0.081 | -0.517 |
| 334.975 | 1.198 | Unidentified | 0.008 | 0.052 | -0.517 |
| 214.106 | 1.069 | Unidentified | 0.006 | 0.045 | -0.518 |
| 367.152 | 6.750 | Unidentified | 0.047 | 0.128 | -0.526 |
| 280.115 | 2.727 | Unidentified | 0.008 | 0.054 | -0.527 |
| 152.054 | 5.357 | Unidentified | 0.008 | 0.053 | -0.528 |
| 354.147 | 11.344 | Unidentified | 0.038 | 0.116 | -0.531 |
| 535.535 | 18.184 | 1-(8E-octadecenyl)-heptadecanoate | 0.010 | 0.058 | -0.537 |
| 355.066 | 18.653 | Phenolsulfonphthalein | 0.029 | 0.103 | -0.539 |
| 130.048 | 9.874 | Pyroglutamic acid | 0.014 | 0.071 | -0.542 |
| 331.127 | 6.761 | Unidentified | 0.044 | 0.125 | -0.547 |
| 258.133 | 2.437 | Unidentified | 0.032 | 0.107 | -0.556 |
| 168.061 | 3.542 | Unidentified | 0.010 | 0.058 | -0.560 |
| 238.071 | 1.920 | Unidentified | 0.007 | 0.051 | -0.564 |
| 309.128 | 0.790 | Unidentified | 0.031 | 0.106 | -0.564 |
| 142.086 | 1.408 | Unidentified | 0.008 | 0.053 | -0.565 |
| 143.056 | 1.404 | Unidentified | 0.009 | 0.055 | -0.570 |
| 128.053 | 16.230 | Unidentified | 0.033 | 0.108 | -0.576 |
| 481.172 | 16.229 | Tyr-TyrMe-OH | 0.021 | 0.087 | -0.578 |
| 280.139 | 1.403 | Unidentified | 0.000 | 0.012 | -0.588 |
| 284.110 | 1.362 | Glutamyl-Histidine | 0.030 | 0.106 | -0.589 |
| 168.079 | 6.753 | N-Trimethyl-2-aminoethylphosphonate | 0.006 | 0.046 | -0.593 |
| 263.122 | 16.226 | Nopalinic acid | 0.025 | 0.095 | -0.599 |
| 174.056 | 16.229 | Quinaldic acid | 0.031 | 0.106 | -0.601 |
| 290.161 | 5.440 | 3-Methylglutarylcarnitine | 0.023 | 0.090 | -0.602 |
| 90.054 | 2.340 | Alanine | 0.000 | 0.008 | -0.602 |
| 278.123 | 1.069 | S-(2-Methylpropionyl)-dihydrolipoamide-E | 0.001 | 0.018 | -0.610 |
| 460.151 | 1.038 | Phe-Trp-OH | 0.005 | 0.042 | -0.611 |
| 106.041 | 9.874 | 2-cyano-Pyrimidine | 0.040 | 0.121 | -0.613 |
| 176.055 | 9.874 | N-Formyl-L-glutamate | 0.033 | 0.108 | -0.619 |
| 534.863 | 18.177 | LysoPE(0:0/22:2(13Z,16Z)) | 0.001 | 0.017 | -0.634 |
| 161.068 | 1.405 | Unidentified | 0.000 | 0.008 | -0.635 |
| 123.046 | 2.221 | THIODIGLYCOL | 0.028 | 0.101 | -0.636 |
| 198.113 | 1.405 | Metanephrine | 0.000 | 0.006 | -0.638 |
| 410.140 | 16.029 | Unidentified | 0.026 | 0.096 | -0.640 |
| 120.045 | 9.883 | Aminomalonic acid | 0.036 | 0.114 | -0.644 |
| 634.741 | 16.356 | Cer(d18:2/23:0) | 0.021 | 0.087 | -0.647 |
| 302.119 | 1.350 | Unidentified | 0.009 | 0.055 | -0.656 |
| 613.188 | 1.398 | Unidentified | 0.008 | 0.053 | -0.659 |
| 244.121 | 1.406 | Glutaminyl-Proline | 0.004 | 0.035 | -0.668 |
| 160.095 | 1.401 | N-Isovalerylglycine | 0.000 | 0.013 | -0.670 |
| 369.075 | 19.950 | 2-Deoxy-4-O-[(2E)-3-(4-hydroxyphenyl)-2-propenoyl]-3-C-(methoxycarbonyl)pentaric acid | 0.045 | 0.126 | -0.675 |
| 260.113 | 1.069 | Unidentified | 0.002 | 0.022 | -0.679 |
| 262.130 | 1.401 | Unidentified | 0.001 | 0.015 | -0.697 |
| 569.208 | 0.785 | Leukotriene F4 | 0.017 | 0.079 | -0.697 |
| 383.026 | 18.621 | Unidentified | 0.029 | 0.103 | -0.702 |
| 132.986 | 0.961 | Unidentified | 0.016 | 0.077 | -0.704 |
| 183.091 | 6.753 | Unidentified | 0.038 | 0.116 | -0.707 |
| 112.051 | 1.268 | Cytosine | 0.026 | 0.096 | -0.711 |
| 80.048 | 1.845 | Unidentified | 0.021 | 0.087 | -0.714 |
| 216.124 | 1.399 | Unidentified | 0.001 | 0.017 | -0.717 |
| 283.069 | 0.720 | 9-Riburonosylhypoxanthine | 0.018 | 0.079 | -0.719 |
| 223.076 | 4.779 | L-Cystathionine | 0.002 | 0.022 | -0.740 |
| 423.194 | 1.972 | Unidentified | 0.001 | 0.016 | -0.749 |
| 360.093 | 13.854 | Unidentified | 0.032 | 0.107 | -0.751 |
| 234.135 | 1.398 | Unidentified | 0.001 | 0.016 | -0.761 |
| 274.090 | 1.944 | Pentose + Proline | 0.001 | 0.013 | -0.762 |
| 736.214 | 4.295 | Unidentified | 0.017 | 0.077 | -0.778 |
| 330.058 | 1.959 | cAMP | 0.007 | 0.047 | -0.782 |
| 386.212 | 19.422 | Unidentified | 0.020 | 0.085 | -0.784 |
| 203.151 | 0.898 | N,N-Dimethylarginine | 0.002 | 0.022 | -0.785 |
| 371.144 | 4.847 | 4beta-Hydroxyobovatachromene | 0.000 | 0.006 | -0.791 |
| 78.035 | 1.559 | Cysteamine | 0.006 | 0.045 | -0.799 |
| 218.065 | 6.752 | 2-(Hydroxymethyl)-3-(acetamidomethylene)succinate | 0.007 | 0.047 | -0.808 |
| 308.169 | 5.486 | Unidentified | 0.004 | 0.035 | -0.817 |
| 312.212 | 1.782 | Unidentified | 0.049 | 0.131 | -0.821 |
| 192.054 | 9.894 | Unidentified | 0.030 | 0.104 | -0.821 |
| 687.212 | 19.401 | Unidentified | 0.001 | 0.013 | -0.822 |
| 113.107 | 5.786 | 2-Imino-4-methylpiperidine | 0.009 | 0.055 | -0.837 |
| 322.087 | 1.067 | Deoxy-5-methylcytidylate | 0.014 | 0.068 | -0.852 |
| 384.022 | 18.618 | Unidentified | 0.005 | 0.043 | -0.868 |
| 374.060 | 1.833 | Unidentified | 0.000 | 0.013 | -0.868 |
| 151.062 | 5.364 | 1-Methylhypoxanthine | 0.002 | 0.022 | -0.872 |
| 316.078 | 1.070 | Unidentified | 0.011 | 0.060 | -0.876 |
| 492.139 | 5.771 | Unidentified | 0.047 | 0.128 | -0.877 |
| 518.237 | 1.366 | PS(18:4(6Z,9Z,12Z,15Z)/0:0) | 0.009 | 0.055 | -0.879 |
| 354.075 | 4.780 | Unidentified | 0.004 | 0.035 | -0.905 |
| 516.224 | 1.070 | Unidentified | 0.005 | 0.039 | -0.909 |
| 146.028 | 1.178 | Unidentified | 0.024 | 0.093 | -0.916 |
| 313.105 | 9.874 | Thr-Ala-OH | 0.022 | 0.089 | -0.938 |
| 300.103 | 1.069 | 8-hydroxy Guanosine | 0.004 | 0.035 | -0.961 |
| 254.099 | 8.874 | Dihydroferuloylglycine | 0.030 | 0.104 | -0.976 |
| 340.089 | 4.301 | 6-Hydroxy-5-methoxyindole glucuronide | 0.000 | 0.008 | -0.978 |
| 393.071 | 0.963 | 5,7,3',4',5'-Pentahydroxy-3,6,8-trimethoxyflavone | 0.000 | 0.008 | -0.984 |
| 560.189 | 1.070 | Unidentified | 0.017 | 0.079 | -0.999 |
| 314.084 | 1.945 | Unidentified | 0.000 | 0.004 | -1.003 |
| 524.077 | 1.377 | Unidentified | 0.009 | 0.055 | -1.011 |
| 160.043 | 14.016 | Unidentified | 0.023 | 0.091 | -1.020 |
| 208.099 | 8.873 | 4-(2-Aminophenyl)-2,4-dioxobutanoic acid | 0.036 | 0.114 | -1.022 |
| 267.096 | 9.892 | Unidentified | 0.013 | 0.068 | -1.043 |
| 241.030 | 14.023 | L-Cystine | 0.033 | 0.108 | -1.051 |
| 263.094 | 5.756 | Unidentified | 0.016 | 0.077 | -1.058 |
| 612.187 | 1.396 | Unidentified | 0.004 | 0.035 | -1.066 |
| 490.210 | 0.770 | Unidentified | 0.004 | 0.035 | -1.066 |
| 538.202 | 1.069 | Unidentified | 0.014 | 0.068 | -1.070 |
| 440.151 | 15.711 | Trp-Leu-OH | 0.014 | 0.071 | -1.093 |
| 786.205 | 6.741 | Flavin adenine dinucleotide (FAD) | 0.009 | 0.055 | -1.112 |
| 288.068 | 14.019 | Unidentified | 0.013 | 0.067 | -1.119 |
| 163.089 | 4.132 | Unidentified | 0.005 | 0.039 | -1.124 |
| 402.128 | 3.500 | HoPhe-Asn-OH | 0.009 | 0.055 | -1.135 |
| 94.065 | 4.138 | Unidentified | 0.003 | 0.032 | -1.137 |
| 232.002 | 4.191 | Unidentified | 0.019 | 0.083 | -1.155 |
| 552.182 | 1.974 | N-Acetyl-N6,N6,O-tridemethylpuromycin-5'-phosphate | 0.000 | 0.011 | -1.160 |
| 280.231 | 1.408 | Linoleamide | 0.003 | 0.030 | -1.175 |
| 77.039 | 14.015 | Unidentified | 0.004 | 0.033 | -1.176 |
| 353.315 | 16.232 | PGE2 | 0.048 | 0.128 | -1.183 |
| 146.062 | 4.136 | Unidentified | 0.006 | 0.044 | -1.220 |
| 103.054 | 14.021 | Unidentified | 0.003 | 0.031 | -1.228 |
| 267.146 | 3.039 | Unidentified | 0.023 | 0.091 | -1.237 |
| 220.096 | 14.010 | Unidentified | 0.037 | 0.115 | -1.244 |
| 355.118 | 18.623 | Unidentified | 0.040 | 0.119 | -1.247 |
| 784.562 | 22.733 | Arachidonoyl thio-PC | 0.002 | 0.026 | -1.277 |
| 581.256 | 1.371 | Unidentified | 0.012 | 0.064 | -1.278 |
| 344.238 | 2.705 | Unidentified | 0.036 | 0.114 | -1.293 |
| 416.090 | 14.019 | Unidentified | 0.025 | 0.095 | -1.323 |
| 219.097 | 1.135 | Glutamylalanine | 0.001 | 0.019 | -1.361 |
| 136.078 | 4.131 | L-Homocysteine | 0.003 | 0.031 | -1.368 |
| 290.043 | 4.191 | Unidentified | 0.047 | 0.128 | -1.385 |
| 221.066 | 16.053 | Unidentified | 0.025 | 0.094 | -1.430 |
| 515.172 | 15.768 | TyrMe-Nap-OH | 0.003 | 0.030 | -1.438 |
| 281.210 | 1.404 | 12S-hydroxy-5Z,8E,10E-heptadecatrienoic acid | 0.002 | 0.026 | -1.449 |
| 261.146 | 7.254 | L-gamma-glutamyl-L-isoleucine | 0.001 | 0.019 | -1.460 |
| 337.021 | 14.018 | Unidentified | 0.013 | 0.067 | -1.488 |
| 326.219 | 2.700 | Farnesylcysteine | 0.034 | 0.110 | -1.489 |
| 209.093 | 4.132 | Kynurenine | 0.002 | 0.022 | -1.501 |
| 365.200 | 1.007 | Unidentified | 0.003 | 0.030 | -1.525 |
| 342.086 | 16.069 | Unidentified | 0.006 | 0.045 | -1.525 |
| 192.064 | 4.132 | 5-Hydroxyindoleacetic acid | 0.001 | 0.016 | -1.534 |
| 166.086 | 14.016 | L-Phenylalanine | 0.004 | 0.035 | -1.558 |
| 582.165 | 1.068 | Unidentified | 0.003 | 0.031 | -1.571 |
| 280.312 | 1.409 | Unidentified | 0.001 | 0.013 | -1.593 |
| 891.311 | 1.402 | dipalmitoyl phosphatidylinositol 3-phosphate | 0.002 | 0.026 | -1.611 |
| 705.228 | 16.255 | Unidentified | 0.045 | 0.126 | -1.632 |
| 334.140 | 10.201 | Unidentified | 0.000 | 0.006 | -1.646 |
| 262.321 | 1.402 | Unidentified | 0.002 | 0.022 | -1.668 |
| 295.129 | 8.362 | Glutamylphenylalanine | 0.001 | 0.016 | -1.679 |
| 262.214 | 1.401 | Unidentified | 0.003 | 0.028 | -1.689 |
| 120.079 | 14.016 | L-Threonine | 0.006 | 0.045 | -1.723 |
| 605.175 | 1.935 | SP1 | 0.000 | 0.005 | -1.761 |
| 192.101 | 14.021 | Unidentified | 0.009 | 0.055 | -1.842 |
| 238.108 | 14.016 | N-lactoyl-phenylalanine | 0.009 | 0.055 | -1.852 |
| 577.221 | 1.068 | Formononetin 7-O-rutinoside | 0.012 | 0.065 | -1.890 |
| 279.097 | 4.456 | gamma-Glutamylmethionine | 0.008 | 0.054 | -1.903 |
| 441.152 | 5.224 | (+)-12a-Hydroxyerythynone | 0.000 | 0.010 | -1.945 |
| 303.068 | 5.293 | Unidentified | 0.001 | 0.016 | -2.014 |
| 575.129 | 14.016 | Unidentified | 0.018 | 0.080 | -2.047 |
| 277.120 | 12.502 | Unidentified | 0.002 | 0.022 | -2.050 |
| 260.091 | 14.014 | Unidentified | 0.032 | 0.107 | -2.150 |
| 289.120 | 8.997 | Unidentified | 0.009 | 0.055 | -2.325 |
| 381.147 | 1.646 | 2-Methoxyestrone 3-sulfate | 0.001 | 0.019 | -2.548 |
| 528.120 | 14.018 | Unidentified | 0.014 | 0.068 | -2.627 |
| 408.105 | 4.195 | S-Succinylglutathione | 0.007 | 0.051 | -2.924 |

**Table S7.** Metabolic Pathway Enrichment in SAECs Exposed to 5 µg/mL PM Collected from the Control Room.

| Pathway Name | Pathway Total | Total Hits | Significant Hits | Expected | P(Gamma) | AdjP.Gamma | Pathway Number | Enrichment Factor |
| --- | --- | --- | --- | --- | --- | --- | --- | --- |
| Ascorbate and aldarate metabolism | 8 | 2 | 1 | 0.204 | 0.002 | 0.026 | P4 | 4.906 |
| Histidine metabolism | 16 | 1 | 1 | 0.204 | 0.002 | 0.026 | P5 | 4.906 |
| Butanoate metabolism | 15 | 3 | 2 | 0.408 | 0.003 | 0.036 | P9 | 4.906 |
| Valine, leucine, and isoleucine degradation | 35 | 8 | 5 | 1.121 | 0.003 | 0.035 | P6 | 4.460 |
| Purine metabolism | 65 | 11 | 5 | 1.223 | 0.001 | 0.015 | P1 | 4.089 |
| Cysteine and methionine metabolism | 33 | 8 | 3 | 1.121 | 0.001 | 0.018 | P2 | 2.676 |
| Valine, leucine, and isoleucine biosynthesis | 8 | 6 | 3 | 1.121 | 0.003 | 0.035 | P7 | 2.676 |
| Pantothenate and CoA biosynthesis | 17 | 5 | 2 | 0.815 | 0.002 | 0.026 | P3 | 2.453 |
| Synthesis and degradation of ketone bodies | 5 | 2 | 1 | 0.408 | 0.003 | 0.036 | P8 | 2.453 |
| Propanoate metabolism | 19 | 5 | 2 | 0.815 | 0.008 | 0.058 | P13 | 2.453 |
| beta-Alanine metabolism | 21 | 3 | 1 | 0.510 | 0.004 | 0.040 | P10 | 1.963 |
| Aminoacyl-tRNA biosynthesis | 22 | 12 | 3 | 2.038 | 0.011 | 0.058 | P18 | 1.472 |
| Pyrimidine metabolism | 39 | 5 | 1 | 0.713 | 0.007 | 0.053 | P12 | 1.402 |
| Phenylalanine metabolism | 10 | 8 | 2 | 1.529 | 0.005 | 0.044 | P11 | 1.308 |
| Glycine, serine, and threonine metabolism | 30 | 5 | 1 | 0.917 | 0.010 | 0.058 | P15 | 1.090 |
| Arginine and proline metabolism | 37 | 8 | 1 | 0.917 | 0.010 | 0.058 | P16 | 1.090 |
| Drug metabolism - cytochrome P450 | 43 | 7 | 1 | 0.917 | 0.010 | 0.058 | P17 | 1.090 |
| Tyrosine metabolism | 42 | 13 | 2 | 1.936 | 0.009 | 0.058 | P14 | 1.033 |
| Alanine, aspartate, and glutamate metabolism | 28 | 5 | 1 | 1.019 | 0.013 | 0.058 | P19 | 0.981 |

**Table S8.** Metabolic Pathway Enrichment in SAECs Exposed to 5 µg/mL PM Collected from the Printer Room During Printing with ABS Filaments.

| Pathway Name | Pathway Total | Total Hits | Significant Hits | Expected | P(Gamma) | AdjP.Gamma | Pathway Number | Enrichment Factor |
| --- | --- | --- | --- | --- | --- | --- | --- | --- |
| Galactose metabolism | 27 | 9 | 7 | 0.293 | 0.002 | 0.025 | P11 | 23.892 |
| Glycolysis or Gluconeogenesis | 23 | 2 | 2 | 0.147 | 0.001 | 0.021 | P2 | 13.652 |
| Fructose and mannose metabolism | 20 | 4 | 3 | 0.293 | 0.002 | 0.025 | P10 | 10.239 |
| Amino sugar and nucleotide sugar metabolism | 35 | 6 | 4 | 0.439 | 0.003 | 0.025 | P17 | 9.101 |
| Ascorbate and aldarate metabolism | 8 | 2 | 2 | 0.293 | 0.001 | 0.013 | P1 | 6.826 |
| Caffeine metabolism | 10 | 3 | 2 | 0.293 | 0.002 | 0.025 | P12 | 6.826 |
| Starch and sucrose metabolism | 13 | 3 | 2 | 0.293 | 0.002 | 0.025 | P13 | 6.826 |
| Inositol phosphate metabolism | 21 | 1 | 1 | 0.147 | 0.001 | 0.021 | P3 | 6.826 |
| Phosphatidylinositol signaling system | 17 | 1 | 1 | 0.147 | 0.001 | 0.021 | P4 | 6.826 |
| Vitamin B6 metabolism | 9 | 2 | 1 | 0.293 | 0.002 | 0.025 | P14 | 3.413 |
| beta-Alanine metabolism | 21 | 3 | 2 | 0.732 | 0.001 | 0.021 | P5 | 2.730 |
| Pyrimidine metabolism | 39 | 5 | 2 | 1.026 | 0.002 | 0.025 | P15 | 1.950 |
| Valine, leucine, and isoleucine degradation | 35 | 8 | 3 | 1.612 | 0.001 | 0.022 | P7 | 1.862 |
| Valine, leucine and isoleucine biosynthesis | 8 | 6 | 3 | 1.612 | 0.001 | 0.022 | P8 | 1.862 |
| Phenylalanine metabolism | 10 | 8 | 4 | 2.198 | 0.001 | 0.021 | P6 | 1.820 |
| Purine metabolism | 65 | 11 | 3 | 1.758 | 0.001 | 0.023 | P9 | 1.706 |
| Aminoacyl-tRNA biosynthesis | 22 | 12 | 4 | 2.930 | 0.002 | 0.025 | P16 | 1.365 |
| Phenylalanine, tyrosine, and tryptophan biosynthesis | 4 | 3 | 1 | 0.879 | 0.009 | 0.045 | P20 | 1.138 |
| Tyrosine metabolism | 42 | 13 | 3 | 2.783 | 0.006 | 0.038 | P19 | 1.078 |
| Arginine and proline metabolism | 37 | 8 | 1 | 1.319 | 0.003 | 0.025 | P18 | 0.758 |

**Table S9.** Metabolic Pathway Enrichment in SAECs Exposed to 5 µg/mL PM Collected from the Printer Room During Printing with PLA Filaments.

| Pathway Name | Pathway Total | Total Hits | Significant Hits | Expected | P(Gamma) | AdjP.Gamma | Pathway Number | Enrichment Factor |
| --- | --- | --- | --- | --- | --- | --- | --- | --- |
| Retinol metabolism | 16 | 3 | 3 | 0.160 | 0.001 | 0.022 | P3 | 18.839 |
| Fatty acid biosynthesis | 10 | 1 | 1 | 0.160 | 0.001 | 0.022 | P1 | 6.280 |
| One carbon pool by folate | 9 | 1 | 1 | 0.160 | 0.001 | 0.022 | P2 | 6.280 |
| Ascorbate and aldarate metabolism | 8 | 2 | 1 | 0.318 | 0.002 | 0.032 | P6 | 3.140 |
| Caffeine metabolism | 10 | 3 | 1 | 0.318 | 0.002 | 0.032 | P7 | 3.140 |
| Folate biosynthesis | 24 | 4 | 1 | 0.478 | 0.003 | 0.035 | P11 | 2.093 |
| Pyrimidine metabolism | 39 | 5 | 2 | 1.115 | 0.002 | 0.032 | P8 | 1.794 |
| Purine metabolism | 65 | 11 | 3 | 1.911 | 0.002 | 0.032 | P5 | 1.570 |
| Pantothenate and CoA biosynthesis | 17 | 5 | 2 | 1.274 | 0.003 | 0.033 | P10 | 1.570 |
| Tyrosine metabolism | 42 | 13 | 4 | 3.026 | 0.003 | 0.033 | P9 | 1.322 |
| Phenylalanine, tyrosine, and tryptophan biosynthesis | 4 | 3 | 1 | 0.955 | 0.002 | 0.028 | P4 | 1.047 |
| Arginine and proline metabolism | 37 | 8 | 1 | 1.433 | 0.004 | 0.036 | P12 | 0.698 |

|  |  |  |  |  |  |  |  |  |  |  |  |  |  |  |  |
| --- | --- | --- | --- | --- | --- | --- | --- | --- | --- | --- | --- | --- | --- | --- | --- |
|  |  |  |  |  |  |  |  |  |  |  |  |  |  |  |  |
|  |  |  |  |  |  |  |  |  |  |  |  |  |  |  |  |
|  |  |  |  |  |  |  |  |  |  |  |  |  |  |  |  |
|  |  |  |  |  |  |  |  |  |  |  |  |  |  |  |  |
|  |  |  |  |  |  |  |  |  |  |  |  |  |  |  |  |
|  |  |  |  |  |  |  |  |  |  |  |  |  |  |  |  |
|  |  |  |  |  |  |  |  |  |  |  |  |  |  |  |  |
|  |  |  |  |  |  |  |  |  |  |  |  |  |  |  |  |
|  |  |  |  |  |  |  |  |  |  |  |  |  |  |  |  |
|  |  |  |  |  |  |  |  |  |  |  |  |  |  |  |  |
|  |  |  |  |  |  |  |  |  |  |  |  |  |  |  |  |
|  |  |  |  |  |  |  |  |  |  |  |  |  |  |  |  |
|  |  |  |  |  |  |  |  |  |  |  |  |  |  |  |  |
|  |  |  |  |  |  |  |  |  |  |  |  |  |  |  |  |
|  |  |  |  |  |  |  |  |  |  |  |  |  |  |  |  |
|  |  |  |  |  |  |  |  |  |  |  |  |  |  |  |  |
|  |  |  |  |  |  |  |  |  |  |  |  |  |  |  |  |
|  |  |  |  |  |  |  |  |  |  |  |  |  |  |  |  |
|  |  |  |  |  |  |  |  |  |  |  |  |  |  |  |  |
|  |  |  |  |  |  |  |  |  |  |  |  |  |  |  |  |

**Table S10.** Metabolic Pathway Enrichment in SAECs Exposed to 10 µg/mL PM Collected from the Control Room.

| Pathway Name | Pathway Total | Total Hits | Significant Hits | Expected | P(Gamma) | AdjP.Gamma | Pathway Number | Enrichment Factor |
| --- | --- | --- | --- | --- | --- | --- | --- | --- |
| Glutathione metabolism | 19 | 1 | 1 | 0.159 | 0.001 | 0.018 | P3 | 6.308 |
| Purine metabolism | 65 | 11 | 4 | 0.872 | 0.001 | 0.014 | P1 | 4.587 |
| D-Glutamine and D-glutamate metabolism | 6 | 3 | 1 | 0.238 | 0.002 | 0.020 | P5 | 4.205 |
| Pantothenate and CoA biosynthesis | 17 | 5 | 2 | 0.555 | 0.001 | 0.016 | P2 | 3.604 |
| Valine, leucine, and isoleucine biosynthesis | 8 | 7 | 3 | 0.951 | 0.002 | 0.020 | P6 | 3.154 |
| Caffeine metabolism | 10 | 4 | 1 | 0.317 | 0.002 | 0.021 | P7 | 3.154 |
| Aminoacyl-tRNA biosynthesis | 22 | 12 | 4 | 1.506 | 0.002 | 0.019 | P4 | 2.656 |
| beta-Alanine metabolism | 21 | 4 | 1 | 0.396 | 0.003 | 0.024 | P8 | 2.523 |
| Arginine and proline metabolism | 37 | 8 | 2 | 0.793 | 0.008 | 0.041 | P12 | 2.523 |
| Pyrimidine metabolism | 39 | 6 | 1 | 0.476 | 0.004 | 0.026 | P9 | 2.103 |
| Valine, leucine, and isoleucine degradation | 35 | 9 | 2 | 1.110 | 0.015 | 0.041 | P15 | 1.802 |
| Glycine, serine, and threonine metabolism | 30 | 6 | 1 | 0.713 | 0.007 | 0.041 | P10 | 1.402 |
| Cysteine and methionine metabolism | 33 | 7 | 1 | 0.713 | 0.007 | 0.041 | P11 | 1.402 |
| Drug metabolism - cytochrome P450 | 43 | 6 | 1 | 0.793 | 0.008 | 0.041 | P13 | 1.262 |
| Tryptophan metabolism | 41 | 18 | 2 | 1.982 | 0.010 | 0.041 | P14 | 1.009 |

**Table S11.** Metabolic Pathway Enrichment in SAECs Exposed to 10 µg/mL PM Collected from the Printer Room During Printing with ABS Filaments.

| Pathway Name | Pathway Total | Total Hits | Significant Hits | Expected | P(Gamma) | AdjP.Gamma | Pathway Number | Enrichment Factor |
| --- | --- | --- | --- | --- | --- | --- | --- | --- |
| Purine metabolism | 65 | 11 | 3 | 0.671 | 0.001 | 0.006 | P1 | 4.473 |
| beta-Alanine metabolism | 21 | 4 | 1 | 0.305 | 0.002 | 0.013 | P3 | 3.280 |
| Pantothenate and CoA biosynthesis | 17 | 5 | 1 | 0.427 | 0.003 | 0.014 | P5 | 2.343 |
| Cysteine and methionine metabolism | 33 | 7 | 1 | 0.549 | 0.004 | 0.014 | P6 | 1.822 |
| Arginine and proline metabolism | 37 | 8 | 1 | 0.610 | 0.001 | 0.008 | P2 | 1.640 |
| Tyrosine metabolism | 42 | 13 | 2 | 1.220 | 0.003 | 0.014 | P4 | 1.640 |
| Phenylalanine metabolism | 10 | 8 | 1 | 0.915 | 0.010 | 0.020 | P7 | 1.093 |
| Aminoacyl-tRNA biosynthesis | 22 | 12 | 1 | 1.159 | 0.016 | 0.020 | P8 | 0.863 |

**Table S12.** Metabolic Pathway Enrichment in SAECs Exposed to 10 µg/mL PM Collected from the Printer Room During Printing with PLA Filaments.

| Pathway Name | Pathway Total | Total Hits | Significant Hits | Expected | P(Gamma) | AdjP.Gamma | Pathway Number | Enrichment Factor |
| --- | --- | --- | --- | --- | --- | --- | --- | --- |
| Galactose metabolism | 27 | 9 | 7 | 0.171 | 0.001117 | 0.021 | P10 | 41.000 |
| Glycolysis or Gluconeogenesis | 23 | 2 | 2 | 0.085 | 0.000803 | 0.020 | P4 | 23.429 |
| Fructose and mannose metabolism | 20 | 4 | 3 | 0.171 | 0.001117 | 0.021 | P9 | 17.572 |
| Amino sugar and nucleotide sugar metabolism | 35 | 6 | 4 | 0.256 | 0.001522 | 0.021 | P17 | 15.619 |
| Phosphatidylinositol signaling system | 17 | 1 | 1 | 0.085 | 0.000803 | 0.020 | P7 | 11.714 |
| Inositol phosphate metabolism | 21 | 1 | 1 | 0.085 | 0.000803 | 0.020 | P6 | 11.714 |
| Ascorbate and aldarate metabolism | 8 | 1 | 1 | 0.085 | 0.000803 | 0.020 | P5 | 11.714 |
| Starch and sucrose metabolism | 13 | 3 | 2 | 0.171 | 0.001117 | 0.021 | P13 | 11.714 |
| Caffeine metabolism | 10 | 4 | 2 | 0.341 | 0.002033 | 0.021 | P18 | 5.857 |
| Vitamin B6 metabolism | 9 | 2 | 1 | 0.171 | 0.001117 | 0.021 | P14 | 5.857 |
| Glutathione metabolism | 19 | 1 | 1 | 0.171 | 0.001117 | 0.021 | P12 | 5.857 |
| Selenocompound metabolism | 16 | 1 | 1 | 0.171 | 0.001117 | 0.021 | P11 | 5.857 |
| beta-Alanine metabolism | 21 | 4 | 2 | 0.427 | 0.000718 | 0.019 | P2 | 4.686 |
| Pyrimidine metabolism | 39 | 6 | 2 | 0.512 | 0.000796 | 0.020 | P3 | 3.905 |
| D-Glutamine and D-glutamate metabolism | 6 | 3 | 1 | 0.256 | 0.001522 | 0.021 | P16 | 3.905 |
| Pantothenate and CoA biosynthesis | 17 | 5 | 2 | 0.598 | 0.000894 | 0.020 | P8 | 3.347 |
| Purine metabolism | 65 | 11 | 3 | 0.939 | 0.000706 | 0.019 | P1 | 3.195 |
| Arginine and proline metabolism | 37 | 8 | 2 | 0.854 | 0.00816 | 0.040 | P25 | 2.343 |
| Drug metabolism - cytochrome P450 | 43 | 6 | 2 | 0.854 | 0.001331 | 0.021 | P15 | 2.343 |
| Valine, leucine, and isoleucine biosynthesis | 8 | 7 | 2 | 1.024 | 0.011667 | 0.040 | P26 | 1.952 |
| Aminoacyl-tRNA biosynthesis | 22 | 12 | 3 | 1.622 | 0.005041 | 0.040 | P20 | 1.850 |
| Valine, leucine, and isoleucine degradation | 35 | 9 | 2 | 1.195 | 0.016012 | 0.040 | P27 | 1.674 |
| Alanine, aspartate, and glutamate metabolism | 28 | 5 | 1 | 0.683 | 0.005442 | 0.040 | P21 | 1.464 |
| Propanoate metabolism | 19 | 5 | 1 | 0.768 | 0.006706 | 0.040 | P24 | 1.302 |
| Glycine, serine, and threonine metabolism | 30 | 6 | 1 | 0.768 | 0.006706 | 0.040 | P23 | 1.302 |
| Tyrosine metabolism | 42 | 13 | 2 | 1.707 | 0.005812 | 0.040 | P22 | 1.171 |
| Tryptophan metabolism | 41 | 18 | 2 | 2.134 | 0.002643 | 0.024 | P19 | 0.937 |
